# Widening the lens of population-based health research to climate change impacts and adaptation: the climate change and health evaluation and response system (CHEERS)

**DOI:** 10.3389/fpubh.2023.1153559

**Published:** 2023-05-25

**Authors:** Sandra Barteit, Ali Sié, Pascal Zabré, I Traoré, Windpanga Aristide Ouédraogo, Valentin Boudo, Stephen Munga, Sammy Khagayi, David Obor, Erick Muok, Jonas Franke, Maximilian Schwarz, Klaus Blass, Tin Tin Su, Till Bärnighausen, Osman Sankoh, Rainer Sauerborn

**Affiliations:** ^1^Heidelberg Institute of Global Health (HIGH), Faculty of Medicine and University Hospital, Heidelberg University, Heidelberg, Germany; ^2^Centre de Recherche en Santé de Nouna, Nouna, Burkina Faso; ^3^Kenya Medical Research Institute, Kisumu, Kenya; ^4^Remote Sensing Solutions, Munich, Germany; ^5^South East Asia Community Observatory (SEACO) and Global Public Health, Jeffrey Cheah School of Medicine and Health Sciences, Monash University, Bandar Sunway, Malaysia; ^6^Africa Health Research Institute (AHRI), KwaZulu-Natal, South Africa; ^7^Harvard Center for Population and Development Studies, Cambridge, MA, United States; ^8^Statistics Sierra Leone, Freetown, Sierra Leone; ^9^School of Public Health, Faculty of Health Sciences, University of the Witwatersrand, Johannesburg, South Africa

**Keywords:** health impacts, public health surveillance, climate change, digital health, low and middle-income country, climate change and health, response system

## Abstract

**Background:**

Climate change significantly impacts health in low-and middle-income countries (LMICs), exacerbating vulnerabilities. Comprehensive data for evidence-based research and decision-making is crucial but scarce. Health and Demographic Surveillance Sites (HDSSs) in Africa and Asia provide a robust infrastructure with longitudinal population cohort data, yet they lack climate-health specific data. Acquiring this information is essential for understanding the burden of climate-sensitive diseases on populations and guiding targeted policies and interventions in LMICs to enhance mitigation and adaptation capacities.

**Objective:**

The objective of this research is to develop and implement the Change and Health Evaluation and Response System (CHEERS) as a methodological framework, designed to facilitate the generation and ongoing monitoring of climate change and health-related data within existing Health and Demographic Surveillance Sites (HDSSs) and comparable research infrastructures.

**Methods:**

CHEERS uses a multi-tiered approach to assess health and environmental exposures at the individual, household, and community levels, utilizing digital tools such as wearable devices, indoor temperature and humidity measurements, remotely sensed satellite data, and 3D-printed weather stations. The CHEERS framework utilizes a graph database to efficiently manage and analyze diverse data types, leveraging graph algorithms to understand the complex interplay between health and environmental exposures.

**Results:**

The Nouna CHEERS site, established in 2022, has yielded significant preliminary findings. By using remotely-sensed data, the site has been able to predict crop yield at a household level in Nouna and explore the relationships between yield, socioeconomic factors, and health outcomes. The feasibility and acceptability of wearable technology have been confirmed in rural Burkina Faso for obtaining individual-level data, despite the presence of technical challenges. The use of wearables to study the impact of extreme weather on health has shown significant effects of heat exposure on sleep and daily activity, highlighting the urgent need for interventions to mitigate adverse health consequences.

**Conclusion:**

Implementing the CHEERS in research infrastructures can advance climate change and health research, as large and longitudinal datasets have been scarce for LMICs. This data can inform health priorities, guide resource allocation to address climate change and health exposures, and protect vulnerable communities in LMICs from these exposures.

## 1. Introduction

### 1.1. The present landscape of health and demographic surveillance systems (HDSSs) – standardized, longitudinal mortality data across 56 sites spanning 60 years

In many countries across sub-Sahara Africa, South and South-East Asia, reliable data on vital events and cause of deaths remains scarce (1–3). Often, this information is generated through routine reporting systems within the public health sector, which possess inherent biases. Users of modern health services do not accurately represent the population, as access is constrained by factors such as financial affordability, geographical accessibility, cultural acceptability, and other limitations. Furthermore, data from the widely utilized private healthcare sector is not included in these systems, leading to unrecorded vital events, such as births, deaths, and migrations, and biased estimates of the population’s disease burden. For example, a significant number of deaths occur in homes, complicating the reliable attribution of a cause of death. Although the Demographic and Health Survey (DHS) provides high-quality data, cross-sectional surveys offer only a snapshot of a country or population’s health profile (4, 5). While nationally and often sub-nationally representative, the lengthy intervals between DHSs, typically 5 years, cause the collected information to become outdated rapidly. The cross-sectional survey format also limits its analytical scope, hindering the evaluation of intervention effects and medium-to long-term health trends. National census data share similar constraints.

Health and demographic surveillance systems (HDSSs) address the limitations of cross-sectional surveys in several ways:

- HDSSs encompass long-term, dynamic, whole-population cohorts with up to 62 years of continuous data, covering an average of 75,000 people residing in a defined geographic region (1) (refer to Supplementary Appendix 1 for an overview of HDSS sites, including population, villages, site size (km^2^), and start year).
- Within these populations, HDSSs collect both denominator and numerator data, enabling the use of relative precision through person-time measurements.
- Demographic events, including deaths, births, and migrations, are recorded.
- A verbal autopsy adhering to the latest World Health Organization (WHO) Verbal Autopsy specification is conducted using a set of validated questions to determine the cause of death occurring outside of healthcare facilities (which are often limited) or physical autopsies (which are frequently culturally inappropriate and infeasible).

**Figure 1:**
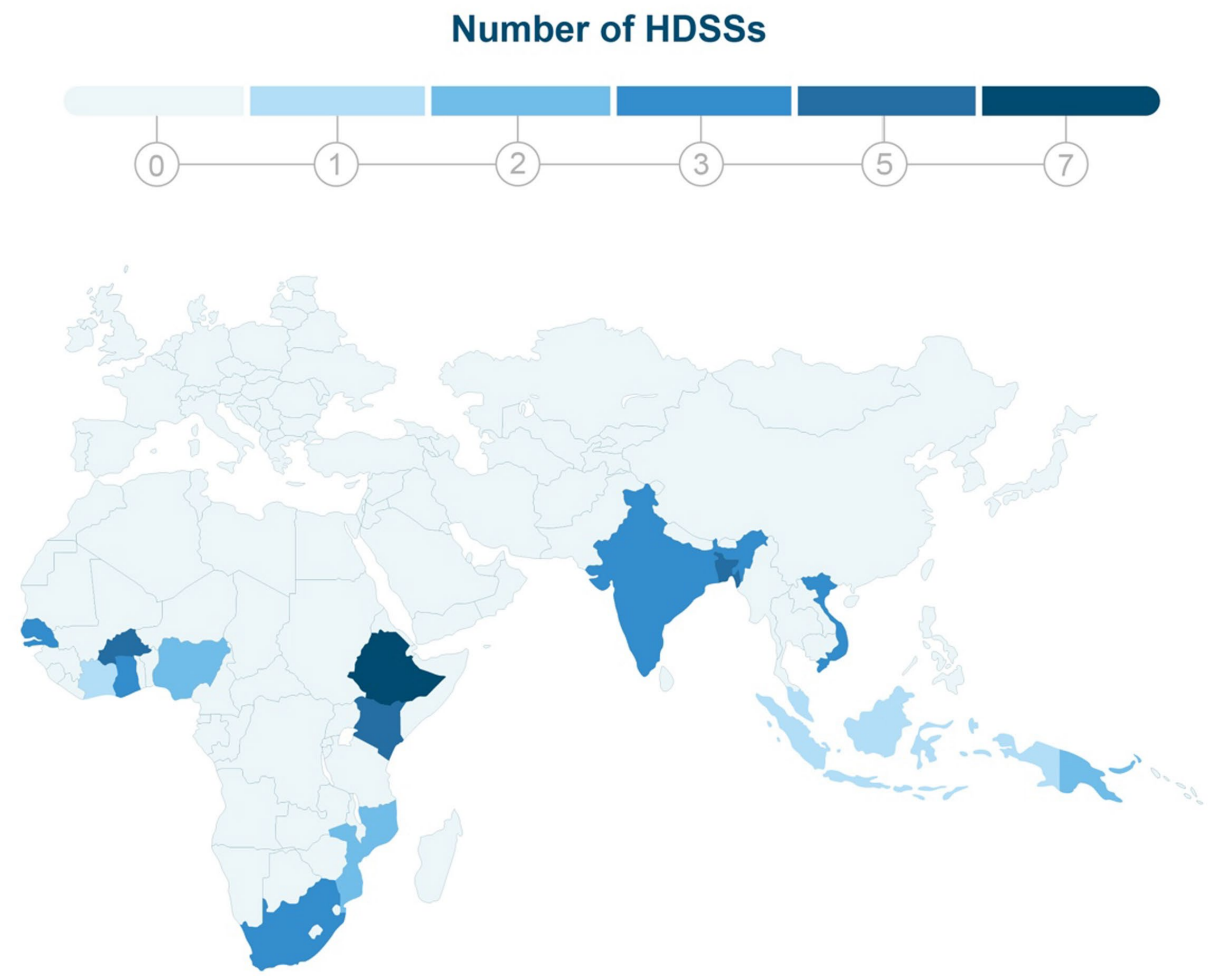
This map depicts the global distribution of 56 Health and Demographic Surveillance Systems (HDSSs) across Asia and Africa. The color-coding scheme used in the map indicates the number of HDSSs in each country, with darker shades of blue representing a higher concentration of HDSSs in that region.

HDSSs are established in 56 low-and middle-income countries (LMICs) across Africa, Asia, and Oceania (see Figure 1). These systems address the scarcity of population-health data and serve as invaluable resources for local and national decision-makers (1, 6, 7). The cohorts account for more than 45 million person-years of observation, spanning up to 60 years, and rely on standardized, quality-controlled research protocols (6–8). The foundation of each health and demographic surveillance system (HDSS) database is established through a baseline census of the respective population. These databases consist of longitudinal records for individuals and social units within designated surveillance areas, which are generated from regular cohort monitoring, typically conducted on an annual basis, and subsequent data collection rounds.

Most HDSSs predominantly gather data on cause-specific mortality outcomes. In contrast, morbidity and social measures are generally addressed only in cross-sectional studies sampled from the entire population (6). Despite the limitation of mortality as the primary endpoint, the longitudinal nature of HDSSs provides an ideal platform for evaluating interventions.

Moreover, HDSSs serve as comprehensive sampling frames for specific populations, which facilitates the collection of samples for targeted surveys and qualitative research. This approach enables a more in-depth understanding of the health and demographic dynamics within the populations under surveillance, ultimately informing more effective public health policies and interventions.

In 1998, HDSSs and other population-based research institutions in Africa and Asia formed the INDEPTH (International Network for the Demographic Evaluation of Populations and Their Health) network to enhance lives, inform health policy, and standardize methods (1, 6, 9, 10). INDEPTH facilitated collaboration among 56 HDSS sites by providing resources, training, and a framework for exchanges, highlighting the benefits of standardization. Each HDSS sustains its funding through various sources, such as national, donor-provided, institutional, and self-acquired funds. Though INDEPTH was active from 1998 to 2015, the network’s cessation has not deterred HDSS sites from operating independently. They continue to form regional networks like the HDSS Asia network (11) and adhere to standardized data collection methods outlined by the INDEPTH website.

The HDSS denominator, which represents the number of persons within the target population, serves as a sampling frame for various epidemiological studies, including cross-sectional surveys, panel surveys, nested cohorts, and randomized-controlled trials (RCTs). With a focus on policy-relevant topics, HDSSs hold considerable potential to inform and influence national and global policies, particularly in relation to the Millennium Development Goals (MDGs) and Sustainable Development Goals (SDGs) (12).

HDSSs have contributed significantly to understanding various health issues, ranging from HIV/AIDS and malaria to adult health, aging, and non-communicable diseases (NCDs) in Africa, South and South-East Asia. These systems also build capacity and strengthen efforts from knowledge generation to improved health policy and practice (13–17). HDSS data repositories house the most comprehensive dataset on cause-specific mortality in LMICs to date. As populations in HDSSs are well-established, they enable observation of changes in population risk factors over time and provide a foundation for determining the effectiveness of community-based interventions (6, 7, 9, 18–20).

### 1.2. Climate change and its impact on health outcomes in Africa and Asia

Climate change significantly impacts global health, imposing a triple burden of disease on vulnerable populations (21, 22). Altered rainfall patterns cause droughts and floods with detrimental health effects (23, 24), while driving spatial and temporal shifts in infectious diseases, child malnutrition, and crop failure, particularly in the Global South (25, 26). Although HDSSs show potential for climate change and health-focused population surveillance, the current literature is sparse, necessitating further investigation. Some previous studies conducted in the Nouna HDSS in Burkina Faso, explored weather changes and child health, focusing on malnutrition (26–30). For example, Diboulo et al. (28) found significant associations between temperature, rainfall, and mortality rates in the Nouna HDSS. Several factors captured in HDSS data collection are directly associated with health impacts related to climate change. These factors include age (31), socioeconomic status (32, 33), occupation (34, 35), and access to resources and infrastructure (36), all of which contribute to a broader understanding of health outcomes linked to climate change. However, limitations exist in current approaches. Many studies rely on satellite or weather station data, which lack granularity for accurate exposure and correlation assessments with health outcomes airports (37). Additionally, the focus on mortality outcomes overlooks climate change’s impact on existing health conditions, highlighting the need for more robust research methods. The existing literature using HDSS data in this context is sparse, highlighting the need for further investigation and insights in this area. A significant portion of climate change and health research relies on weather and climate data obtained from satellites or proximate weather stations, often located at airports (37). Such methods lack the granularity required to accurately capture spatial and temporal variations in weather exposures, which is essential for determining exposure and correlation relationships with population-health outcomes. Furthermore, many studies predominantly focus on mortality outcomes, neglecting the substantial influence of climate change on the progression and severity of existing health conditions, such as cardiovascular disease or asthma.

### 1.3. Importance of collecting morbidity data routinely in HDSS

Life lived with diseases constitutes a critical component of a population’s overall disease burden. The disability-adjusted life years (DALYs) metric is commonly employed to quantify this burden, as it combines years of life lost (YLL) due to premature mortality with years lived with disability (YLD). This information equips policymakers, researchers, and health organizations with the necessary insights to allocate resources, interventions, and research efforts based on the relative impact of different diseases and risk factors.

The ongoing epidemiological transition is marked by a growing prevalence of non-communicable diseases, which frequently lead to diminished quality of life due to chronic, recurring symptoms, rather than causing premature death. Meanwhile, communicable diseases continue to pose a substantial burden in low-resource countries and present the risk of pandemics. Concurrently, the influence of climate-sensitive diseases is escalating globally.

Given these worldwide trends, it is crucial that health and demographic surveillance systems (HDSSs) consistently capture data on morbidity alongside mortality, which is currently an infrequent practice (38). Solely relying on mortality to assess the effectiveness of health interventions and policies is inadequate, as it fails to account for the full scope of disease impacts.

By systematically collecting data on YLD, YLL, and climate-sensitive variables, it becomes possible to calculate climate DALYs (cDALYs) that estimate the health impacts of climate change. Additionally, it is important to recognize that seasonal patterns play a role in the occurrence of various diseases (39), including climate-sensitive ones, as well as in fluctuations of household income, expenses, and assets (40). Incorporating these metrics into HDSS data collection fosters a more comprehensive understanding of a population’s disease burden. This holistic approach ultimately facilitates better-informed decision-making concerning resource allocation, intervention strategies, and research priorities in public health.

### 1.4. Climate and health surveillance and response system (CHEERS)

The CHEERS system incorporates components, such as individual exposure sensors, indoor/outdoor temperature and humidity measurements, remote sensing for land use/cover data, population-based disease assessments, and innovative data storage, that seamlessly integrate with existing research infrastructures like HDSS. This cohesive framework enables a rigorous examination of climate change and health interrelations, providing crucial insights for researchers and policymakers.

## 2. Methods

### 2.1. Context of CHEERS within the Nouna HDSS, Burkina Faso

The CHEERS system, integrated with the Nouna HDSS in northwestern Burkina Faso (7), collects climate change and health data. Managed by the Centre de Recherche en Santé de Nouna (CRSN),^1^ the Nouna HDSS has over 31 years of health and population data, surveilling 125,000 individuals by the end of 2019, resulting in over 2.5 million person-years of observation (7).

The Nouna HDSS covers a subset of the Nouna Health District, encompassing approximately 1,755 km^2^. Comprehensive population censuses were conducted in 1992, 2005, 2009, and 2019. In 2007, the town of Nouna represented about 30% of the HDSS population with roughly 33,844 inhabitants. Initiated in 1992, the Nouna HDSS initially incorporated 39 villages within three CSPS (Centre de Santé et de Promotion Sociale; primary health care facilities in francophone West Africa), totaling 26,626 people. The HDSS expanded to include Nouna town and two villages in 2000, followed by an additional 17 villages in 2004, resulting in a total of 58 villages (59 including Nouna). By 2009, the area had one hospital and 13 out of 29 CSPS for the entire district. However, the majority of deaths are still reported at home, with only about 30% of deaths occurring in health facilities (see text footnote 1). The HDSS represents roughly a quarter of the Nouna Health District in terms of area and 1/3 in terms of population. Although the Nouna HDSS population is not randomly selected, key variables are consistent with national-level observations, allowing for cautious generalization of results from the Nouna area.

The monitoring area of the Nouna HDSS features a tropical climate with a single rainy season extending from June to October, accompanied by an annual average rainfall of 800 mm (ranging from approximately 480 – 1,085 mm) and consistently high temperatures throughout the year. The region experiences a high prevalence of malnutrition and malaria (24). The consequences of climate change on public health are anticipated to disproportionately affect subsistence farmers and economically disadvantaged individuals due to their limited resources for implementing adaptive and mitigative measures.

### 2.2. Climate change and health-related variables

In 2020, five weather stations were installed across the Nouna CHEERS site to represent various agro-ecological zones. A sensor-based sub-cohort, stratified by age and gender, collected data on daily activity, sleep, heart rate, indoor temperature, and humidity [refer to (41, 42)]. Remote sensing approaches, including agricultural yield models, were employed to estimate crop productivity (43, 44). The morbidity component, covering 10% of the Nouna HDSS population, began in May 2022, initiating comprehensive health data collection.

**Figure 2:**
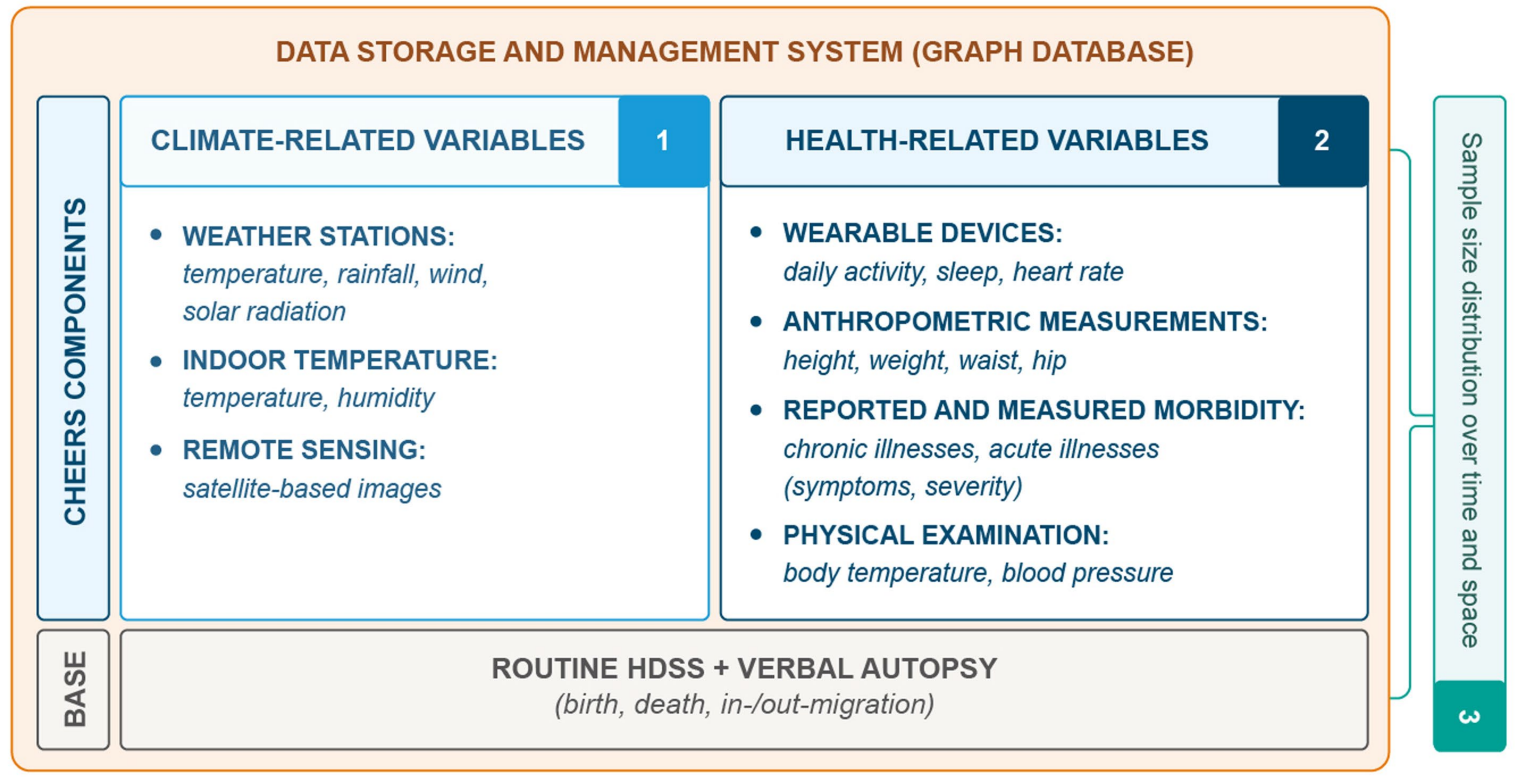
Overview of the CHEERS site data structure, which includes the routine HDSS data collection and the novel CHEERS components of climate-related data (1), reported and measured health status (2), as well as a novel sample size distribution over time and space (3) to optimize resources needed for data collection. The collected data of the CHEERS is managed with a graph-database which provides a flexible data storage and management system.

**Figure 3:**
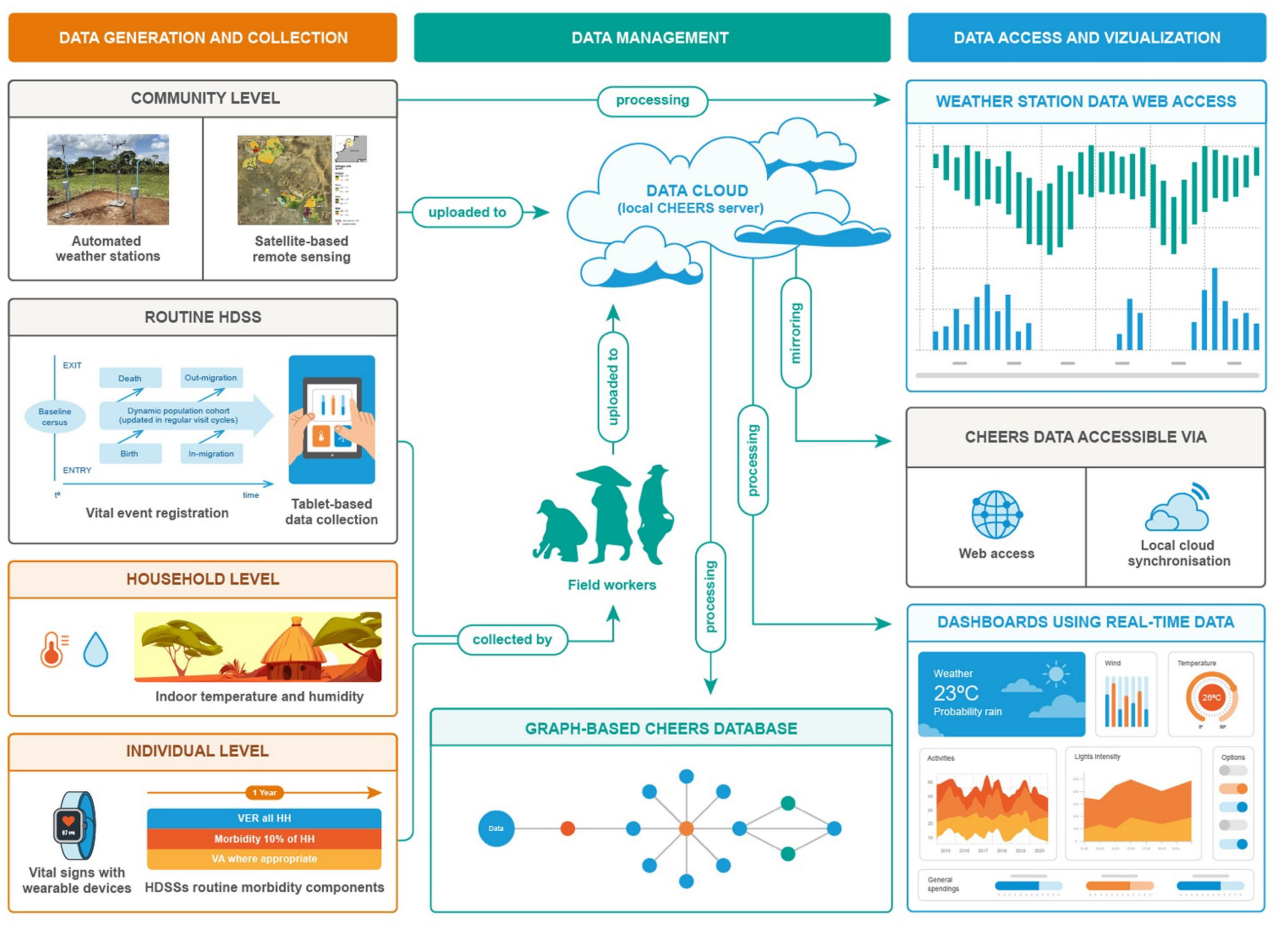
CHEERS data collection system comprising data collected at the individual level (wearable devices; routinely collected morbidity data), the household level (indoor temperature of houses), the community level (automated weather stations; satellite-based remote sensing including land use classification (LUC), land cover classification (LCC), surface water and wetlands) and the routine HDSS data collection (vital events registration).

Device selection was informed by literature reviews (37, 45) and feasibility assessments (41, 42, 44). Table 1 summarizes the collected data, while Figure 2 illustrates the CHEERS structure, including routine HDSS data, climate-related data, reported and measured health status, and an optimized sample size distribution. A graph database manages the data, providing flexible storage and management. Figure 3 outlines the CHEERS process from data generation to visualization.

**Table 1:** Overview of components and climate change-related health variables collected in as part of CHEERS within the Nouna HDSS, Burkina Faso.

Type of data	Measured variables	Employed devices
Climate-related data	Indoor (in every sampled household, *n* = 500): temperature and relative humidity measurements (every 15 min)	iButton data logger; Switchbot
	Outdoor (5 weathers stations spatially covering the Nouna HDSS catchment area in 10 km diameter): temperature, precipitation, wind speed, wind direction, solar radiation (every 15 min)	Validated weather stations
	Remote sensing-based land use and land cover classifications, surface water occurrence maps (quantification and identification of water bodies and wetlands)	Sentinel-1 and Sentinel-2 satellite
Reported and measured health status	Consumer-grade wearable devices (daily activity measured in steps, sleep with regards to sleep length and sleep quality, heart rate; in a sampled population of *n* = 500)	Garmin vivosmart 5 (former: Withings Pulse HR)
	Anthropometric measurements (in 10% sub-sample of HDSS population)	Recumbent length: Seca 417 (measuring range 10–100 cm); used for children unable to stand or shorter than 85 cm Height: Seca 213 (measuring range 20–205 cm); used for children able to stand and taller than 85 cm Weight: Seca 878 (measuring range up to 200 kg, uncalibrated) (WHO Child Growth Standards, 2006; World Health Organization (WHO), 2008)
	Morbidity-relevant questions incorporated into the standard HDSS data collection, focusing on self-reported health status (in 10% sub-sample of the HDSS population)	Android-based tablets to run Survey Solutions software

Data is collected at community, household, and individual levels, with weather stations and satellite-based remote sensing providing community-level data. Routine HDSS data is collected via tablet-based systems, household-level data includes indoor temperature and humidity measurements, and individual-level data involves vital signs measured with wearable devices and self-reported morbidity. Field workers transmit collected data to a server, where raw data is processed and loaded into a graph database, enabling direct access through dashboards. Researchers access data via internal cloud services like Seafile.

#### 2.2.1. Climate-related variables

The climate-related data collected as part of CHEERS (see Figure 2, denoted by number 1) includes continuous measurements from weather stations, providing information on precipitation, temperature, wind (speed and direction), relative humidity and solar radiation. Additionally, indoor devices containing a thermo-and a hygrometer measure temperature and humidity, while remote sensing approaches, such as the use of Sentinel-2 imagery, contribute insights into land use and waterbody characteristics.

##### 2.2.1.1. Indoor temperature of houses

**Figure 4:**
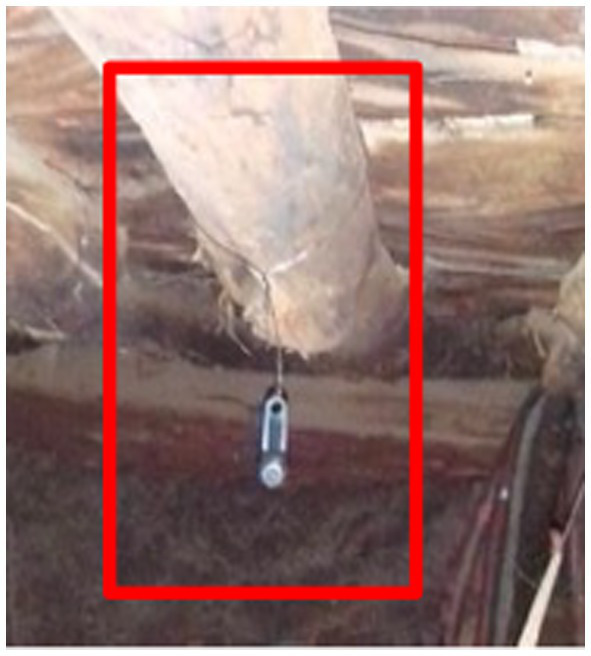
Installed sensor that measures indoor temperature and humidity, which is part of the household-level data collection in CHEERS.

Indoor air temperature and relative humidity are systematically monitored using a single datalogger per household, installed in the primary living room or bedroom areas (see Figure 4). These dataloggers are mounted 1.5–2 meters above the ground on interior walls, above adult head height, to minimize contact. They are strategically positioned away from direct sunlight and heat sources (e.g., cooking areas) while maintaining exposure to the main body of the room’s air (avoiding concealment behind curtains or placement in isolated corners).

Weather parameters are recorded every 15 min to capture the majority of daily fluctuations. At the Nouna CHEERS site, indoor temperature measurements are collected for *n* = 500 households, where sampled household members also wear consumer-grade wearable devices. Data synchronization occurs every 4 weeks using a portable laptop and USB adapter for iButton devices, and tablet and Bluetooth for the Switchbot Meter. iButton devices were chosen for temperature and humidity logging due to their established and validated performance in scientific literature, resilience in dusty and extreme environments, and long battery life of at least 1 year. Additionally, we are currently testing SwitchBot meters, which are more cost-effective and feature a display for temperature and humidity measurements, potentially providing study participants with a better understanding of their exposures.

##### 2.2.1.2. Remotely-sensed data

Remote sensing techniques utilize satellite-derived geospatial data to analyze the effects of climate change on various health and environmental factors. For instance, these methods can examine the impact of climate change on childhood macro-and micronutrient deficiencies, as well as its influence on crop yields and food production (29, 30, 44). By constructing spatio-temporal models, remote sensing can also assess the association between climate change and malaria prevalence. Moreover, these techniques allow for the refinement of local climate variables and the quantification of uncertainties within the climate-malaria modeling framework. Data is freely accessible through the Copernicus Open Access Hub and the Copernicus Data Space Ecosystem^2^.

For the satellite-based annual yield models at the household field level, we collected field data for five primary food crops (maize, millet, sorghum, beans, and sesame) over 3 years (2018, 2020, and 2021) (43). Seven agricultural surveyors were trained to use GPS devices for field boundary sampling (*n* = 1,027) and to install, monitor, and harvest yield squares (*n* = 411). Each field was assigned a unique identifier to link it to individual households. The selection of fields aimed to maximize variation to represent the full range of variability in the study area.

Sentinel-2 Level-1C images, featuring 10 m resolution and a 5-day revisiting cycle, were employed for the crop growing seasons of 2018, 2020, and 2021. These images underwent preprocessing, including atmospheric correction using Sen2Cor version 2.10. Subsequently, monthly maximum NDVI (normalized difference vegetation index; assessing vegetation greenness or photosynthetic activity) composites were generated to minimize cloud influence by applying the NDVI formula [NDVI = (NIR – Red) / (NIR + Red)], where NIR denotes the near-infrared band and Red denotes the red band. Monthly NDVI composites were created by selecting the maximum NDVI value from all available NDVI images within a month for each pixel location.

For each composite, three vegetation indices—NDVI, NDRE (normalized difference red edge; evaluating plant health and stress, especially in crops), and NDWI (normalized difference water index; measuring vegetation water content and detecting land surface water feature changes)—were calculated. These indices effectively monitored vegetation and estimated yields. In total, 10 monthly composites were computed for each vegetation index from March to December for each year, with each monthly composite acting as a predictor for the linear regression model. Pixel values at the 5 × 5 m sampled harvest squares were extracted for each agricultural field.

Daily CHIRPS 2.0 global dataset provided rainfall data for the model, summarized weekly from March to October (46). Monthly composites and weekly rainfall datasets served as model predictors using LASSO regression (43).

#### 2.2.2. Automated weather stations

**Figure 5:**
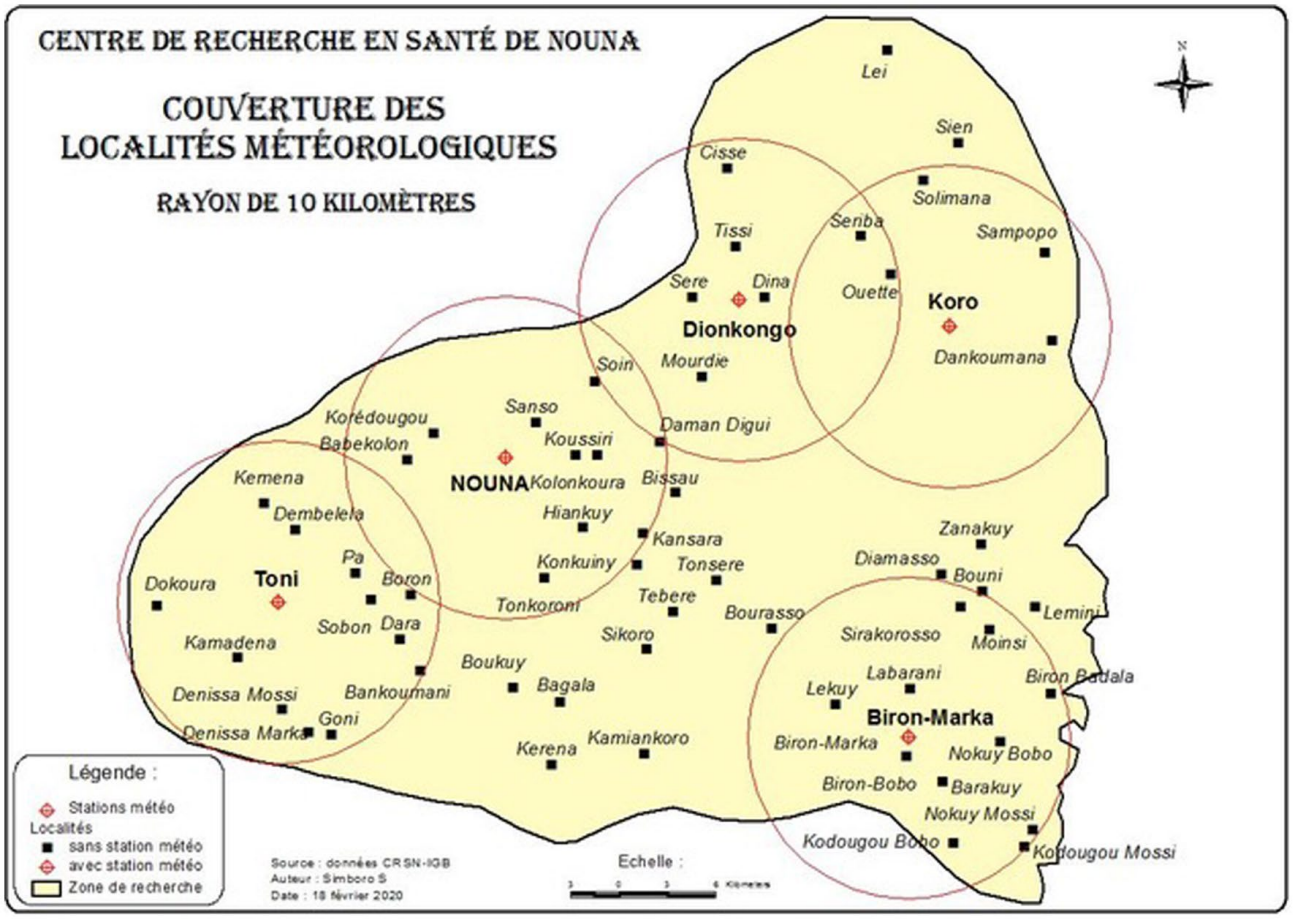
Map of Nouna CHEERS area in Burkina Faso indicating the distribution of five weather stations installed in March 2020. The weather stations provide comprehensive meteorological data for the region, measuring precipitation, temperature, solar radiation, wind speed and direction, and relative humidity (station locations encircled).

The Nouna CHEERS site has strategically deployed five automated weather stations to maximize spatial coverage of different agro-ecological zones. These stations continuously transmit data through General Packet Radio Service (GPRS), enabling real-time access to weather information on a locally hosted web platform (see Figure 5). Weather stations can run autonomously (solar-powered) and synchronize data automatically every 15 min via mobile data. The weather stations collect six meteorological variables: air temperature, precipitation, solar radiation, relative humidity, wind speed, and wind direction. Site selection was contingent upon negotiations with landowners for land usage permissions. Additionally, community engagement and sensitization activities were conducted to educate the local population about the purpose and importance of weather stations, as well as their role in generating valuable data for climate change and health research.

### 2.3. Health-related variables

The CHEERS system enables the collection of various health-related variables at the individual level, including sensor-based data such as daily activity, sleep quality and duration, and heart rate, as well as anthropometric measurements like height, weight, waist, and hip circumference (see Figure 2). Additionally, the study incorporates both reported and measured morbidity data. Reported morbidity is obtained through supplemental questions posed to the HDSS study population, eliciting further details about symptoms and severity of acute and chronic illnesses (see Supplementary Appendix 2 for CHEERS questionnaire).

#### 2.3.1. Continuous individual vital sign collection: consumer-grade wearable devices

In the Nouna CHEERS site, we have employed consumer-grade wearables to generate high-resolution individual-level data (41), enabling ecological momentary assessments (47) of health parameters within real-life environments. The feasibility and acceptability of these wearable devices have been assessed in the Nouna HDSS (41), providing valuable insights into individual activity profiles, sleep patterns, and vital signs such as heart rate (42). These variables, such as activity profiles, sleep patterns, and vital signs like heart rate, are typically measurable by commercial wearable devices, and offer valuable individual health insights. At the Nouna CHEERS site, study participants wear a consumer-grade wearable device, currently a Garmin vivosmart 5 (refer to Table 2 for details), that continuously records data 24 h a day. The device is worn on the wrist and can store up to 14 days of data with a 7-day battery life. We previously used the Withings Pulse HR, but it was not found to be well-suited for collecting population-health data in rural Burkina Faso (42). The data from the wearable device is synchronized with a field worker’s tablet via Bluetooth, and the field worker visits the participant’s home every 5–7 days. To accommodate remote areas without access to an electrical grid, participants are supplied with battery packs and foldable solar panels for charging wearable devices.

**Table 2:** Overview of consumer-grade wearable devices employed in the Nouna CHEERS, Burkina Faso, and in the Siaya HDSS, Kenya.

	Garmin vivosmart 5
Features	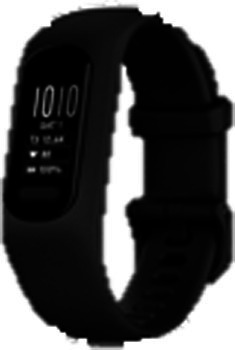
Steps measurements	Measured continuously, steps identified based on amplitude and periodic pattern Technology: accelerometer
Heart rate measurements	Routinely measured every 10 min Measurement frequency every 1 s (continuous heart rate mode) only in workout session or after 2 min of running Technology: photoplethysmography
Sleep measurements	Four variables calculated: total hours of sleep, sleep stages, sleep movement, and sleep score
Pulse oximeter measurements	Measures blood oxygen level (SpO_2_) by shining light into the skin and checking how much light is absorbed.
Wear location	Wrist
Data synchronization	Up to 1 month activity tracking, up to 3 weeks activity tracking plus extensive fitness activity use
Battery life	Up to 7 days
In-built sensor	High precision MEMS 3-axis accelerometer Photoplethysmography sensor
Connectivity	Bluetooth low energy

#### 2.3.2. Self-reported morbidity

The updated HDSS questionnaire (refer to Supplementary Appendix 2 for full CHESS questionnaire) incorporates a comprehensive morbidity component consisting of 57 questions that covers both acute and chronic illnesses, as well as symptoms, which were validated against a well-established list. This component is routinely administered to a 10% sample of households within the CHEERS site, totaling at least 1,500 households. The questionnaire captures the date of illness onset (day, month, year), associated disability, and illness severity. To complement the self-reported data, anthropometric measurements are taken such as weight, height, waist circumference, and hip circumference.

### 2.4. Sampling

In 2019, the latest HDSS census was conducted, registering a total of 124,957 individuals residing in 15,014 households across 58 villages and the town of Nouna, comprising of seven sectors.

#### 2.4.1. Climate-related variables

##### 2.4.1.1. Indoor temperature

At present, the indoor temperature surveillance in the CHEERS system is limited to a sample of 500 households due to funding and data collection constraints. However, with additional funding, it is envisaged that a larger sample of at least 1,500 households (10% of the total CHEERS population) can be achieved. To facilitate participant recruitment, CRSN field workers collaborated with community leaders and household heads to inform them about the data collection procedures and the importance of the study.

##### 2.4.1.2. Remotely-sensed data

Sentinel-2 images, featuring a 10-meter spatial resolution, are supplied by the European Space Agency (ESA) every 5 days and can be freely accessed via the Copernicus Open Access Hub. To develop satellite-based multi-annual yield models for major food crops, a minimum sample size of *n* ≥ 25 yield squares per year is targeted for each crop type, ensuring the model’s robustness and reliability (refer to (43) for more information).

##### 2.4.1.3. Weather stations

The optimal number of weather stations required to accurately cover a specific area is dependent on various factors, such as spatial variability in climate, topography, and land use. To determine the number of stations required, we installed five weather stations in the Nouna CHESS site to assess spatial variability in climate parameters and refine our sample size estimation. Statistical methods such as geostatistical interpolation techniques (e.g., kriging) (48, 49) and the variogram method (50) are used to estimate the optimal number of weather stations needed based on the level of accuracy desired and the results from the initial set of weather stations. We also consider practical constraints such as accessibility, maintenance requirements, and costs when selecting weather station locations to balance comprehensive coverage of the study area with resource management (see Figure 5).

#### 2.4.2. Health-related variables

##### 2.4.2.1. Wearables

The sample sizes in our study are stratified by age and gender. Participants under the age of 18 years have been excluded from the sensor-based cohorts as wearable devices were deemed unsuitable for them. This is because commercial wearables are generally designed for adults and may not accurately capture data for younger individuals due to differences in physiology and activity patterns. However, we may consider including younger participants in future studies if appropriate wearable devices become available. At present, the monitored sub-population consists of 500 study participants, primarily due to financial constraints related to the cost of wearable devices and data collection expenses. It is envisaged that a larger sample of at least 1,500 households (10% of the total CHEERS population) can be achieved in the future.

##### 2.4.2.2. Vital event registration (VER) and self-reported morbidity

The VER and reported morbidity data collection is grounded in the latest 2019 census data, which documented 124,957 individuals distributed across 15,014 households in 58 villages and the town of Nouna. The questionnaire (refer to Supplementary Appendix 2) is administered to all 15,014 households, with 10% of these households (or at least 1,500) being selected for morbidity monitoring. Employing stratified random sampling proportional to each stratum’s size, the Nouna CHEERS is organized into 52 clusters, with each cluster comprising approximately 289 households (52 × 289 ≈ 15,014 households). In every cluster, around 29 households are monitored for morbidity (52 × 29 ≈ 1,500).

Over a 52-week period, Nouna CHEERS collects morbidity data from about 29 households in each of the 52 clusters. This translates to field workers visiting approximately 29 households per week for morbidity monitoring in addition to vital event registration. For the vital event registration, data is collected from roughly 289 households per week, spanning across the 52 clusters. To minimize seasonal bias, the 52 clusters are allocated one cluster per week.

#### 2.4.3. Data collection

##### 2.4.3.1. Climate-related variables

###### 2.4.3.1.1. Indoor temperature

Each month, field workers synchronize both wearable data and indoor temperature measurements with laptops or tablets using Bluetooth technology at the Nouna CHEERS site. The indoor temperature devices used in the study include the iButton, which can log data for up to 1 year, and the Switchbot meter, which can log data for up to 36 days. These durations are long enough to allow for monthly synchronization. After synchronization, the data is transferred to the CRSN server for further processing and analysis.

###### 2.4.3.1.2. Remotely-sensed data

The Copernicus Open Access Hub offers free access to Sentinel-2 images with a 10-meter spatial resolution, available at Level-1C every 5 days. These images can be further processed, including atmospheric correction using Sen2Cor version 2.10. Remote sensing data also allows for more in-depth analysis of land cover and water bodies, utilizing Sentinel-1 radar data for the latter. In the development of satellite-based multi-annual yield models for major food crops, a target sample size of *n* ≥ 25 yield squares per year was set for each crop type.

###### 2.4.3.1.3. Weather stations

The meteorological variables collected by the weather station are automatically recorded in the remote terminal unit (RTU), which is installed on the station. The weather station runs autonomously, powered by a small solar panel and battery, ensuring self-sufficiency. Field workers who have received training in weather station management perform routine checks on the weather stations on a monthly basis. These checks include activities such as mowing grass, inspecting the station for insect nests and damage, and ensuring that the station is functioning properly. In addition, the web platform is equipped with automatic notification triggers that alert the weather station managers in the event of any interruptions, such as issues with mobile data transmission or electricity supply from the solar panel.

##### 2.4.3.2. Health-related variables

###### 2.4.3.2.1. Wearables

Weekly, field workers visit households of study participants to collect data from the wearable devices they wear. In rural areas, offline synchronization features are used to synchronize the data with tablets carried by the field workers. The collected data is then transmitted to the CRSN server once the field workers return to the headquarters and have mobile data connection. This data collection process is separate from the routine CHEERS data collection, as it involves a different data collection frequency compared to the collection of VER and morbidity data.

###### 2.4.3.2.2. Vital event registration and reported morbidity

Data collection at the Nouna CHEERS site utilizes electronic devices like tablets and smartphones, with field interviewers transmitting data to the CRSN server via the internet. The process involves field staff training, field deployment, data monitoring, and multi-level validation. Field staff receive training on electronic data collection devices and safety guidelines. Quality assurance is maintained through a hierarchy of supervisors and central controllers who review, correct, and approve data.

**Figure 6:**
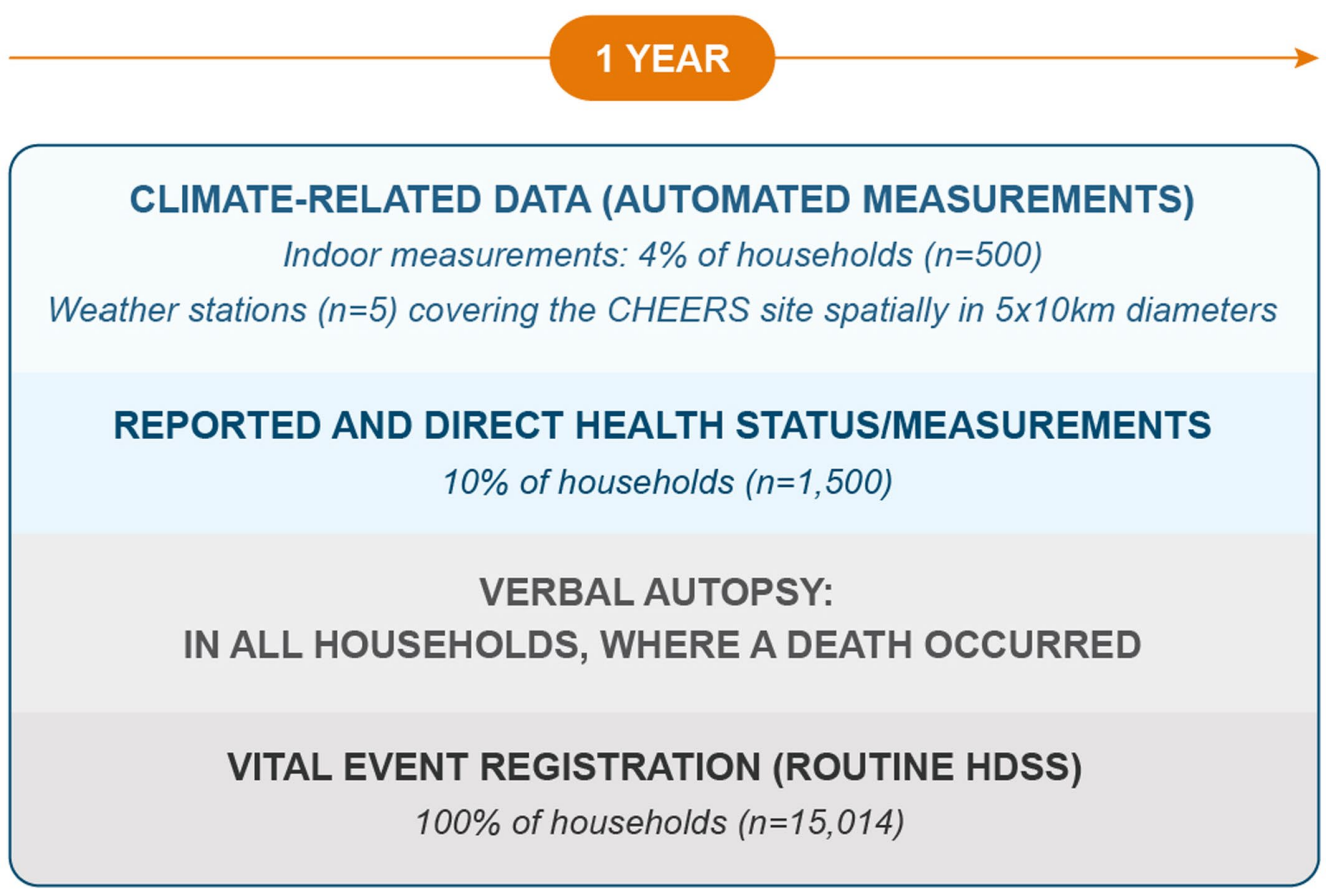
The CHEERS framework creates an integrated routine data collection system that encompasses several key modules: vital event registration (collected for all households within the CHEERS site), verbal autopsy (conducted in households reporting a death, with interviews carried out by a proxy household member), and reported morbidity data (including information on chronic and acute diseases, collected for 10% of households, up to a maximum of 1,500 households). Climate-related data is currently obtained from 500 households (indoor temperature) and 500 individuals (wearable devices measuring daily activity, sleep, and heart rate) in the Nouna CHEERS site. Presently, five weather stations are deployed in the Nouna CHEERS site, with ongoing evaluation to determine their sufficiency in covering the area.

CHEERS data collection combines vital event registration (see Figure 6), morbidity assessment, and verbal autopsy (where applicable) in a single visit, adopting a proxy+ approach to gather information about household members. This one-stop data collection reduces budgetary burden associated with staffing, fuel, and other consumables. Nouna CHEERS currently employs 20 permanent field workers, each responsible for one stratum.

### 2.5. Flexible data storage and management systems: Graph database

**Figure 7:**
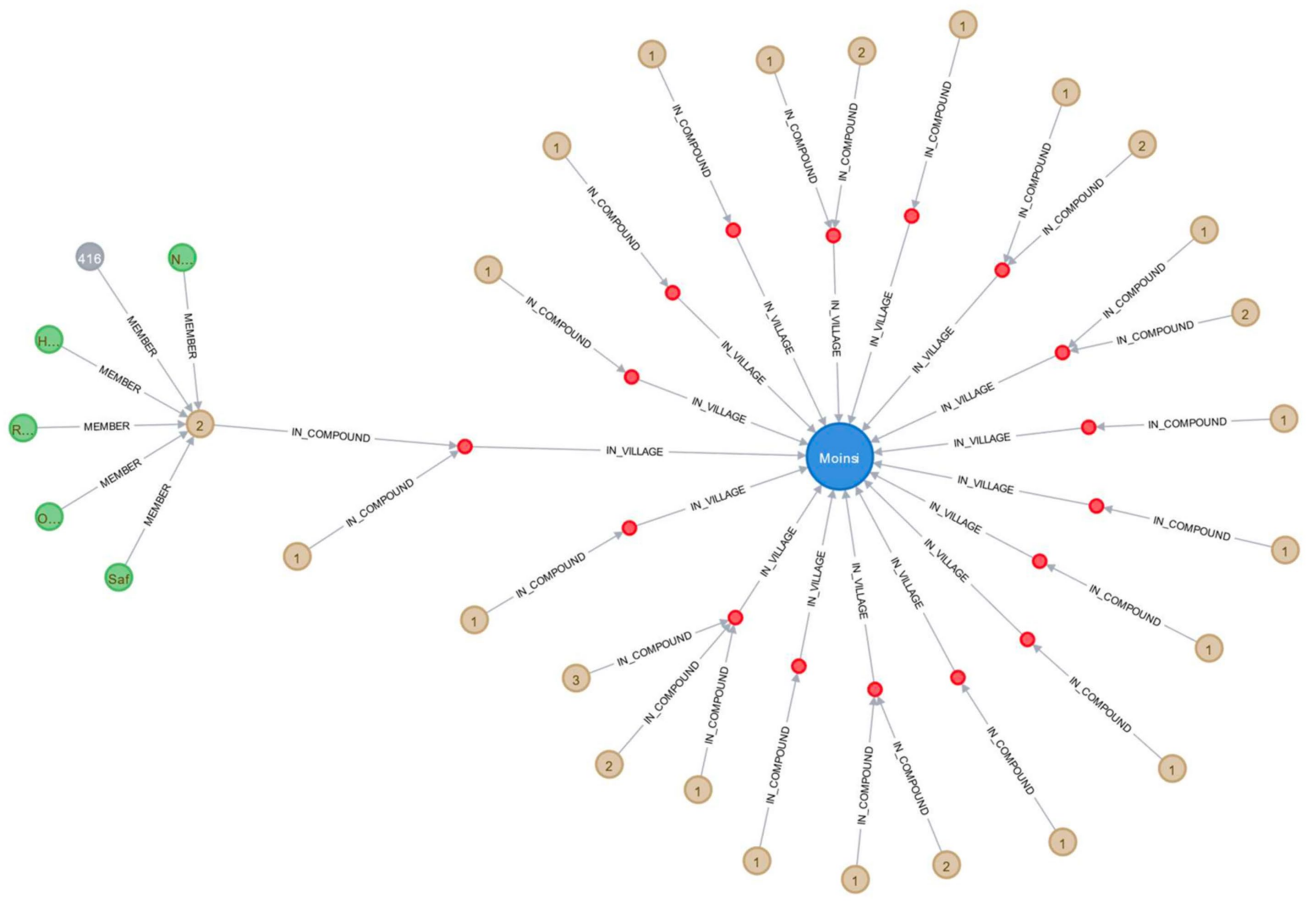
Visual representation of health and demographic data in a graph database for the Nouna HDSS, showcasing the village of Moinsi, its compounds, households, and current household members.

Relational databases, which organize data into tables and establish connections between them, have been the standard method for managing large datasets. However, they can be inflexible when integrating new data sources, particularly those with varying granularities and structures, such as remotely sensed satellite images, individual-level wearable data, or meteorological data from weather stations. To address this issue, we explored the use of a graph database to store and manage data in the Nouna CHEERS system. In a proof-of-concept study, we migrated the 2019 census data from the Nouna HDSS into a graph database and subsequently combined the entire HDSS database from 1992 to 2019. Unlike relational databases, which use tables, the graph database is a mathematical graph consisting of nodes and edges that connect some of the nodes. This structure allows for the representation of networks within the population, such as compounds, households, individuals, and assets, which can be connected by various relationships. The graph database can also store all relevant health and demographic data points, including geospatial properties, dates, household data, individual data, and compound data (see Figure 7).

CHEERS graph database comprises:

- **Nodes**: Object types stored in nodes are described by labels. Many different labels can be assigned to a single node. Certain aspects of a node can be encoded using so-called properties, which are key-value pairs.
- **Relationships**: Relationships have unique labels that indicate the type of relationship they represent; relationships can also have features, such as a quantity, date, etc., that further define the relationship.
- **Properties**: Nodes and relationships may also have properties. They store the values of certain interview-gathered variables or values derived from those variables.

The CHEERS system synchronizes data automatically to the cloud or manually by field workers, allowing for the development of dashboards that offer visual representations of climate change and health impacts in vulnerable populations. Pilot dashboards have been designed to directly feed data from wearables and weather stations, with the aim of providing information to guide decision-making. Usability evaluations and user knowledge and emotional impact assessments are currently ongoing. The dashboard aims to make data easily accessible, provide knowledge on climate change’s impact on vulnerable populations’ health, and deliver key information to decision-makers, policymakers, and the public. Future additions will include remote sensing, morbidity, and weather station data.

### 2.6. Ethical considerations

Ethical approval for this research has been granted by the Heidelberg University Hospital Ethics Committee, the Comité d’éthique pour la Recherche en Santé of the Ministère de la Santé Burkina Faso, and the Kenya Medical Research Institute (KEMRI) Scientific and Ethics Review Unit (SERU). Informed consent procedures are managed by the respective institutions leading the Health and Demographic Surveillance System (HDSS). Informed consent templates are available on the INDEPTH website,^3^ which also provides further information on privacy and confidentiality measures.

To ensure data security and integrity, stringent regulations are in place to control access to various data types and the timing of access. Continuous data entry necessitates daily backups across multiple media, with a redundant hard drive configuration recommended to maintain data backups. In accordance with INDEPTH guidelines, regular database backups are performed, and offsite storage is utilized for these backups, separate from the main office.

In addressing the use and protection of personally identifiable data (PID), we employ a stringent anonymization process in which respondent names are replaced with unique identification numbers throughout data analysis and presentation. This approach ensures that the collected data cannot be associated with specific individuals, thereby preserving participant anonymity.

Access to the data is rigorously controlled, with only authorized researchers who have completed relevant training in data security and privacy protection granted permission. The sharing of data with external parties is contingent upon a thorough review and approval process, which ensures adherence to the same rigorous privacy and confidentiality standards across all parties involved.

Each HDSS, and subsequently the CHEERS framework, determines the type and amount of compensation provided to participants. For further details on this matter, please refer to the HDSS profiles (7). By implementing these measures, we aim to protect the privacy of the participants and maintain the highest ethical standards in our research.

## 3. Results

The Nouna CHEERS project was initiated in 2022, and data collection is currently ongoing, as described in the methodology sections. All components of the CHEERS system outlined in the methodology section have been implemented. Our study results will be disseminated through future publications. Additionally, initial components of the CHEERS system have been implemented in the Siaya HDSS in Kenya [for further details, see (41)] and the SEACO HDSS in Malaysia (51). While findings from all components are still pending publication, some aspects of the CHEERS system have already been reported in published studies, which are summarized below.

### 3.1. Climate-related data

#### 3.1.1. Remotely-sensed data

In a pilot study (44) and a subsequent case study in the Nouna HDSS (43), we developed a model for predicting yield at the field level (individual fields of households of study participants in the Nouna CHEERS site). Using time series of Sentinel-2 satellite images (10 m spatial resolution), we calculated monthly vegetation indices, including NDVI, NDRE, and NDWI, to monitor crop growth and estimate yield. Yield models were developed for each crop type and a multi-annual yield model was established, incorporating a total of 65 variables. These variables included 30 for vegetation indices – 10 for each index – and 35 for precipitation, representing 35 weeks of accumulated rainfall. LASSO regression with 5-fold cross-validation was utilized for model development. The models capture a portion of the inter-year variability in yields, enabling prediction of yield estimates at the household level without new *in situ* measurements. This linkage facilitates the assessment of the relationship between household-level yields, socioeconomic indicators, nutritional status of children, and the health status of household members. See (43, 44) for more information.

### 3.2. Self-reported and measured health status

#### 3.2.1. Continuous individual vital sign collection: consumer-grade wearable devices

We conducted an observational study from January 2021 to March 2021 to investigate the feasibility and acceptability of using wearables to generate individual-level data in rural Burkina Faso (42). The study involved 148 participants wearing a wearable (Withings Pulse HR wristband tracker) and a thermometer patch (Tucky axillary thermometer patch) for 3 weeks. We found that wearables can generate large and longitudinal datasets on activity, sleep, and heart rate, which can complement existing population health routine measurements such as in the HDSSs (42). The study highlights the potential of wearables to generate objective insights into individual activity and vital patterns in low-resource settings. Accelerometry data were generated most reliably, while photoplethysmography and thermometer measurements proved more difficult with higher data missingness. However, the acceptability did not appear to affect the data quantity and quality. The study underscores the importance of open communication and regular follow-ups of study participants to avoid distress and improper use of the wearables. The study findings indicate that the use of wearable technology can be a valuable tool in population health surveillance in low-and middle-income countries (LMICs). However, it is important to acknowledge and address the technical challenges and barriers associated with the implementation of such data collection modules (42).

We carried out an observational study spanning from August 2021 to June 2022, collecting data for 11 months, to investigate the effect of extreme weather on the health of the rural population in Burkina Faso using wearable devices (52). Linear mixed effects models were used to estimate the relation between heat and precipitation with daily activity, sleep duration and heart rate. The study found that sleep duration decreased significantly with higher heat exposure, while daily activity was highest during rainy season when WBGT was highest, but decreased when daily maximum WBGT reached 30°C, which is considered dangerous heat exposure. HR data had insufficient data completeness during daytime and nighttime HR showed no statistically significant correlation with heat exposure. Heavy rains did not impair health parameters measured in this study. The study concludes that heat especially impaired sleep and daily activity of the participants, highlighting the need for research on appropriate interventions and adaptations to reduce the adverse impact of weather exposure on their health (52).

## 4. Discussion

The CHEERS (Climate, Health, and Equity: Evidence-based Responses for Sustainability) framework is a novel and modular system designed to broaden the scope of existing health and demographic surveillance systems (HDSSs) and similar research infrastructures to include research on the topic of climate change and its impact on health. By integrating digital innovations at the individual, household, and community levels with the routine collection of vital event data, CHEERS has the potential to generate a wealth of data that can be used to identify the most pressing climate change and health-related priorities, particularly for vulnerable populations. The framework enables the long-term monitoring of population health status and the health effects of environmental exposures, providing evidence-based data to support the allocation of resources toward mitigating and adapting to these priorities. The use of digital technologies in data collection also allows for real-time monitoring of population health status and the health effects of exposures, which can be translated into accessible dashboards for policymakers, stakeholders, and the general public for data-driven decision making. Evaluation of individual CHEERS components, such as wearables (42, 52), and remote sensing (43, 44), and, has shown promising results, with ongoing efforts to integrate CHEERS and health district data for a comprehensive population health monitoring system. Ultimately, CHEERS has the potential to transform HDSSs and similar research infrastructures into climate-and health-ready research infrastructures, allowing for the generation of data that can inform public health policy and practice.

### 4.1. Self-reported and measured health status

#### 4.1.1. Wearable devices

Wearable devices are increasingly employed in population health research due to their ability to collect large, objective datasets on various health-related parameters, such as activity, sleep, and heart rate. These insights into individual health status are particularly valuable in low-resource contexts where data is often limited or nonexistent. By serving as a crucial tool in population health research, wearables help researchers better understand and address health needs in vulnerable populations.

Correlating wearable data with weather exposure data enables researchers to gain deeper insights into the impact of climate change on human health. Koch et al. (37) demonstrated the effectiveness of wearables in measuring the effects of heat on sleep, physical activity, heart rate, and other physiological responses during wildfires. Combining wearable data with environmental factors allows for prediction tasks, such as disease prediction and estimating working capacity under climate change-induced weather exposures.

Wearable devices have numerous potential health applications, including monitoring diseases like Alzheimer’s, diabetes mellitus, and atrial fibrillation, tracking fertility, and studying associations between lifestyle factors, such as coffee consumption, sleep, physical activity, and blood pressure (45). As wearable technology continues to advance, it holds great promise for generating reliable population health data that can inform research, interventions, and policymaking, particularly in the context of climate change’s adverse health effects.

Cuffless wristband wearables offer continuous, non-invasive blood pressure monitoring in real-world settings, providing crucial insights into individual exposures and health outcomes (53). These devices are particularly significant for individuals with non-communicable diseases, such as hypertension, who may face heightened health risks due to environmental factors like heat. A comprehensive meta-analysis by Liu et al. (54) revealed a correlation between a 1°C temperature increase and a 2.1% rise in cardiovascular disease-related mortality, as well as a 0.5% increase in morbidity.

Another wearable, Kenzen, non-invasively measures core body temperature and sweat rates, offering real-time insights into individual physiological responses to environmental conditions. Sweat and core body temperature are critical indicators of physiological responses to heat stress. Monitoring these factors helps assess individual heat tolerance, hydration, and risk of heat-related illnesses, informing public health interventions and early warning systems to mitigate health risks and improve community resilience to climate change.

Utilizing wearable devices in climate change and health research offers numerous advantages for understanding population health. These devices enable continuous, non-invasive monitoring of individuals’ physiological parameters, reflecting their responses to environmental stressors. By collecting real-time data at scale, researchers can identify patterns, trends, and vulnerabilities among different population groups. This granular data helps to uncover hidden health risks and provides insights into the varied effects of climate change on human health. Furthermore, wearables facilitate the study of underrepresented populations in resource-limited settings, contributing to a more comprehensive understanding of global health challenges. Ultimately, the use of wearable devices in climate change and health research can guide evidence-based interventions and inform policies aimed at mitigating the health impacts of climate change on diverse populations.

#### 4.1.2. Morbidity assessment

In Burkina Faso, the population faces considerable health challenges due to heat and erratic rainfall patterns associated with climate change (55, 56). Research suggests that Africa’s surface temperature is likely to increase at a faster rate than the global average (57), and the altered precipitation patterns are already causing severe droughts and floods (23). These extreme heat and unpredictable precipitation events result in a heightened risk of heat-related illnesses, compromised agricultural productivity leading to food insecurity and malnutrition, and the proliferation of vector-borne diseases due to altered breeding habitats (58–61). A comprehensive understanding of these outcomes is crucial for developing effective, evidence-based policies and interventions to mitigate the impacts of climate change on the health of the population in Burkina Faso and similar settings (62). Nevertheless, there is a scarcity of targeted research, and the effective utilization of surveillance data is essential for monitoring the health impacts of climate change-induced exposures in Africa and the sub-Saharan region (63).

To address this research gap, the CHEERS system incorporates a module designed for routine morbidity data collection in a 10% sample of all households, providing a feasible and cost-effective approach. This module captures information on acute and chronic diseases, symptom onset, and the extent of disability caused by these conditions. Accurately quantifying health impacts attributable to climate change by calculating metrics such as DALYs or quality-adjusted life years (QALYs) is essential for estimating loss and damages (64). The calculation of climate-associated DALYs (cDALYs) and QALYs (cQALYs) requires attributing weather events to climate change (65) and establishing the link between health outcomes and weather exposures, ultimately connecting them to climate change (66). Quantitative L&D research has focused mainly on climate risk and attribution, with less attention paid to empirical data of L&D connected to attributed climate events (67). Significant progress has been made in these areas, and further advancements are anticipated with efforts in place including the Santiago Network,^4^ which coordinates and facilitates Loss and Damage (L&D) needs assessments, and the Warsaw International Mechanism^5^ on L&D, which addresses the impacts of climate change in developing countries. The international L&D policy debate revolves around supporting vulnerable developing countries in averting, minimizing, and addressing climate change impacts (68). Human-caused climate change increases the frequency and severity of extreme weather events and slow-onset events, leading to L&D. Reducing L&D involves enhancing resilience, offering financial or social protection support, and incorporating resilience into recovery efforts. Broader policy and governance arrangements play a crucial role in decreasing vulnerability and exposure to climate change, with sustainable development serving as a key component in this process. This information is vital for international negotiations and policy governance to effectively address the consequences of climate change on vulnerable populations.

In the future, data collection efforts within the Nouna CHEERS site will be expanded by incorporating additional physical examinations, encompassing body temperature, blood pressure, pulse, fasting plasma glucose, glycated hemoglobin (HbA1c), hemoglobin levels, and malaria diagnostics. This component has not yet been developed and implemented; however, its integration can be facilitated through capacity building among healthcare workers, including community health workers, by training them to perform the necessary tests. Furthermore, the use of point-of-care testing devices can support testing in remote and resource-limited settings by offering immediate results to inform patient management. These devices are advantageous due to their portability, ease of use, and minimal dependence on laboratory infrastructure, making them a suitable option for expanding data collection efforts in challenging environments.

### 4.2. Climate-related data

#### 4.2.1. Indoor temperature

At present, our knowledge of individual indoor exposures in low-resource settings and the corresponding adaptive approaches to address heat stress is limited (69). For example, in the Nouna CHEERS site, residential structures are frequently constructed using cost-effective materials, such as corrugated metal sheets, which may lead to elevated indoor temperatures for occupants. Consequently, those residing in substandard housing are likely to face challenges in accessing adaptive measures, such as air conditioning, exacerbating their vulnerability to heat stress (70). Elevated indoor temperature exposures can result in various physiological responses, including alterations in heart rate as a consequence of heat exposure (71). Nonetheless, the scarcity of research exploring the associations between indoor temperature and the health of at-risk populations might contribute to the marginalization of these communities while simultaneously obscuring potential health hazards (71–73). Acquiring additional data is crucial for quantifying potential risks and determining response priorities. Therefore, CHEERS integrates indoor measurements utilizing compact sensors (currently employing iButton DS1923-F5 and Switchbot Meter) that can be installed in households and remain in place for extended periods due to their long battery life and unobtrusive design.

#### 4.2.2. Remotely-sensed data

Remote sensing may generate valuable insights into various environmental factors and their impacts on public health through its continuous, global coverage and availability. Applications of remote sensing include air quality monitoring, identification of heat islands and areas prone to heat stress through land surface temperature measurements, tracking of environmental conditions associated with vector-borne diseases, assessment of water quality and waterborne diseases, and monitoring of agricultural productivity and crop yields for food security and malnutrition studies (43, 44). Many remote sensing datasets are freely available for public use, although the accessibility and resolution of the data may vary along with the usability per use case or application. Numerous space agencies, research institutions, and other organizations provide open access to remote sensing data collected by Earth observation satellites, including NASA Earth Observing System Data and Information System (EOSDIS),^6^ United States Geological Survey (USGS) EarthExplorer,^7^ European Space Agency (ESA),^8^ and NOAA National Centers for Environmental Information (NCEI).^9^

In our research conducted in the Nouna area, we developed models to predict agricultural field yields based on 3 years of *in situ* data (43). These models successfully captured a portion of the inter-annual variability in yields, potentially reducing the need for extensive measurements in the future. That also reveals one downside of these remote sensing-based models, as they need extensive *in situ* data to train and calibrate the models, which ideally also includes long time series of field data. Nevertheless, these models facilitated the integration of individual household yields with socioeconomic variables, child nutrition, and family health, demonstrating the potential of high-resolution field yield estimation in public health research. Remotely sensed data on the household field level may provide important data for research into agricultural, child undernutrition, and heat impact on work productivity – particularly, farmer work productivity, as the majority of households in Nouna rely on rainfed, small-scale subsistence farming. Results could inform efficient interventions and policies, such as irrigation systems.

#### 4.2.3. Weather stations

Weather stations play a crucial role in understanding the effects of environmental exposures on health; however, their availability is often limited to central locations such as airports, stations outside major metropolitan areas, military locations, or derived from satellite-based precipitation estimates like CHIRPS (Climate Hazards group Infrared Precipitation with Stations) (37). The latter can be too distant or have too coarse resolution to accurately attribute weather exposures to individual health. To accurately evaluate the impact of weather exposures on health, especially in the context of climate change, it is imperative to establish spatially distributed weather stations with high temporal resolution in LMICs. Ideally, these weather stations should be long-lasting, low-cost, and easily maintainable to ensure homogeneous longitudinal datasets, adequate spatial coverage of study regions, and high on-site maintainability. Although fully automated weather stations have been successfully deployed in the Nouna CHEERS site and the Siaya HDSS (Kenya) (42), which has implemented initial CHEERS components, their high cost (approximately 15,000€ per station) and reliance on importation present scalability challenges. As an alternative, 3D-printed weather stations offer a cost-effective, locally producible option. A pilot project in the SEACO HDSS in Malaysia, which is also currently establishing first CHEERS components, is currently evaluating the reliability, ease of data collection, and maintainability of 3D-printed weather stations within the CHEERS framework. Utilizing validated blueprints from the 3D-PAWS initiative (74), 3D-printed weather stations can be constructed with 3D-printed components and local hardware for 600–1,000€ per station. This approach reduces import costs and increases local capacity and ownership. Integrating weather stations with research infrastructures such as HDSSs can significantly advance climate change and health research, enabling targeted public health interventions in LMICs. We hypothesize that the reduced cost burden, simplified maintenance processes, and increased sense of ownership associated with 3D-printed weather stations will address key barriers to their establishment, enabling more extensive data collection on a finer spatial scale. This would generate valuable climate change data to better understand and address the priorities of vulnerable populations, for which limited data currently exists.

### 4.3. Graph database for holistic data management

Data collection and storage in sub-Saharan environments can be limited, fragmented, and tailored for specific applications (75). Accurate, reliable, and interoperable datasets are vital for effective data management and analysis. The CHEERS framework addresses this need by importing routinely collected data into a graph database, an advanced tool for exploring climate change and health challenges in vulnerable populations.

Graph databases offer advantages over traditional relational databases, including faster analysis, broader data type management, and structural flexibility (76, 77). They enable researchers to uncover previously unnoticed patterns, trends, and associations, contributing to a better understanding of climate change’s impact on at-risk populations.

By making CHEERS datasets publicly available, AI-based research in sub-Saharan Africa can advance, overcoming data limitations that have hindered progress (75). However, data ownership and sharing regulations present challenges. It is essential to establish governance processes that protect individual privacy while promoting data access. Public health organizations can facilitate data sharing by implementing guidelines and processes, although further research and dialogue are needed to determine optimal strategies for data sharing.

Comprehensive graph datasets offer unique value to CHEERS sites, enabling integration of additional research and potentially attracting funding. Accessible datasets encourage scientific investigation and interdisciplinary collaboration, highlighting the importance of recognizing their value for fostering scientific advancement.

### 4.4. Dashboards

The timely sharing of research insights, particularly from data collected using digital devices such as wearables, weather stations, and indoor temperature sensors, can be expedited through the use of dashboards. These dashboards provide data visualizations that can effectively illustrate the connections between climate change and health. By translating complex data into visually appealing and user-friendly formats, dashboards enable a wider audience, including non-experts, policymakers, and the general public, to access and understand the information. Real-time updates, interactivity, and the integration of data from multiple sources contribute to a comprehensive and dynamic representation of research findings.

Dashboards can also facilitate evidence-based decision-making by offering insights into the relationships between climate change, health risks, and other relevant factors. This allows policymakers to develop informed strategies for adaptation and mitigation. Online dashboards with narratives can increase population awareness and potentially promote behavior change, as meaningful narratives in health communication may be more effective than mere factual statements.

However, data privacy and security are crucial concerns when dealing with sensitive information, especially personal biometric data. Dashboards must be designed with strict access controls and data protection measures to prevent unauthorized access and data breaches. Additionally, the accuracy and reliability of the collected data depend on the quality of the devices and the data collection methods employed.

While aggregated and anonymized data can be displayed on dashboards, careful consideration should be given to the potential risks associated with making personal biometric information accessible to the general public. Any decision to display individually collected real-time biometric data on public platforms should be taken with caution.

Currently, we are implementing CHEERS components in the SEACO HDSS in Malaysia and the Siaya HDSS in Kenya. To our knowledge, there are no other studies that have adopted a framework comparable to CHEERS, although some have integrated individual elements such as remote sensing data or weather stations. The novelty of the CHEERS framework stems from the synthesis of these data sources, facilitating a comprehensive assessment of climate and health impacts at the household level. Moreover, although pending evaluation, our experience suggests that the system is designed to be optimally incorporated into other existing HDSSs, as well as similar research infrastructures in any country, regardless of income level – low, middle, or high-income – showcasing its versatility and adaptability to diverse settings.

### 4.5. Limitations

While the CHEERS framework presents a promising approach to understanding the impact of climate change on health, there are several limitations that must be acknowledged.

- Context-specific adaptation: The CHEERS framework has been pioneered in the Nouna HDSS in a sub-Saharan environment. As it is implemented in other settings, such as the SEACO HDSS in Malaysia and the Siaya HDSS in Kenya, it may be necessary to revise some components to better fit into different contexts, taking into account local environmental, socio-cultural, and infrastructural factors.
- Cost analysis: A thorough cost analysis of the CHEERS framework has not been conducted. Although it is anticipated that the operating costs of CHEERS will be lower than those of an annual HDSS data collection with multiple rounds, a detailed assessment of the costs involved is needed to provide a more accurate comparison.
- Community engagement: While current HDSSs focus primarily on promoting community participation and translation, it would be ideal to provide guidelines for communities to be involved in an organized and regular way, allowing them to provide feedback in defining research programs and identifying possible areas of priority.
- Emergency data access: Investigating how the collected data could be made available in emergency situations, such as for disaster management, may be beneficial. Further exploration is required to develop protocols and systems that allow for rapid data access while maintaining data privacy and security.
- Integration of additional physical examinations: Although the paper outlines plans to expand data collection efforts within the Nouna CHEERS site to include additional physical examinations, this component has not yet been developed and implemented. The integration of these additional data sources will require capacity building among healthcare workers and may necessitate the use of point-of-care testing devices suitable for remote and resource-limited settings.
- Pilot testing limitations: Some components of the CHEERS framework, such as the dashboard and the AI exploratory approach for the graph-based database, are in the pilot testing phase. As these components are essential for data analysis and visualization, their effectiveness and usability need to be evaluated and refined based on the pilot testing outcomes. Further results and improvements regarding these components will be shared in forthcoming publications.

Addressing these limitations in future iterations of the CHEERS framework will help ensure its adaptability and effectiveness in a range of settings and contribute to a more comprehensive understanding of climate change and health impacts.

## 5. Conclusion

The Climate and Health Surveillance and Response System (CHEERS) framework offers an innovative approach to expand existing HDSSs, generating crucial climate change and health-related data. It incorporates advanced technologies, such as wearable devices, indoor temperature and humidity sensors, automated weather stations, remote sensing, graph databases, and interactive dashboards, enhancing routine health and demographic data collection efficiency.

Successfully implemented in Nouna HDSS and adopted by Siaya HDSS and SEACO HDSS, CHEERS streamlines data collection, transfer, and management, making research infrastructures more attractive to researchers and funders. It enables flexible management of complex population-health data, integrating various data types at different resolutions.

CHEERS has the potential to significantly advance climate change and health research in LMICs, identifying priority areas and informing evidence-based interventions and policies. Further research is needed to evaluate its costs, benefits, and effectiveness across diverse settings.

## Data availability statement

The raw data supporting the conclusions of this article will be made available by the upon request, without undue reservation.

## Ethics statement

The studies involving human participants were reviewed and approved by the Ethics Committee at Heidelberg University Hospital and the Comité d’Ethique pour la Recherche en Santé. Written informed consent to participate in this study was provided by the participants’ legal guardian/next of kin. Written informed consent was obtained from the individual(s), and minor(s)’ legal guardian/next of kin, for the publication of any potentially identifiable images or data included in this article.

## Author contributions

SB wrote the first draft of the manuscript with the assistance of RS. Data collection and local study management are locally managed and conducted by IT, WO, VB, PZ, SM, SK, and DO, and supervised by TS. All author contributed significant intellectual substance to the written protocol and approved the final version for publication.

## Conflict of interest

The authors declare that the research was conducted in the absence of any commercial or financial relationships that could be construed as a potential conflict of interest.

## Publisher’s note

All claims expressed in this article are solely those of the authors and do not necessarily represent those of their affiliated organizations, or those of the publisher, the editors and the reviewers. Any product that may be evaluated in this article, or claim that may be made by its manufacturer, is not guaranteed or endorsed by the publisher.

## Footnotes

^1^ https://www.crsn-nouna.bf/

^2^ https://dataspace.copernicus.eu/index.html#data-access

^3^ http://www.indepth-network.org/Resource%20Kit/INDEPTH%20DSS%20Resource%20Kit/Informed_consent.htm

^4^ https://unfccc.int/santiago-network

^5^ https://unfccc.int/topics/adaptation-and-resilience/workstreams/loss-and-damage/warsaw-international-mechanism

^6^ https://www.earthdata.nasa.gov/

^7^ https://earthexplorer.usgs.gov/

^8^ https://earth.esa.int/eogateway

^9^ https://www.ncei.noaa.gov/
