# Supplementary material for: Widening the lens of population-based health research to climate change impacts and adaptation: the climate change and health evaluation and response system (CHEERS)
Source: Front Public Health. 2023 May 25;11:1153559. doi: 10.3389/fpubh.2023.1153559 (PMC10248881; doi:10.3389/fpubh.2023.1153559)
Supplement: Supplementary file 1 [file Data_Sheet_1.docx]

**Appendix 1**

Overview of all HDSS as listed on the INDEPTH website (status quo: September 2022), comprising a total of 56 listed HDSS in 21 different countries.

| **Country and existing HDSSs** | **No. of HDSS** | **Population under surveillance** | **Villages under surveillance** | **Size of HDSS (km^2^)** | **Start year of HDSS** |
| --- | --- | --- | --- | --- | --- |
| **Bangladesh** | **4** | **482.641** |  |  |  |
| Bandarban HDSS |  | 19.403 | - | - | - |
| Chakaria HDSS |  | 120.000 | 183 | 288 | 1999 |
| Dhaka HDSS |  | 118.238 | - | - | - |
| Matlab HDSS |  | 225.000 | 142 | 184 | 1966 |
| **Burkina Faso** | **5** | **296.907** |  |  |  |
| Nouna HDSS  Nanoro HDSS  Kaya HDSS |  | 54.780  64.480 | 25 |  | 2007 |
| Ouagadougou HDSS |  | 90.000 | 5 | NA | 2008 |
| Sapone HDSS (2011) |  | 87.647 | NA | 1700 | # |
| **Ethiopia** | **7** | **520.884** |  |  |  |
| [Arba Minch HDSS](http://www.indepth-network.org/member-centres/arba-minch-hdss)  Butajira HDSS  Dabat HDSS  Gilgel Gibe HDSS  Harar HDSS  Kersa HDSS  Kilite Awlaelo |  | 68.802  78.000  69.468  62.235  NA  129.200  65.848 |  |  |  |
| **Gambia** | **2** | **47.331** |  |  |  |
| Farafenni HDSS  West Kiang HDSS |  | 47.331 |  |  |  |
| **Ghana** | **3** | **432.402** |  |  |  |
| Dodowa HDSS  Kintampo HDSS  Navrongo HDSS |  | 132.690  142.977  156.735 |  |  |  |
| **Guinea-Bissau** | **1** | **105.000** |  |  |  |
| Bandim HDSS |  | 105.000 | 182 | - | 1978 |
| **India** | **3** | **240.274** |  |  |  |
| Ballabgarh HDSS |  | 90.240 | - |  | 1988 |
| Birbhum HDSS |  | 59.395 | 351 | - | 1961 |
| Vadu HDSS |  | 90.639 | 22 | - | 1977 |
| **Indonesia** | **1** | **12.218** |  |  |  |
| Purworejo HDSS |  | 12.218 | - | - | 1994 |
| **Ivory Coast** | **1** | **42.480** |  |  |  |
| Taboo HDSS |  | 42.480 |  |  |  |
| **Kenya** | **5** | **729.165** |  |  |  |
| Kilifi HDSS  Kisumu HDSS  Kombewa HDSS  Mbita HDSS  Nairobi HDSS |  | 260.000  230.000  123.456  54.014  61.695 |  |  |  |
| **Malawi** | **1** | **35.730** |  |  |  |
| Karonga HDSS |  | 35.730 |  |  |  |
| **Malaysia** | **1** | **40.000** |  |  |  |
| SEACO HDSS |  | 40.000 | - | 1.250 | 2011 |
| **Mozambique** | **2** | **189.451** |  |  |  |
| Chókwè HDSSManhica HDSS |  | 99.834  89.617 | - | - | 2010 |
| **Nigeria** | **2** | **167.230** |  |  |  |
| Cross River HDSS  Nahuche HDSS |  | 31.124  136.106 |  |  | 2010 |
| **Papua New Guinea** | **1** | **56.000** |  |  |  |
| PiH HDSS  Wosera HDSS |  | 56.000  NA |  |  |  |
| **Senegal** | **3** | **64.573** |  |  |  |
| Bandafassi HDSS  Mlomp HDSS  Niakhar HDSS |  | 13.373  8.200  43.000 | 42 | - | 1970 |
| **South Africa** | **3** | **318.000** |  |  |  |
| Agincourt HDSS |  | 115.000 | 27 | 420 | 1992 |
| AHRI HDSS |  | 168.000 | - | 840 | 2000 |
| Dikgale HDSS |  | 35.000 |  |  | 1995 |
| **Tanzania** | **3** | **NA** |  |  |  |
| Magu HDSS  Ifakara HDSS  Rufiji HDSS |  | NA  NA  NA |  |  |  |
| **Thailand** | **1** | **NA** |  |  |  |
| Kanchanaburi HDSS |  | NA |  |  |  |
| **Uganda** | **3** | **151.794** |  |  |  |
| Iganga/Mayuge  Kyamulibwa HDSS  Rakai HDSS |  | 79.794  22.000  50.000 |  |  |  |
| **Vietnam** | **2** | **92.395** |  |  |  |
| DodaLab HDSS |  | 40.598 | - | - | 2007 |
| Filabavi HDSS  Chililab HDSS |  | 51.797  NA | - | 424 | 1999 |
| **Total** | **49** | **4.024.475** |  |  |  |
