# Supplementary material for: Widening the lens of population-based health research to climate change impacts and adaptation: the climate change and health evaluation and response system (CHEERS)
Source: Front Public Health. 2023 May 25;11:1153559. doi: 10.3389/fpubh.2023.1153559 (PMC10248881; doi:10.3389/fpubh.2023.1153559)
Supplement: Supplementary file 2 [file Data_Sheet_2.pdf]

Generated by sandrabarteit, Nov 14, 2022 14:07
Questionnaire created by sandrabarteit, Nov 14, 2022 14:07
Last modified by sandrabarteit, Nov 14, 2022 14:07

Not shared with anyone

Sections: 17, Sub-sections: 12,
Questions: 519.
Questions with enabling conditions: 283
Questions with validation conditions:74
Rosters: 38
Variables: 54

Survey Solutions

CHES-RS

SURVEY IDENTIFICATION INFORMATION
QUESTIONNAIRE DESCRIPTION

COVER
No sub-sections, No rosters, Questions: 4.

INTRODUCTION
No sub-sections, No rosters, Questions: 23, Static texts: 5, Variables: 13.

IDENTIFICATION MÉNAGE
Sub-sections: 3, Rosters: 1, Questions: 47, Static texts: 1, Variables: 5.

MEMBRES DU MÉNAGE
Sub-sections: 5, Rosters: 4, Questions: 99, Static texts: 17, Variables: 14.

NUTRITION
No sub-sections, Rosters: 2, Questions: 61, Static texts: 3, Variables: 1.

INSÉCURITÉ ALIMENTAIRE
No sub-sections, Rosters: 1, Questions: 18.

ANTHROPOMÉTRIE
Sub-sections: 1, Rosters: 1, Questions: 22, Static texts: 4, Variables: 1.

REVENUS ET DÉPENSES
No sub-sections, Rosters: 6, Questions: 20, Static texts: 5.

MORBIDITÉ
Sub-sections: 1, Rosters: 10, Questions: 57, Static texts: 10, Variables: 8.

AGRICULTURE AUX CHAMPS
No sub-sections, Rosters: 2, Questions: 26, Static texts: 4, Variables: 1.

LES CHAMPS DU MÉNAGE
No sub-sections, Rosters: 2, Questions: 15, Variables: 4.

LES CULTURES SPÉCIFIQUES
Sub-sections: 1, Rosters: 3, Questions: 40, Static texts: 1.

HANDICAPS
No sub-sections, Rosters: 2, Questions: 19.

ÉMIGRATION
No sub-sections, Rosters: 1, Questions: 28, Static texts: 1, Variables: 2.

IMMIGRATION
Sub-sections: 1, Rosters: 2, Questions: 29, Static texts: 1, Variables: 2.

DÉCÈS
No sub-sections, Rosters: 1, Questions: 10, Static texts: 3, Variables: 1.

TEST GPS

No sub-sections, No rosters, Questions: 1, Static texts: 3, Variables: 2.

APPENDIX A — ENABLING CONDITIONS

APPENDIX B — VALIDATION CONDITIONS AND MESSAGES

APPENDIX C — CATEGORIES

APPENDIX D — VARIABLES

APPENDIX E — CATEGORIES FILTERS

LEGEND

SURVEY IDENTIFICATION INFORMATION QUESTIONNAIRE DESCRIPTION

Basic information

Title	CHES-RS
Subtitle	Census de recensement HDSS Nouna
Version identificator	ver.1

Survey data information

Study type	Demographic and Health Survey
Kind of data	Census/enumeration data [cen]
Mode of Data Collection	CAPI

Survey information

Country	Burkina Faso
Year	2019
Languages	French
Unit of analysis	Household
Coverage	HDSS Nouna
Funding	HIGH - Universitätsklinikum Heidelberg

COVER

Ménage	TEXT showLocation<br>....................................
Chef Ménage	TEXT showFirstCHM<br>....................................
Sélectionné:<br>I Round 1: 10% sample for asking Morbitié & Revenus/Dépenses	SINGLE-SELECT round1sampled<br>01 ○ Oui<br>02 ○ Non
Coordonnées	GPS location<br>- - - - - - - - - - - -<br>N<br>- - - - - - - - - - - -<br>W<br>- - - - - - - - - - - -<br>A

INTRODUCTION

VARIABLE<br>false	BOOLEAN test<br>This variable is excluded from the exported data
Country<br>E test==true	SINGLE-SELECT tcountry<br>01 ○ Burkina Faso<br>02 ○ Kenya
VARIABLE<br>1 /* 1 Burkina */ /* 2 Kenya */	LONG country
Type of survey?<br>E test==true	SINGLE-SELECT tsurvey<br>01 ○ HDSS<br>02 ○ P1/P2
VARIABLE<br>1 /* 1 HDSS2.0 2 P1/P2 */	LONG survey
VARIABLE<br>"#ff8800"	STRING colorEmphasis
VARIABLE<br>1	LONG round
VARIABLE<br>false	BOOLEAN newHousehold
TEMPORARY PRELOADED ROUND 1: "HH had Bi rths After the Census" flag (1 or NULL)	TEXT bac<br>SCOPE: HIDDEN<br>....................
Date of previous visit	DATE dateLastVisit<br>SCOPE: HIDDEN<br>....................
VARIABLE<br>IsAnswered(dateLastVisit) ? dateLastVisit : new DateTime (2019, 10, 1) /* start of census */	DATETIME dateBase
VARIABLE<br>dateBase.Value.Date.ToString("dd MMMM yyyy")	STRING dateBasePretty
Assignment ID<br>E false	TEXT assignment_id<br>....................
Commune<br>E false	SINGLE-SELECT comm<br>SCOPE: HIDDEN<br>01 ○ Nouna<br>02 ○ Bourasso

**Village**<br><br>I Tapez partie du nom du village, et sélectionnez de la liste	SINGLE-SELECT: COMBO BOX<br>SCOPE: HIDDEN — vill<br>02 ○ Toni<br>05 ○ Boron<br>08 ○ Cissé<br>09 ○ Dankoumana<br>10 ○ Dembeléla<br>11 ○ Denissa<br>12 ○ Denissa Mossi<br>14 ○ Dionkongo<br>15 ○ Dina<br>16 ○ Dokoura<br>17 ○ Goni<br>18 ○ Kamadena<br>19 ○ Kèmena<br>21 ○ Koro<br>23 ○ Leï<br>27 ○ Ouetté<br><br>And 43 other symbols [2]
**Nom du nouveaux village**<br><br>E `vill >= 990`	TEXT — nouveau_village<br><br>..
**Milieu de Residence**	SINGLE-SELECT — milieu<br>01 ○ Urbain<br>02 ○ Rural
**Quartier/Secteur**<br><br>I Écrire 00 s'il n'y a pas des secteurs.	TEXT<br>SCOPE: HIDDEN — quartier<br><br>..
**Numero Concession**<br><br>I 9999 - si c'est une nouvelle concession	TEXT<br>SCOPE: HIDDEN — numConcession<br><br>..
VARIABLE<br>`vill + quartier + numConcession`	STRING — idConcession
**Id Ménage**	TEXT<br>SCOPE: HIDDEN — idMenage<br><br>..
**Appuyer pour enregistrer la date**	DATE: CURRENT TIME — dateEntree<br><br>..
**Avez-vous activé le Mode Avion de la tablette?**<br>S'il vous plait verifiez et l'activez!	SINGLE-SELECT — airplaneMode<br>01 ○ Oui<br>02 ○ Non

STATIC TEXT

E airplaneMode==2

Si vous ne savez pas comment activer le Mode Avion, demandez votre superviseur.

Appuyer pour enregistrer les coordonnées GP S	GPS gps<br>N<br>W<br>A
VARIABLE<br>gps.Accuracy	DOUBLE gps_accuracy

STATIC TEXT

GPS Accuracy: %gps_accuracy%

VARIABLE<br>50	DOUBLE maxGPSdifference
VARIABLE<br>(double)Math.Round((decimal)gps.GpsDistance(location),0)	DOUBLE GPSdist
VARIABLE<br>(double)Math.Round((decimal)GPSdist/1000,0)	DOUBLE GPSdistKm

STATIC TEXT

E GPSdist>maxGPSdifference && GPSdist<1000

Tu es %GPSdist% m de la position enregistrée pour cette concession!

STATIC TEXT

E GPSdist>maxGPSdifference && GPSdist>=1000

Tu es %GPSdistKm% km de la position enregistrée pour cette concession!

STATIC TEXT

E !IsAnswered(gps) && !(IsAnswered(latitudeManual) && IsAnswered(longitudeManual))

Aucune mesure valide de GPS

Voulez vous enregistrer les coordonnées GPS manuellement?<br>E !IsAnswered(gps)	SINGLE-SELECT gpsExterne<br>01 ○ Oui<br>02 ○ Non
Latitude<br>E gpsExterne==1	NUMERIC: DECIMAL latitudeManual
Longitude<br>E gpsExterne==1	NUMERIC: DECIMAL longitudeManual

Question	Réponse	Variable
L'interview peut être réalisé?	SINGLE-SELECT<br>01 Oui, interview peut être réalisé<br>02 Non - Refusé<br>03 Non - ménage n'a pas pu être trouvé<br>04 Non - il n y avait personne au ménage<br>05 Non - le ménage n'a pas pu être accédé<br>06 Non - tout le ménage a déménagé<br>07 Non - tout le ménage est décédé<br>09 Non - autre raison	result
Pourquoi pas?<br>E `result.InList(5,9)`	TEXT<br>..	result_autre
Ou est-ce que le ménage a déménagé?<br>E `result == 6`	SINGLE-SELECT<br>01 autre endroit dans le HDSS<br>02 Burkina Faso - hors du HDSS<br>03 hors Burkina Faso - Exterieur	demenage
Quelle village?<br>E `demenage==1`	SINGLE-SELECT: COMBO BOX<br>02 Toni<br>05 Boron<br>08 Cissé<br>09 Dankoumana<br>10 Dembeléla<br>11 Denissa<br>12 Denissa Mossi<br>14 Dionkongo<br>15 Dina<br>16 Dokoura<br>17 Goni<br>18 Kamadena<br>19 Kèmena<br>21 Koro<br>23 Leï<br>27 Ouetté<br><br>And 43 other symbols [1]	villageDestin

Le consentement éclairé est-il signé ?<br>E result==1	SINGLE-SELECT consentement<br>01 O Oui<br>02 O Non

IDENTIFICATION MÉNAGE

E $OK1

VARIABLE<br>"blue"	STRING colorName
Quel é le nom du Chef de Ménage?<br>E !(continuation==true) &&false	TEXT nomChefMenage
Est-ce que %showFirstCHM% est le Chef de Mé nage?<br>E !(continuation==true)	SINGLE-SELECT confirmCHM<br>01 Oui<br>02 Non
Selectionnez le nouveau Chef de Ménage<br>(Si la personne n'est pas dans la liste, allez a la [liste des membres](membres) e ajoutez cette personne!)<br>E confirmCHM == 2	SINGLE-SELECT: LINKED nouveauCHM<br>SOURCE OF CATEGORIES: ROSTER MEMBRE
Code Ménage<br>E // /* for P1P2 -> */ false true	TEXT codeMenage<br>SCOPE: HIDDEN
VARIABLE<br>idConcession + codeMenage	STRING new_idMenage
VARIABLE<br>codeMenage.ToUpper().ToCharArray()[0] == 'X' || codeMenage.ToUpper().ToCharArray()[0] == 'Y' || codeMenage.ToUpper().ToCharArray()[0] == 'Z'	BOOLEAN continuation
VARIABLE<br>continuation==true ? "(continu)" : "de"	STRING continuationMark
Numéro de téléphone du Chef de Ménage<br>E continuation!=true	TEXT phoneChefMenage
Nombre Individus (MAS)<br>E continuation!=true &&false	NUMERIC: INTEGER numIndividu_mas
Nombre Individus (FEM)<br>E continuation!=true &&false	NUMERIC: INTEGER numIndividu_fem

IDENTIFICATION MÉNAGE

HABITAT ET CONDITIONS DE VIE

E continuation!=true && !$P1P2

Question	Réponse	Variable
Quel est le statut d’occupation du logement pri ncipale?	SINGLE-SELECT<br>01 ○ Propriétaire<br>02 ○ Locataire<br>03 ○ Logé par employeur<br>04 ○ Logé par parents/Amis<br>05 ○ Autre	q102
Quel autre statut?<br>E q102==5	TEXT<br>..	q102autre
Quel est le type d’habitat du logement principal du ménage ?	SINGLE-SELECT<br>01 ○ Case ronde<br>02 ○ Maison isolée simple<br>03 ○ Villa/Mini-villa<br>04 ○ Bâtiment à plusieurs logem ents<br>05 ○ Immeuble à appartements<br>06 ○ Autres (à préciser)	q101
Quel autre type?<br>E q101==6	TEXT<br>..	q101autre
Quel est le nombre de pièces du logement?	NUMERIC: INTEGER<br>_ _ _ _ _ _ _ _ _ _ _ _ _ _ _ _ _ _ _ _	q105
Quelle est la nature du matériau principal du m ur de l’habitation principale du ménage ?	SINGLE-SELECT<br>01 ○ Dur<br>02 ○ Semi-dur<br>03 ○ Banco<br>04 ○ Paille<br>06 ○ Pierre en carrière /cailloux<br>05 ○ Autre	q106
Quel autre?<br>E q106==5	TEXT<br>..	q106autre
De quels principaux matériaux est fait le sol de l’habitation principale du ménage ?	SINGLE-SELECT<br>01 ○ Moquette / parquet<br>02 ○ Bois poli<br>03 ○ Carreaux<br>04 ○ Vinyle<br>05 ○ Ciment<br>06 ○ Terre battue / Sable<br>09 ○ Autre (à préciser)	q107

Quels autres matériaux?<br><br>E q107==9	TEXT q107autre<br><br>..
Quels sont les principaux matériaux du <u>toit</u> de l'habitation principale du ménage ?	SINGLE-SELECT q108<br>01 ○ Béton<br>02 ○ Tuiles<br>03 ○ Tôles<br>04 ○ Paille/Feuille<br>05 ○ Banco / Terre Battue<br>06 ○ Autre
Quels autres matériaux?<br><br>E q108==6	TEXT q108autre<br><br>..
Quel type de toilettes utilisez-vous?<br><br>I Réponses multiples - commencez avec celui qui est utilisé le plus frequemment	MULTI-SELECT: ORDERED q109<br>01 ☐ WC avec chasse eau<br>02 ☐ Latrines aménagées<br>03 ☐ Latrines non aménagées<br>04 ☐ Pas de toiulette / nature<br>05 ☐ Autre
Quel autre type?<br><br>E `q109.Contains(5)`	TEXT q109autre<br><br>..
Quelle principale source d'<u>énergie de cuisson</u> utilisez-vous?	SINGLE-SELECT q110<br>01 ○ Electricité / Gaz<br>02 ○ Réchaud à pétrole<br>03 ○ Charbon de bois<br>04 ○ Bois à bruler<br>05 ○ Paille<br>06 ○ Bouse<br>09 ○ Autre
Quelle autre source?<br><br>E q110==9	TEXT q110autre<br><br>..

Quelle principale source d'<u>énergie d'éclairage</u> utilisez-vous?	SINGLE-SELECT q110a<br>01 ○ Electricité Sonabel<br>02 ○ Groupe électrogène<br>03 ○ Plaque solaire<br>04 ○ Batterie<br>05 ○ Lampe à gaz<br>06 ○ Lampe à pétrole<br>07 ○ Lampe à huile<br>08 ○ Lampe torche<br>09 ○ Bougie<br>10 ○ Bois / paille<br>99 ○ Autre
Quelle autre source?<br>E q110a==99	TEXT q110aautre<br>..
Quelle est la source principale d'eau de boisson en saison sèche ?<br>I choix multiples – commencez avec celui qui est utilisé le plus fréquemment.	SINGLE-SELECT q111<br>01 ○ Puits creusé non protégé<br>02 ○ Source non protégée<br>03 ○ Camion citerne/charrette avec petite citerne<br>04 ○ Eau de surface<br>10 ○ Eau en bouteille/sachet<br>11 ○ Robinet dans logement/concession<br>12 ○ Robinet public/fontaine<br>13 ○ Puits à pompe/forage<br>14 ○ Puits creusé protégé<br>15 ○ Source d'eau protégée<br>99 ○ Autre
Quelle autre eau de boisson?<br>E q111==99	TEXT q111autre<br>..

Quelle est la source principale d'eau de boisson en saison pluvieuse ?<br><br>I choix multiples – commencez avec celui qui est utilisé le plus fréquemment.	SINGLE-SELECT q112<br>01 ○ Puits creusé non protégé<br>02 ○ Source non protégée<br>03 ○ Camion citerne/charrette avec petite citerne<br>04 ○ Eau de surface<br>10 ○ Eau en bouteille/sachet<br>11 ○ Robinet dans logement/concession<br>12 ○ Robinet public/fontaine<br>13 ○ Puits à pompe/forage<br>14 ○ Puits creusé protégé<br>15 ○ Source d'eau protégée<br>99 ○ Autre
Quelle autre eau de boisson?<br><br>E q112==99	TEXT q112autre<br>..
Temps de trajet pour s'approvisionner en eau de boisson	SINGLE-SELECT tempEau<br>01 ○ Eau sur place<br>02 ○ Moins de 30 minutes<br>03 ○ 30 minutes ou plus<br>09 ○ Ne sait pas
Quel est la distance qui sépare votre ménage à la source d'eau de boisson?	SINGLE-SELECT distEau<br>01 ○ Sur place<br>02 ○ Moins d'un km<br>03 ○ 1 à 2 km<br>04 ○ Plus de 2 km

IDENTIFICATION MÉNAGE

ÉQUIPMENTS DU MÉNAGE

E continuation!=true

Question	Réponse
Le ménage possède-t-il un de ces équipements ?<br><br>E !$P1P2	MULTI-SELECT: YES/NO — q200<br>201 ☐/☐ RADIO<br>202 ☐/☐ TELEVISEUR<br>203 ☐/☐ LECTEUR VIDEO/CD/DVD<br>204 ☐/☐ TELEPHONE FIXE<br>205 ☐/☐ TELEPHONE PORTABLE<br>218 ☐/☐ MOTO<br>219 ☐/☐ TRICYCLE<br>220 ☐/☐ BICYCLETTE (VÉLO)<br>206 ☐/☐ REFRIGERATEUR / CONGELATEUR<br>208 ☐/☐ PLAQUE A GAZ<br>209 ☐/☐ CUISINIÈRE<br>210 ☐/☐ RECHAUD A PETROLE / GAZ<br>211 ☐/☐ PLAQUE ELECTRIQUE<br>212 ☐/☐ PLAQUE SOLAIRE<br>213 ☐/☐ GROUPE ELECTROGENE<br>214 ☐/☐ LAMPE SOLAIRE<br><br>And 9 other symbols [3]

IDENTIFICATION MÉNAGE / ÉQUIPMENTS DU MÉNAGE

Roster: DETAILS

generated by multi-select question q200 — r200

Question	Réponse
Combien de %rostertitle% possède le ménage?<br><br>W1 self <= 10<br>M1 Ce sont beaucoup de %rostertitle%! Vous êtes sur?	NUMERIC: INTEGER — q200qty<br><br>_ _ _ _ _ _ _ _ _ _ _ _ _ _ _ _ _ _ _ _
Le ménage possède-t-il des moustiquaires?	SINGLE-SELECT — q221<br>01 ○ Oui<br>02 ○ Non
Quelle nature?<br><br>E false	MULTI-SELECT — q221nature<br>01 ☐ Imprégné<br>02 ☐ Non Imprégné<br>03 ☐ Ne sait pas
Nombre des moustiquaires actuellement utilisés?<br><br>E false	NUMERIC: INTEGER — q221quantite<br><br>_ _ _ _ _ _ _ _ _ _ _ _ _ _ _ _ _ _ _ _
Combien des moustiquaires non-imprégné possède le ménage?<br><br>E q221==1	NUMERIC: INTEGER — nbMoustiqNon<br><br>_ _ _ _ _ _ _ _ _ _ _ _ _ _ _ _ _ _ _ _
Combien des moustiquaires imprégné possède le ménage?<br><br>E q221==1	NUMERIC: INTEGER — nbMoustiqImp<br><br>_ _ _ _ _ _ _ _ _ _ _ _ _ _ _ _ _ _ _ _

Combien des moustiquaires non-classifiées possède le ménage?<br>E `q221==1`<br>V1 `nbMoustiqImp+nbMoustiqNon+nbMoustiqNonClass>0`<br>M1 Mais vous avez dit que le ménage possède des moustiquaires!	NUMERIC: INTEGER `nbMoustiqNonClass`<br>_ _ _ _ _ _ _ _ _ _ _ _ _ _ _ _ _ _ _ _

IDENTIFICATION MÉNAGE

ANIMAUX

E `continuation!=true && !$P1P2`

STATIC TEXT

Demandez au <u>chef de ménage (ou à son représentant)</u> uniquement, si son ménage possède les animaux énoncés dans la grille ci-dessous.

Le ménage possède–t-il l'un des animaux énoncés?<br>I Volailles Moutons Chèvres Bœufs Anes Porcs Chevaux	SINGLE-SELECT `animaux`<br>01 ○ Oui<br>02 ○ Non
Nombre des Volailles<br>E `animaux==1`<br>W1 `self<1000`<br>M1 Doit être entre 0 e 999	NUMERIC: INTEGER `nbVolailles`<br>_ _ _ _ _ _ _ _ _ _ _ _ _ _ _ _ _ _ _ _<br>SPECIAL VALUES<br>00 Néant
Nombre des Moutons<br>E `animaux==1`<br>W1 `self<1000`<br>M1 Doit être entre 0 e 999	NUMERIC: INTEGER `nbMoutons`<br>_ _ _ _ _ _ _ _ _ _ _ _ _ _ _ _ _ _ _ _<br>SPECIAL VALUES<br>00 Néant
Nombre des Chèvres<br>E `animaux==1`<br>W1 `self<1000`<br>M1 Doit être entre 0 e 999	NUMERIC: INTEGER `nbChevres`<br>_ _ _ _ _ _ _ _ _ _ _ _ _ _ _ _ _ _ _ _<br>SPECIAL VALUES<br>00 Néant
Nombre des Bœufs<br>E `animaux==1`<br>W1 `self<1000`<br>M1 Doit être entre 0 e 999	NUMERIC: INTEGER `nbBoeufs`<br>_ _ _ _ _ _ _ _ _ _ _ _ _ _ _ _ _ _ _ _<br>SPECIAL VALUES<br>00 Néant
Nombre des Anes<br>E `animaux==1`<br>W1 `self<1000`<br>M1 Doit être entre 0 e 999	NUMERIC: INTEGER `nbAnes`<br>_ _ _ _ _ _ _ _ _ _ _ _ _ _ _ _ _ _ _ _<br>SPECIAL VALUES<br>00 Néant

Nombre des Porcs

NUMERIC: INTEGER — `nbPorcs`

E `animaux==1`

W1 `self<1000`

M1 Doit être entre 0 e 999

SPECIAL VALUES

00	Néant

Nombre des Chevaux

NUMERIC: INTEGER — `nbChevaux`

E `animaux==1`

W1 `self<1000`

M1 Doit être entre 0 e 999

V2 `nbVolailles+nbMoutons+nbChevres+nbBoeufs+nbAnes+nbPorcs+nbChevaux > 0`

M2 Vous n'avez pas indiqué un seul animal.
Mais vous avez dit que vous avez des animaux [ici](animaux)

SPECIAL VALUES

00	Néant

Enquêteur: Qui a répondu ces sections?

SINGLE-SELECT: LINKED — `repondantBiensMenage`

SOURCE OF CATEGORIES: ROSTER MEMBRE

E `continuation!=true`

VARIABLE

LONG — `totalNewBorns`

`membre.Count(x => x.repondantNaissance != null)`

MEMBRES DU MÉNAGE

E `$OK1 || $allDead || $allLeft`

STATIC TEXT

E `bac == "1" &&false`

Ce ménage a rapporté un nouveau-né après le census!
N'oubliez pas de l'ajouter !!!

Entrez le prénom de chaque membre du ménage<br>(même s'ils sont décédés ou parti après %dateBasePretty%!)<br><br>I Commencer avec le Chef de Ménage!	LIST — `membres`

MEMBRES DU MÉNAGE

Roster: DÉTAILS

generated by list question `membres` — `membre`

Juok name of %membre%<br>E `$country==2`	TEXT — `juok_name`
Nom de %membre%	TEXT — `nom`
Have the data for this member been preloaded ?<br>(will be hidden!)	SINGLE-SELECT<br>SCOPE: HIDDEN — `preloadedMember`<br>01 ○ Yes<br>02 ○ No
ID membre	TEXT<br>SCOPE: HIDDEN — `preloadedMemberId`
newID from census (for identifying members without id, unknown before the census)	TEXT<br>SCOPE: HIDDEN — `censusID`
VARIABLE<br>`idMenage + ((@rowindex<9)?"00": ((@rowindex<99)?"0":"")) + (@rowindex+1)`	STRING — `id_membre`
VARIABLE<br>`@rowindex+1`	LONG — `rang`
Rang enregistré	NUMERIC: INTEGER<br>SCOPE: HIDDEN — `preloadedRang`

STATIC TEXT

E `false`

ID membre: %id_membre%

STATIC TEXT

E `confirmCHM==2`

Selectionnez le lien avec le nouveau Chef de Ménage!

Question	Réponse
Lien de parenté avec CM<br>V1 `/* $HDSS ? (@rowcode>1 ? membre.Count(x => x.parente == 1546) == 1 : true) : true */ membre.Count(x => x.parente == 1546) < 2`<br>M1 Il peut avoir seulement 1 Chef!<br>V2 `/* in a survey the 1st member may not be the CHM! */ $HDSS && confirmCHM == 1 ? (@rowcode == 1 ? self == 1546 : true) : true`<br>M2 Le premier faut être toujours le CHEF DE MÉNAGE !	SINGLE-SELECT `parente`<br>01546 O Chef de ménage<br>03108 O Epouse/époux<br>01720 O Coépouse<br>03599 O Fils/fille<br>03042 O Enfant adopté/confié<br>03826 O Frère/sœur<br>10157 O Père /mère<br>10275 O Petit Fils/fille<br>08913 O Neveu/nièce<br>09804 O Oncle/tante<br>03047 O Enfant conjoint(e)<br>04446 O Grand-père/grand-mère<br>04173 O Gendre/belle fille<br>00823 O Beau-frère/belle sœur<br>00539 O Autre parent<br>00531 O Aucun lien<br>And 1 other symbols [4]
Statut de résidence	SINGLE-SELECT `residence`<br>01 O Résident présent<br>02 O Résident absent<br>03 O Résident déménagé<br>04 O Résident émigré<br>05 O Résident décédé
Avant ou après la visite antérieure (%dateBasePretty%)?<br>E `residence.InList(3,4,5)`	SINGLE-SELECT `quitteQuand`<br>01 O Avant<br>02 O Après

STATIC TEXT

E `/* residence>2 */ quitteQuand == 1`

C'est pas necessaire de répondre d'autres questions sur %rostertitle%!

Question	Réponse
Sexe<br>E `$expectedPresent`	SINGLE-SELECT `sexe`<br>01 O Masculin<br>02 O Féminin

STATIC TEXT

E `$expectedPresent`

Date de Naissance
Demandez de voir un document!

Question	Réponse
Date naissance<br>E `false`	DATE `ddn`<br>..

Date de Naissance - Année

NUMERIC: INTEGER — anneeN

E `$expectedPresent`
V1 `self.InRange(1900, dateEntree.Value.Year) || self==9999`
M1 L'année doit être entre 1900 et la date d'aujourd'hui

SPECIAL VALUES

9999	Ne sait pas

Date de Naissance - Mois

SINGLE-SELECT — moisN

E `anneeN!=9999 && $expectedPresent`

01	Janvier
02	Février
03	Mars
04	Avril
05	Mai
06	Juin
07	Juillet
08	Août
09	Septembre
10	Octobre
11	Novembre
12	Décembre
99	Ne sait pas

Date de Naissance - Jour

SINGLE-SELECT — jourN

F (@optioncode==30 && moisN!=2) || (@optioncode==31 && moisN.InList(1,3,5,7,8,10,12)) || @optioncode.InRange(1,29) || @optioncode==99
E `moisN.InRange(1,12)`

01	1
02	2
03	3
04	4
05	5
06	6
07	7
08	8
09	9
10	10
11	11
12	12
13	13
14	14
15	15
16	16

And 16 other symbols [5]

Age de %membre% (ans):

NUMERIC: INTEGER — reportedAge

E `anneeN==9999`

STATIC TEXT

E `age <= 6 && (anneeN==9999 || moisN==99 || jourN==99)`

Insistez pour voir un document!

et remplissez l'année, mois et jour!

STATIC TEXT

E `$expectedPresent`

Age = %age%

Comment avez vous validé la date de naissance de %rostertitle%?<br>E `$expectedPresent`	SINGLE-SELECT ddnSource<br>02 ○ Avec le carnet de santé<br>03 ○ Avec le certificat de naissanc e<br>04 ○ CNIB / Passport<br>05 ○ Quelqu'un m'a dit<br>06 ○ Avec un autre document
Qui vous a renseigné?<br>E `ddnSource==5`	TEXT ddnSourceWho<br>..
Quel autre document?<br>E `ddnSource==6`	TEXT ddnSourceOther<br>..
VARIABLE<br>`FullYearsBetween(ddn, dateEntree)`	LONG ageOld
VARIABLE<br>`IsAnswered(reportedAge) ? reportedAge : ( moisN==99 ? FullYearsBetween(new DateTime((int) anneeN,7,1), dateEntree) : ( jourN==99 ? FullYearsBetween(new DateTime((int) anneeN,(int) moisN,15`<br>And 118 other symbols [1]	LONG age
VARIABLE<br>`CenturyMonthCode(dateEntree.Value.Month, dateEntree.Value.Year) - CenturyMonthCode(ddn.Value.Month, ddn.Value.Year) - ((dateEntree.Value.Day < ddn.Value.Day)?1:0)`	LONG ageInMonthsOld
VARIABLE<br>`IsAnswered(reportedAge) ? (long) reportedAge*12 : CenturyMonthCode(dateEntree.Value.Month, dateEntree.Value.Year) - // if don't know month, use June CenturyMonthCode((moisN==99?6:(int) moisN), (int) a`<br>And 113 other symbols [2]	LONG ageInMonths1

STATIC TEXT

E `false`

Age: %ageInMonths1% months

Est-ce que %membre% est la mère ou la guardienne d'un enfant vivant dans ce ménage?<br>E `sexe==2 && age>12 && $expectedPresent`	SINGLE-SELECT momGuardian<br>01 ○ Oui<br>02 ○ Non
Est-ce que %membre% %nom% a un surnom?<br>E `momGuardian==1 || age.InRange(0,4) && $expectedPresent`	SINGLE-SELECT existsNickname<br>01 ○ Oui<br>02 ○ Non
Quel est le surnom de %membre%?<br>E `existsNickname==1`	TEXT nickname<br>..

Question	Réponse
La mère de %membre% vit dans le ménage?<br>E `$expectedPresent`	SINGLE-SELECT — `mereDansMenage`<br>01 O Oui<br>02 O Non
Ethnie<br>E `$expectedPresent`	SINGLE-SELECT — `ethnie`<br>01 O Dafing/Marka<br>02 O Bwaba<br>03 O Mossi<br>04 O Samo<br>05 O Peulh<br>06 O Dioula<br>07 O Bobo<br>08 O Autre<br>99 O NSP
Quelle autre ethnie?<br>E `ethnie==8`	TEXT — `ethnie_o`<br>..
Religion<br>E `$expectedPresent`	SINGLE-SELECT — `religion`<br>01 O Musulman<br>02 O Catholique<br>03 O Protestant<br>04 O Animiste<br>06 O Sans religion<br>05 O Autre
Quelle autre religion?<br>E `religion==5`	TEXT — `religion_o`<br>..
Type d'instruction<br>E `age > 5`	SINGLE-SELECT — `instruction`<br>00 O Aucun<br>01 O Ecole Classique<br>02 O Ecole Coranique<br>03 O Alphabétisation<br>99 O Ne sais pas
Niveau d'instruction<br>E `instruction==1`	SINGLE-SELECT — `niveauEducation`<br>00 O Aucun<br>01 O Pre Scolaire<br>02 O Primaire / Post Primaire<br>03 O Secondaire<br>04 O Supérieur<br>05 O Technique<br>06 O Centre de formation non for melle<br>99 O Ne sais pas

Niveau d'instruction	SINGLE-SELECT<br>SCOPE: HIDDEN<br>prevNiveauEducation<br>00 O Aucun<br>01 O Pre Scolaire<br>02 O Primaire / Post Primaire<br>03 O Secondaire<br>04 O Supérieur<br>05 O Technique<br>06 O Centre de formation non for melle<br>99 O Ne sais pas

STATIC TEXT

E `$expectedPresent`

niveau anterieur: %prevNiveauEducation%

Dernière classe frequenté<br><br>F niveauEducation==1 ? @optioncode.InRange(1,3) : niveauEducation==2 ? @optioncode.InRange(4,13) : niveauEducation==3 ? @optioncode.InRange(14,16) : niveauEducation==4 ? @optioncode.InRange(17,25) : niv<br>And 102 other symbols [1]<br>E `niveauEducation.InRange(1,6)`<br>W1 `classe >= prevClasse`<br>M1 La dernière foi vous avez dit que avez fréquenté la classe %prevClasse % ???	SINGLE-SELECT<br>classe<br>01 O PS1<br>02 O PS2<br>03 O PS3<br>04 O PR1<br>05 O PR2<br>06 O PR3<br>07 O PR4<br>08 O PR5<br>09 O PR6<br>10 O SE1<br>11 O SE2<br>12 O SE3<br>13 O SE4<br>14 O SE5<br>15 O SE6<br>16 O SE7<br><br>And 18 other symbols [6]

Dernière classe frequenté

SINGLE-SELECT
SCOPE: HIDDEN

prevClasse

F /* niveauEducation==1 ? @optioncode.InRange(1,3) : niveauEducation==2 ? @optioncode.InRange(4,13) : niveauEducation==3 ? @optioncode.InRange(14,16) : niveauEducation==4 ? @optioncode.InRange(17,25) : And 113 other symbols [2]

E `niveauEducation.InRange(1,6)`

01	PS1
02	PS2
03	PS3
04	PR1
05	PR2
06	PR3
07	PR4
08	PR5
09	PR6
10	SE1
11	SE2
12	SE3
13	SE4
14	SE5
15	SE6
16	SE7

And 18 other symbols [7]

STATIC TEXT

E `$expectedPresent`

classe anterieur: %prevClasse%

Avez-vous une occupation actuellement?

SINGLE-SELECT

statutOccupation

I Une activité qui rapporte de l’argent - durant les derniers 7 jours.

E `age >= 15`

01	Occupé
02	Chômeurs
03	En quête d’emploi
04	Ménagère
05	Etudiant / Elève
06	Retraité
07	Rentier
08	Invalide
99	Autre

Quelle autre statut d'occupation?

TEXT

statutOccupationAutre

E `statutOccupation==99`

....

Occupation principale<br><br>E age > 14 && !statutOccupation.InList(2,3,4,6,8)	SINGLE-SELECT: COMBO BOX — occupation<br>01 ○ Agriculture<br>02 ○ Apiculture<br>03 ○ Berger<br>04 ○ Boucher<br>05 ○ Cathéchiste<br>06 ○ Chauffeur<br>07 ○ Coiffeur<br>08 ○ Commerce<br>09 ○ Cordonnier<br>10 ○ Cuisinier<br>11 ○ Dolotière<br>12 ○ Elève/École r/Etudiant<br>13 ○ Elevage<br>14 ○ Forgeron/ FGR=FOR<br>15 ○ Fileuse<br>16 ○ Fonc. public<br><br>And 59 other symbols [8]
Quelle autre occupation?<br><br>E occupation==96	TEXT — occupation_o<br><br>..
Comment a été l'état de santé de %membre% d urant les 12 derniers mois ?<br><br>I S'il a répondu bien: est ce que c'est très bon, bon ou plutôt moyen ? S'il a répondu mauvais: est ce que c'est très mauvais, mauvais ou plutôt m oyen ?<br>E $expectedPresent	SINGLE-SELECT — etatSante<br>01 ○ Très bon<br>02 ○ Bon<br>03 ○ Moyen<br>04 ○ Mauvais<br>05 ○ Très mauvais
A %membre% une maladie chronique, c'est-à-dire une maladie qui dure depuis plus d e trois mois ?<br><br>E $expectedPresent	SINGLE-SELECT — maladieChronique<br>01 ○ Oui<br>02 ○ Non
En dehors de cette (ces) maladie(s) chronique(s), a %membre% souffert d'une autre maladie d ans le mois passé?<br><br>E $expectedPresent	SINGLE-SELECT — autreMaladie<br>01 ○ Oui<br>02 ○ Non
Dans le mois passé, a %membre% consulté dan s un CSPS du district ou au CMA de Nouna?<br><br>E $expectedPresent	SINGLE-SELECT — consulteCentre<br>01 ○ Oui<br>02 ○ Non

Statut matrimoniale<br>E (sexe==2 && age>11) || (sexe==1 && age>14)	SINGLE-SELECT matrimon<br>01 ○ Célibataire<br>02 ○ Marié(e) Monogame<br>03 ○ Marié(e) Polygame<br>04 ○ Union Libre<br>05 ○ Divorcé(e) / Séparé(e)<br>06 ○ Veuf / Veuve
Voudriez-vous nous communiquer votre N° de Tel Personnel?<br>E age >= 10	SINGLE-SELECT givesPhone<br>001 ○ Oui<br>002 ○ Non<br>997 ○ Ne possède pas de téléphon e
Qu'est-ce que le n° de téléphone?<br>E givesPhone == 1	TEXT phoneMembre<br>..
Consentirez-vous à ce que le CRSN vous appell e par téléphone ou via le tél. d'une personne d u ménage pour des études de ce genre?<br>E age >= 10	SINGLE-SELECT consentPhone<br>01 ○ Oui<br>02 ○ Non
Quel type de téléphone possède?<br>E age >= 10 && givesPhone!=997	SINGLE-SELECT typePhone<br>001 ○ Tel. Simple<br>002 ○ Smartphone<br>999 ○ Ne sait pas<br>996 ○ Refus

STATIC TEXT

E typePhone==999

Demander à voir le téléphone s'il est à coté

Grossesse en cours<br>E sexe==2 && $childbearingAge	SINGLE-SELECT grossesse<br>01 ○ Oui<br>02 ○ Non<br>99 ○ NSP

MEMBRES DU MÉNAGE / DÉTAILS

GROSSESSE %ROSTERTITLE%

E grossesse==1

Nom du répondant<br>I (Si la personne n'est pas dans la liste, retournez a la [liste des membre s](membres) e ajoutez cette personne!)	SINGLE-SELECT: LINKED repondantGrossesse<br>SOURCE OF CATEGORIES: ROSTER MEMBRE

**Combien de mois?**<br>E grossesse == 1<br>V1 `self.InRange(1,9) || self==-1`<br>M1 Doit être enceinte entre 1 et 9 mois!	NUMERIC: INTEGER — `pregnancyMonths`<br>SPECIAL VALUES<br>-01 Ne sait pas
**Année début grossesse**<br>V1 `self.InRange(dateEntree.Value.Year-1, dateEntree.Value.Year) || self==9999`<br>M1 L'année doit être cette année ou l'anterieur.	NUMERIC: INTEGER — `anneeG`<br>SPECIAL VALUES<br>9999 Ne sait pas
**Mois début grossesse**<br>E `anneeN!=9999`<br>V1 `pregnancyMonths==-1 ? true : minMonthsSinceDebut.InRange(pregnancyMonths-1, pregnancyMonths+1)`<br>M1 Ça fait %minMonthsSinceDebut% mois - mais vous avez dit qu'ils sont %pregnancyMonths% mois!<br>V2 `pregnancyMonths==-1 ? monthsSinceDebut >= pregnancyMonths + 1 : true`<br>M2 Si c'était en %anneeG%, vous ne pouvez pas être enceinte %pregnancyMonths% mois!<br>V3 `pregnancyMonths==-1 ? minMonthsSinceDebut <= 9 : true`<br>M3 Improbable! Vous serait enceinte du moins %minMonthsSinceDebut% mois maintenant!<br>V4 `self == 99 ? true : monthsSinceDebut >= 0`<br>M4 Ne peut pas être dans l'avenir!	SINGLE-SELECT — `moisG`<br>01 ○ Janvier<br>02 ○ Février<br>03 ○ Mars<br>04 ○ Avril<br>05 ○ Mai<br>06 ○ Juin<br>07 ○ Juillet<br>08 ○ Août<br>09 ○ Septembre<br>10 ○ Octobre<br>11 ○ Novembre<br>12 ○ Décembre<br>99 ○ Ne sait pas
VARIABLE<br>`CenturyMonthCode(dateEntree.Value.Month, dateEntree.Value.Year) - CenturyMonthCode((moisG==99?1:(int) moisG), anneeG)`	LONG — `monthsSinceDebut`
VARIABLE<br>`CenturyMonthCode(dateEntree.Value.Month, dateEntree.Value.Year) - CenturyMonthCode((moisG==99?12:(int) moisG), anneeG)`	LONG — `minMonthsSinceDebut`
**Rang de la grossesse???**<br>V1 `self.InRange(1,99)`<br>M1 Doit être entre 1 et 99.	NUMERIC: INTEGER — `rangGrossesse`
**Statut**<br>E `false /* c'est grossesse en cours! */`	SINGLE-SELECT — `statutGrossesse`<br>01 ○ Accouchement intervenu<br>00 ○ Pas encore
**Date d'accouchement**<br>E `statutGrossesse==1`	DATE — `dateAccouchement`
**Months of previously reported Grossesse en cours**	NUMERIC: INTEGER<br>SCOPE: HIDDEN — `openPregnancy`

Depuis de %dateBasePretty% avez vous eu une ou plusieurs grossesses qui sont terminées?<br>E `sexe==2 && $childbearingAge`	SINGLE-SELECT `grossessesHistoriques`<br>01 ○ Oui<br>02 ○ Non
Combien de grossesses?<br>E `grossessesHistoriques==1`	NUMERIC: INTEGER `nombreGrossHist`<br>_ _

MEMBRES DU MÉNAGE / DÉTAILS

Roster: GROSSESSE

generated by numeric question `nombreGrossHist` `r_issueGrossesse`

MEMBRES DU MÉNAGE / DÉTAILS / GROSSESSE

ISSUE GROSSESSE

STATIC TEXT

E `@rowcode == 1 && IsAnswered(openPregnancy)`

%dateBasePretty% vous aviez une grossesse de %openPregnancy% mois.

Quelle était la issue de la grossesse?	SINGLE-SELECT `issueGrossesse`<br>01 ○ Accouchement<br>02 ○ Avortement<br>03 ○ Mort-Né
Date de l'issue de la grossesse	DATE `dateIssue`<br>..
C'était sa première grossesse?	SINGLE-SELECT `premiereGrossesse`<br>01 ○ Oui<br>02 ○ Non
%membre% a survie?	SINGLE-SELECT `mereSurvie`<br>01 ○ Oui<br>02 ○ Non
L'enfant a survie?	SINGLE-SELECT `enfantSurvie`<br>01 ○ Oui<br>02 ○ Non
Type d’accouchement<br>E `issueGrossesse.InList(1,3)`	SINGLE-SELECT `typeAccouchement`<br>01 ○ Voie basse<br>02 ○ Césarienne
Type d’avortement<br>E `issueGrossesse==2`	SINGLE-SELECT `typeAvortement`<br>01 ○ spontané<br>02 ○ provoqué<br>03 ○ ne sait pas / ne veut pas rep ondre

Lieu	SINGLE-SELECT lieuAccouchement
	01 ○ F. Sanitaire<br>02 ○ Acc. Villagoise<br>03 ○ Domicile / Route

Quel type de F. Sanitaire?<br>E `lieuAccouchement==1`	SINGLE-SELECT typeFsanitaire
	01 ○ CSPS<br>02 ○ CM<br>03 ○ CMA<br>04 ○ Hôpital<br>99 ○ Autre

MEMBRES DU MÉNAGE / DÉTAILS / GROSSESSE / ISSUE GROSSESSE

%LIEUACCOUCHEMENT%

E `IsAnswered(lieuAccouchement)`

A quel village?<br>I Tapez partie du nom du village, et sélectionnez de la liste	SINGLE-SELECT: COMBO BOX villAccouchement
	02 ○ Toni<br>05 ○ Boron<br>08 ○ Cissé<br>09 ○ Dankoumana<br>10 ○ Dembeléla<br>11 ○ Denissa<br>12 ○ Denissa Mossi<br>14 ○ Dionkongo<br>15 ○ Dina<br>16 ○ Dokoura<br>17 ○ Goni<br>18 ○ Kamadena<br>19 ○ Kèmena<br>21 ○ Koro<br>23 ○ Leï<br>27 ○ Ouetté<br><br>And 43 other symbols [10]

VARIABLE<br>`(CenturyMonthCode(dateEntree.Value.Month, dateEntree.Value.Year) - CenturyMonthCode(dateBase.Value.Month, dateBase.Value.Year) - ((dateEntree.Value.Day < dateBase.Value.Day)?1:0))`	LONG timeSinceLast

STATIC TEXT

E `false`

time since last census %timeSinceLast% months

MEMBRES DU MÉNAGE / DÉTAILS

NOUVEAU-NÉ %MEMBRE%

E `/* only if born after previous census */ ageInMonths1 <= (CenturyMonthCode(dateEntree.Value.Month, dateEntree.Value.`

Year) - CenturyMonthCode(dateBase.Value.Month, dateBase.Value.Year) - ((dateEntree.V And 36 other symbols [1]

Nom du répondant

I (Si la personne n'est pas dans la liste, retournez a la [liste des membre s](membres) e ajoutez cette personne!)

SINGLE-SELECT: LINKED
SOURCE OF CATEGORIES: ROSTER MEMBRE

`repondantNaissance`

Qui est la mère de %membre%

F sexe==2 && age >= 9 && (age > @current.age + 9)
E `mereDansMenage == 1`

SINGLE-SELECT: LINKED
SOURCE OF CATEGORIES: ROSTER MEMBRE

`mereDuNouveauNe`

Prenom de la mére de %rostertitle%

E `mereDansMenage == 2`

TEXT

`prenomMere`

Nom de la mére de %rostertitle%

E `mereDansMenage == 2`

TEXT

`nomMere`

Est-ce la première naissance vivante de %mere DuNouveauNe%?

SINGLE-SELECT

`premiereNaissance`

01 ○ Oui
02 ○ Non

Rang de naissance

E `premiereNaissance==2`

NUMERIC: INTEGER

`rangNaissance`

Accouchement Multiple?

SINGLE-SELECT

`accouchementMultiple`

01 ○ Oui
02 ○ Non

Combien d'enfants sont nés à cette occasion?

E `accouchementMultiple==1`
W1 `self < 6`
M1 %enfantsMultiples% enfants sont nés ensembles ?!

NUMERIC: INTEGER

`enfantsMultiples`

Combien de naissances vivantes %mereDuNou veauNe% a eu, y compris celle-ci ?

E `premiereNaissance==2`
W1 `self <= 15`
M1 Vous êtes sûr que vous avez eu %naissancesVivantes% naissances viv antes?!

NUMERIC: INTEGER

`naissancesVivantes`

Lieu de la naissance

SINGLE-SELECT

`lieuNaissance`

01 ○ Domicile
02 ○ Accoucheuses villageoises
03 ○ CSPS
04 ○ CMA
99 ○ Autre

Quel autre lieu?

E `lieuNaissance==99`

TEXT

`autreLieuNaissance`

Le père de %membre% vit dans le ménage?

SINGLE-SELECT

`pereDansMenage`

01 ○ Oui
02 ○ Non

Qui est le père de %membre%?<br>F sexe==1 && age >= 9 && (age > @current.age + 9)<br>E pereDansMenage==1	SINGLE-SELECT: LINKED<br>SOURCE OF CATEGORIES: ROSTER MEMBRE<br>pereDuNouveauNe
Prénom du père<br>E pereDansMenage==2	TEXT<br>prenomPere
Nom du père<br>E pereDansMenage==2	TEXT<br>nomPere
Ou reside le père?<br>E pereDansMenage==2	SINGLE-SELECT<br>residPere<br>01 Intérieur SSDS<br>02 Au Burkina Faso<br>03 Hors Burkina Faso
A quel village?<br>I Tapez partie du nom du village, et sélectionnez de la liste<br>E residPere==1	SINGLE-SELECT: COMBO BOX<br>residPereVill<br>02 Toni<br>05 Boron<br>08 Cissé<br>09 Dankoumana<br>10 Dembeléla<br>11 Denissa<br>12 Denissa Mossi<br>14 Dionkongo<br>15 Dina<br>16 Dokoura<br>17 Goni<br>18 Kamadena<br>19 Kèmena<br>21 Koro<br>23 Leï<br>27 Ouetté<br>And 43 other symbols [9]

A quel lieu?<br>F (@optioncode<=4 || @optioncode==99) && residPere==2 || @optioncode>4 && residPere==3<br>E `residPere>1`	SINGLE-SELECT `residPereHorsSDSS`<br>01 ○ Ouaga<br>02 ○ Bobo<br>03 ○ Dedougou<br>04 ○ Nouna<br>05 ○ Côte d'Ivoire<br>06 ○ Mali<br>07 ○ Autre lieu en Afrique<br>08 ○ Amerique<br>09 ○ Asie<br>10 ○ Europe<br>11 ○ Australie<br>99 ○ Autre
Nom du chef de ménage du père<br>E `residPere==1`	TEXT `pereNomCHM`<br>..
Est-ce que %membre% possède une moustiquaire?<br>E `$expectedPresent`	SINGLE-SELECT `moustiquaire`<br>01 ○ Non<br>02 ○ Oui, moustiquaire non imprégnée<br>03 ○ Oui, moustiquaire imprégnée d'insecticide<br>04 ○ Oui, je ne sais pas
%membre% a dormi sous moustiquaire la nuit dernière?<br>E `$expectedPresent`	SINGLE-SELECT `dormi_moustiq`<br>01 ○ Non<br>02 ○ Oui, moustiquaire non imprégnée<br>03 ○ Oui, moustiquaire imprégnée d'insecticide<br>04 ○ Oui, je ne sais pas
VARIABLE<br>`membre.Count(x => x.ageInMonths1<60)`	LONG `NumChildren_under_5`

MEMBRES DU MÉNAGE

NOTE

E `(NumChildren_under_5 == 0) && $P1P2`

STATIC TEXT

E `$P1P2 & NumChildren_under_5 == 0`

Il n y a pas des enfants moins de 5 années!
Fermez cette interview et choisissez une
MÉNAGE DE REMPLACEMENT !

MEMBRES DU MÉNAGE

Roster: MÈRE DE - %ROSTERTITLE%

generated by list question membres

child

E (mereDansMenage==1 && !IsAnswered(mereDuNouveauNe)) || (mereDansMenage==2 && ageInMonths1<60)

Qui est la mère de %membre%?<br>I (Si la personne n'est pas dans la liste, retournez a la [liste des membre s](membres) e ajoutez cette personne!)<br>F sexe==2 && age >= 12<br>E mereDansMenage == 1	SINGLE-SELECT: LINKED<br>SOURCE OF CATEGORIES: ROSTER MEMBRE<br>mere
VARIABLE<br>mere[0]	LONG<br>motherid
Qui s'occupe de %membre%?<br>I (Si la personne n'est pas dans la liste, retournez a la [liste des membre s](membres) e ajoutez cette personne!)<br>F age>=12<br>E mereDansMenage==2 && ageInMonths1<60	SINGLE-SELECT: LINKED<br>SOURCE OF CATEGORIES: ROSTER MEMBRE<br>guardian
VARIABLE<br>guardian[0]	LONG<br>guardianid

STATIC TEXT

E false

mother: %motherid% - guardian: %guardianid%

MEMBRES DU MÉNAGE

Roster: (-- COMMENTED --) ENFANT

generated by list question membres

R_child

E age.InRange(0,4) && false

Est-ce que %R_child% %nom% a un surnom?	SINGLE-SELECT<br>existschildNickname<br>01 Oui<br>02 Non
Quel est le surnom de %R_child%?<br>E existschildNickname==1	TEXT<br>childNickname
VARIABLE<br>dateEntree.Value.Day - ddn.Value.Day	LONG<br>deltaDays
VARIABLE<br>(deltaDays<0)?ageInMonths1 -1:ageInMonths1	LONG<br>adjusted

STATIC TEXT

E false

Age in completed months: %ageInMonths1%

STATIC TEXT

E !$treatmentAge && !$newBorn

%R_child% is not under 5 years old!

Comment avez vous validé la date de naissance de %R_child%?<br>E `($treatmentAge || $newBorn) && false`	SINGLE-SELECT dobSource<br>01 ○ Avec le carnet de santé<br>02 ○ Avec le certificat de naissance<br>03 ○ Quelqu'un m'a dit<br>04 ○ Avec un autre document
Qui vous a renseigné?<br>E `dobSource==3`	TEXT dobSourceWho<br>..
Quel autre document?<br>E `dobSource==4`	TEXT dobSourceOther<br>..
What is %R_child%'s vital status?<br>E `($treatmentAge || $newBorn) && false`	SINGLE-SELECT childVital<br>01 ○ Alive - slept in the house last night<br>02 ○ Alive - but temporarily absent<br>03 ○ Dead<br>04 ○ Moved within the community<br>05 ○ Moved outside the community<br>06 ○ Unknown
When did %R_child% die?<br>E `childVital == 3`	DATE whenDied<br>..
Where did %R_child% live when %R_child% died?<br>E `childVital == 3`	TEXT whereDied<br>..
What is the name of the household head where %R_child% moved to?<br>I First and Last name of new household head<br>E `childVital==4`	TEXT movedToHoH<br>..
Which community did %R_child% move to?<br>E `childVital==5`	TEXT movedToCommunity<br>..

NUTRITION

E `$OK && $P1P2`

NUTRITION

Roster: ENFANT

generated by list question `membres` — `detailenfant`

E `ageInMonths1<60`

Question	Type	Code	Réponse	ID
Est-ce que l'enfant %detailenfant% est présent lors de l'enquête ?	SINGLE-SELECT	01	Oui	QN02
		00	Non	

STATIC TEXT

Âge de %detailenfant% est: %ageInMonths1% mois

Question	Type	Code	Réponse	ID
Observez. : L'enfant %detailenfant% a-t-il un ventre gonflé aujourd'hui?	SINGLE-SELECT	01	Oui	QN06
		00	Non	
L'enfant %detailenfant% a-t-il eu de la fièvre au cours des 4 dernières semaines ?	SINGLE-SELECT	01	Oui	QN07
		00	Non	
L'enfant %detailenfant% a-t-il eu la diarrhée au cours des 4 dernières semaines ?	SINGLE-SELECT	01	Oui	QN08
		00	Non	
Observez: %detailenfant% l'enfant a-t-il un gonflement des jambes ou des bras ? (faites un test)	SINGLE-SELECT	01	Oui	QN09
		00	Non	
L'enfant %detailenfant% a des signes de kwashiorkor ou marasmes ?	SINGLE-SELECT	01	Oui	QN10
		00	Non	
Avait-elle eu des complications pendant la grossesse ou l'accouchement de %detailenfant% ?	SINGLE-SELECT	01	Oui	QN24
		00	Non	
A quel âge de la grossesse (en mois) y a t il eut ces complications ?	SINGLE-SELECT	01	< 7 moins	QN25
E `QN24==1`		02	7-8 moins	
		03	8-9 moins	
		04	> 9 moins	
%detailenfant% Avait - il /elle eut d'autres maladies les deux (2) dernières semaines ?	SINGLE-SELECT	01	Oui	QN11
E `false`		00	Non	

Enfant issu d'accouchement unique ou multiple (gémellité) ?	SINGLE-SELECT QN28<br>01 ○ Unique<br>02 ○ jumeaux
Durée de grossesse (en mois) jusqu'à la naissance de %detailenfant%<br>V1 QN29<=11<br>M1 La durrée de la grossesse doit etre inferieur ou egale a 11 mois	NUMERIC: INTEGER QN29<br>_ _
Est-ce que la mère/tutrice de l'enfant %detailenfant% est présente lors de l'enquête ?	SINGLE-SELECT QN13<br>01 ○ Oui<br>00 ○ Non
La mère de %detailenfant% est vivante ?<br>E QN13==0	SINGLE-SELECT QN14A<br>01 ○ Oui<br>00 ○ Non
Le père de %detailenfant% est vivant ?	SINGLE-SELECT QN14B<br>01 ○ Oui<br>00 ○ Non
Est-ce que la mère / tutrice de %detailenfant% travaille aux champs ?<br>E false	SINGLE-SELECT QN22A<br>01 ○ Oui<br>00 ○ Non
Si oui, préciser (Plusieurs réponses possibles)<br>E QN22A==1	MULTI-SELECT QN22B<br>01 ☐ Préparer/Défricher le champ<br>02 ☐ semer<br>03 ☐ cultiver (enlever les herbes)<br>04 ☐ récolter
Est-ce qu'elle emmène l'enfant aux champs ?<br>E QN22A==1	SINGLE-SELECT QN23<br>01 ○ Jamais<br>02 ○ Rarement<br>03 ○ Parfois<br>04 ○ Souvent
La mère/tutrice a-t-elle donné le premier lait (kolostrum) %detailenfant% juste après la naissance ?	SINGLE-SELECT QN26<br>01 ○ Oui<br>00 ○ Non
La mère/tutrice de %detailenfant% à-t-elle commencé à lui donner des aliments solides?	SINGLE-SELECT aliment_yes_no<br>01 ○ Oui<br>00 ○ Non

Question	Type	Variable
À quel âge (en mois) de %detailenfant% la mère /tutrice a-t-elle introduit la bouillie ?<br>I âge de l'enfant (en mois)<br>V1 QN27<=ageInMonths1<br>M1 le nombre de mois doit etre inferieur ou egal a l'age en mois de l'enfa nt	NUMERIC: INTEGER<br>_ _	QN27
VARIABLE<br>QN29*4.33333	DOUBLE	QN29_semaine
À quel âge (en mois) de %detailenfant% avez co mmencé à lui donner le sein ?<br>E QN26==0<br>V1 QN30<=ageInMonths1 || QN30<=11<br>M1 l'age doit etre inferieur ou egal a celui de l'enfant	NUMERIC: INTEGER<br>_ _	QN30
Votre enfant %detailenfant% a été sevré?	SINGLE-SELECT<br>01 ○ Oui<br>00 ○ Non	sevrage_yes_no
Jusqu'à quel âge (en mois) l'enfant a été sevré c ompletement (fin allaitement maternelle)<br>E sevrage_yes_no==1<br>V1 QN31<=ageInMonths1 || QN31<=48<br>M1 l'age en mois incorrecte	NUMERIC: INTEGER<br>_ _	QN31

STATIC TEXT

La santé de %detailenfant%

Question	Type	Variable
Avez-vous emmené %detailenfant% regulairem ent au SMI ?	SINGLE-SELECT<br>01 ○ Oui<br>00 ○ Non	QN32A
Avez-vous un carnet SMI de %detailenfant% ?	SINGLE-SELECT<br>01 ○ Oui<br>00 ○ Non	QN32B
Veuillez prendre la photo du carnet<br>E QN32B==1	PICTURE	QN32B_photo
A quel âge (en mois) de %detailenfant% avez-vo us commencé à l' amené au SMI ?<br>E QN32A==1<br>V1 self <= ageInMonths1<br>M1 l'age doit etre inferieur ou egal a celui de l'enfant	NUMERIC: INTEGER<br>_ _<br>SPECIAL VALUES<br>00 Moins d'un mois	QN32C
Continuez-vous à emmener %detailenfant% re gulairement ?<br>E QN32A==1	SINGLE-SELECT<br>01 ○ Oui<br>00 ○ Non	QN32D
Donnez les raisons<br>E QN32D==0	SINGLE-SELECT<br>01 ○ Etat nutritionnel amélioré<br>02 ○ Manque de moyens<br>03 ○ Autres	QN32E

Quelle autre raisons<br>E QN32E==3	TEXT QN32E_autre<br>..
A peu près combien de fois par an l'y amenez v ous ?<br>I Nombre de fois<br>E QN32D==1	NUMERIC: INTEGER QN32F<br>- - - - - - - - - - - - - - - - - -
Avait - Il/ elle reçu des vaccins ?<br>E QN32D==1	SINGLE-SELECT QN33A<br>01 O Oui<br>00 O Non

STATIC TEXT

E QN33A==1

Montrez-moi svp la carte de vaccination (prise photo !)

Enquêteur: Combien des fotos sont necessaire s?<br>E QN33A==1	NUMERIC: INTEGER numFotosVaccin<br>- - - - - - - - - - - - - - - - - -

NUTRITION / ENFANT

Roster: FOTO CARTE DE VACCINATION

generated by numeric question numFotosVaccin

fotosVaccin

Foto %fotosVaccin%	PICTURE QN33B
Avait - Il/ elle reçu des suppléments de vitamine A ?<br>E QN33A==1	SINGLE-SELECT QN34<br>01 O Oui<br>00 O Non
Au cours des dernières 24 heures, %detailenfa nt% a-t-il mangé ?	SINGLE-SELECT QN35<br>01 O Moins que d'habitude<br>02 O autant que d'habitude<br>03 O plus que d'habitude
Votre enfant, a -t- il/ elle a déjà bénéficié d'une i ntervention alimentaire ?	SINGLE-SELECT QN36A<br>01 O Oui<br>00 O Non
Combien de fois ?<br>E QN36A==1<br>V1 QN36B<=20<br>M1 le nombre de fois semble etre elevée	NUMERIC: INTEGER QN36B<br>- - - - - - - - - - - - - - - - - -
Période d'intervention alimentaire<br>E QN36A==1	SINGLE-SELECT QN36C<br>01 O En cours<br>02 O Plus maintenant - mais dans l'année dernière<br>03 O il y a plus d'un an

Question	Réponse	Code
%detailenfant% a-t-il déjà été diagnostiqué mal nutri par un professionnel de santé?	SINGLE-SELECT<br>01 ○ Oui<br>00 ○ Non	QN37A
Quand est-ce que l’enfant a été diagnostiqué m alnutri - Année<br>E QN37A==1	NUMERIC: INTEGER<br>_ _ _ _ _ _ _ _ _ _ _ _ _ _ _ _ _ _ _ _	QN37B
Quand est-ce que l’enfant a été diagnostiqué m alnutri - Mois ?<br>E QN37A==1	SINGLE-SELECT<br>01 ○ Janvier<br>02 ○ Fevrier<br>03 ○ Mars<br>04 ○ Avril<br>05 ○ Mai<br>06 ○ Juin<br>07 ○ Juillet<br>08 ○ Aout<br>09 ○ Septembre<br>10 ○ Octobre<br>11 ○ Novembre<br>12 ○ Decembre<br>99 ○ Ne sait pas	QN37Bm
%detailenfant% mange-t-il/elle à tous les repas (03 fois par jour)?	SINGLE-SELECT<br>01 ○ Oui<br>00 ○ Non	QG06
%detailenfant% mange-t-il/elle les aliments du champ du ménage à tous les repas (03 fois par jour)?	SINGLE-SELECT<br>01 ○ Oui<br>00 ○ Non	QG07
%detailenfant% mange-t-il/elle a satiété?	SINGLE-SELECT<br>01 ○ Oui<br>00 ○ Non	QG08
%detailenfant% reçoit-il le plat familial ?	SINGLE-SELECT<br>01 ○ Oui<br>00 ○ Non	QG10A
À quel âge pour la première fois ?<br>I Age (en mois)<br>E QG10A==1<br>V1 QG10B<=ageInMonths1<br>M1 le nombre de mois doit etre inferieur ou egal a l'age de l'enfant	NUMERIC: INTEGER<br>_ _ _ _ _ _ _ _ _ _ _ _ _ _ _ _ _ _ _ _	QG10B
%detailenfant% a-t-il/elle été dans une formati on sanitaire dans les derniers 12 mois?	SINGLE-SELECT<br>01 ○ Oui<br>00 ○ Non	QG11A

Question	Réponse
Pour quelle raison ?<br>E QG11A==1	MULTI-SELECT QG11B<br>01 ☐ Consultion<br>02 ☐ Hospitalisation<br>03 ☐ probleme nutritionnel<br>04 ☐ Vaccination de routine<br>09 ☐ Autres à préciser
Autre Raisons a preciser<br>E QG11B.Contains(9)	TEXT QG11B_autre<br>..
Qui a vu l'enfant ?<br>E QG12==1	MULTI-SELECT QG13B<br>01 ☐ Agent de santé du CSPS<br>02 ☐ Agent de santé du CMA<br>03 ☐ Investigateur d'étude<br>04 ☐ Agent CREN<br>09 ☐ Autres
%detailenfant% a t-il/elle été présenté dans une formation sanitaire dans les derniers 12 mois pour raison de la malnutrition ?	SINGLE-SELECT QG12<br>01 ○ Oui<br>00 ○ Non
À quelle occasion ?<br>E QG12==1	SINGLE-SELECT QG13A<br>01 ○ lors d'une campagne<br>02 ○ lors d'une consultation<br>03 ○ lors d'une vaccination de routine<br>04 ○ lors de la participation à une étude<br>09 ○ autre (précisez)
Quelle autre occasion<br>E QG13A==9	TEXT QG13A_autre<br>..
donnez-vous à %detailenfant% les céréales de votre propre champ principalement?	SINGLE-SELECT QG16<br>01 ○ Oui<br>00 ○ Non
Avez-vous un jardin que vous cultivez vous -mêmes ?	SINGLE-SELECT QG17<br>01 ○ Oui<br>00 ○ Non

Quelles plantes/condiment/végétales cultivez-v ous<br><br>E QG17==1	MULTI-SELECT QG18<br>44 ☐ Carotte<br>45 ☐ Courgette<br>46 ☐ Citrouille/ courge<br>47 ☐ Persil<br>48 ☐ Poivron<br>49 ☐ Tomate<br>50 ☐ Aubergine<br>51 ☐ Avocat<br>52 ☐ Concombre<br>53 ☐ Gombo<br>54 ☐ Oignon<br>55 ☐ Ail<br>56 ☐ Fleur de kapokier<br>99 ☐ Autre
Quelle autre plante?<br><br>E `QG18.Contains(99)`	TEXT QG18autre<br>..
Ajoutez-vous les condiments/ herbes/ produit de votre jardin à votre cuisine ?<br><br>I (question adressée à la mère)<br>E QG17==1	SINGLE-SELECT QG19<br>01 ○ Oui<br>00 ○ Non
Quelles?<br><br>F QG18.Contains(@optioncode)<br>E QG19==1	MULTI-SELECT QG20<br>44 ☐ Carotte<br>45 ☐ Courgette<br>46 ☐ Citrouille/ courge<br>47 ☐ Persil<br>48 ☐ Poivron<br>49 ☐ Tomate<br>50 ☐ Aubergine<br>51 ☐ Avocat<br>52 ☐ Concombre<br>53 ☐ Gombo<br>54 ☐ Oignon<br>55 ☐ Ail<br>56 ☐ Fleur de kapokier<br>99 ☐ Autre
Nom du répondant<br><br>I (Si la personne n'est pas dans la liste, retournez a la [liste des membre s](membres) e ajoutez cette personne!)	SINGLE-SELECT: LINKED repondantNutrition<br>SOURCE OF CATEGORIES: ROSTER MEMBRE

INSÉCURITÉ ALIMENTAIRE

E `$OK && $P1P2`

INSÉCURITÉ ALIMENTAIRE

Roster: QUESTIONS

generated by list question `membres`

`foodInsecurity`

E `age >= 12 && ( parente == 1546 || child.Count(x => x.motherid == @rowcode)>0 || child.Count(x => x.guardianid == @rowcode)>0 ) && residence.InList(1)`

Question	Réponses
Ces quatre dernières semaines, étiez-vous préoccupé que votre ménage n’avait pas assez de nourriture?	SINGLE-SELECT `FI1`<br>01 ○ Oui<br>00 ○ Non
Avec quelle fréquence cette situation s’est-elle présentée?<br>E `FI1==1`	SINGLE-SELECT `FI1frequence`<br>01 ○ Rarement (1 ou 2 fois ces 4 dernières semaines)<br>02 ○ Parfois (3 à 10 fois ces 4 dernières semaines)<br>03 ○ Souvent (plus de 10 fois ces 4 dernières semaines)
Ces quatre dernières semaines, est-ce que vous-même ou un membre de votre ménage n’a pas pu manger les types de nourriture que vous préférez à cause d’un manque de ressources?	SINGLE-SELECT `FI2`<br>01 ○ Oui<br>00 ○ Non
Avec quelle fréquence cette situation s’est-elle présentée?<br>E `FI2==1`	SINGLE-SELECT `FI2frequence`<br>01 ○ Rarement (1 ou 2 fois ces 4 dernières semaines)<br>02 ○ Parfois (3 à 10 fois ces 4 dernières semaines)<br>03 ○ Souvent (plus de 10 fois ces 4 dernières semaines)
Ces quatre dernières semaines, est-ce que vous-même ou un membre de votre ménage a mangé une variété limitée d’aliments parce que les ressources étaient insuffisantes?	SINGLE-SELECT `FI3`<br>01 ○ Oui<br>00 ○ Non
Avec quelle fréquence cette situation s’est-elle présentée?<br>E `FI3==1`	SINGLE-SELECT `FI3frequence`<br>01 ○ Rarement (1 ou 2 fois ces 4 dernières semaines)<br>02 ○ Parfois (3 à 10 fois ces 4 dernières semaines)<br>03 ○ Souvent (plus de 10 fois ces 4 dernières semaines)

Ces quatre dernières semaines, est-ce que vous-même ou un membre de votre ménage a mangé une nourriture que vous ne souhaitiez pas manger à cause du manque de ressources pour obtenir d'autres types de nourriture?	SINGLE-SELECT FI4<br>01 Oui<br>00 Non
Avec quelle fréquence cette situation s'est-elle présentée?<br>E FI4==1	SINGLE-SELECT FI4frequence<br>01 Rarement (1 ou 2 fois ces 4 dernières semaines)<br>02 Parfois (3 à 10 fois ces 4 dernières semaines)<br>03 Souvent (plus de 10 fois ces 4 dernières semaines)
Ces quatre dernières semaines, est-ce que vous-même ou un membre de votre ménage a mangé un repas plus petit que vous n'auriez souhaité parce qu'il n'y avait pas assez à manger?	SINGLE-SELECT FI5<br>01 Oui<br>00 Non
Avec quelle fréquence cette situation s'est-elle présentée?<br>E FI5==1	SINGLE-SELECT FI5frequence<br>01 Rarement (1 ou 2 fois ces 4 dernières semaines)<br>02 Parfois (3 à 10 fois ces 4 dernières semaines)<br>03 Souvent (plus de 10 fois ces 4 dernières semaines)
Ces quatre dernières semaines, est-ce que vous-même ou un membre de votre ménage a mangé moins de repas par jour parce qu'il n'y avait pas assez de nourriture?	SINGLE-SELECT FI6<br>01 Oui<br>00 Non
Avec quelle fréquence cette situation s'est-elle présentée?<br>E FI6==1	SINGLE-SELECT FI6frequence<br>01 Rarement (1 ou 2 fois ces 4 dernières semaines)<br>02 Parfois (3 à 10 fois ces 4 dernières semaines)<br>03 Souvent (plus de 10 fois ces 4 dernières semaines)
Ces quatre dernières semaines, est-il arrivé que le ménage soit complètement sans nourriture parce qu'il n'y avait pas de ressources pour en acheter?	SINGLE-SELECT FI7<br>01 Oui<br>00 Non

Avec quelle fréquence cette situation s'est-elle présentée?<br><br>E FI7==1	SINGLE-SELECT FI7frequence<br>01 Rarement (1 ou 2 fois ces 4 dernières semaines)<br>02 Parfois (3 à 10 fois ces 4 dernières semaines)<br>03 Souvent (plus de 10 fois ces 4 dernières semaines)
Ces quatre dernières semaines, est-ce que vous-même ou un membre de votre ménage est allé au lit en ayant faim parce qu'il n'y avait pas assez de nourriture?	SINGLE-SELECT FI8<br>01 Oui<br>00 Non
Avec quelle fréquence cette situation s'est-elle présentée?<br><br>E FI8==1	SINGLE-SELECT FI8frequence<br>01 Rarement (1 ou 2 fois ces 4 dernières semaines)<br>02 Parfois (3 à 10 fois ces 4 dernières semaines)<br>03 Souvent (plus de 10 fois ces 4 dernières semaines)
Ces quatre dernières semaines, est-ce que vous-même ou un membre de votre ménage a passé toute une journée et toute une nuit sans manger parce qu'il n'y avait pas assez de nourriture?	SINGLE-SELECT FI9<br>01 Oui<br>00 Non
Avec quelle fréquence cette situation s'est-elle présentée?<br><br>E FI9==1	SINGLE-SELECT FI9frequence<br>01 Rarement (1 ou 2 fois ces 4 dernières semaines)<br>02 Parfois (3 à 10 fois ces 4 dernières semaines)<br>03 Souvent (plus de 10 fois ces 4 dernières semaines)

ANTHROPOMÉTRIE

E $OK && $P1P2

ANTHROPOMÉTRIE

Roster: ANTHROPOMÉTRIE

generated by list question membres

anthropometrie

E ageInMonths1<60

STATIC TEXT

Now I would like to ask the mother of your child some questions about the health of your child under 5 years of age and I would like to take measurements of height and weight. After that I would like to continue with the questions about the mother of your child's health and the nutrition of your child under 5.

Question	Answer	ID
Is the health card available?	SINGLE-SELECT<br>01 ○ Yes<br>02 ○ No	an02
Is the child a twin?	SINGLE-SELECT<br>01 ○ Single<br>02 ○ Twin	an05
Did the child have fever over the last 2 weeks?	SINGLE-SELECT<br>01 ○ Yes<br>02 ○ No	an06
Was the child diagnosed with Malaria over the last 2 weeks?	SINGLE-SELECT<br>01 ○ Yes<br>02 ○ No	an07
Did the child have diarrhea over the last 2 weeks?	SINGLE-SELECT<br>01 ○ Yes<br>02 ○ No	an08
Did the child have any other diseases over the last 2 weeks?	SINGLE-SELECT<br>01 ○ Yes<br>02 ○ No	an09
Did you visit the health care center or hospital over the last 2 weeks because your child was sick?	SINGLE-SELECT<br>01 ○ Yes<br>02 ○ No	an10

ANTHROPOMÉTRIE / ANTHROPOMÉTRIE

ANTHROPOMETRIC MEASURES

anthro_measure

E QN02==1

STATIC TEXT

Ask %rostertitle%'s mother if it has a health record with length and weight at birth. Write down the information below. Afterwards ask her to remove the shoes, heavy clothing and / or heavy layers from the child. The mother has to take off her shoes. Inform the mother that you will take the measurements twice one after another.

Birth length in centimeter (##) (as in the health card)<br>W1 `(self>=35 && self<=60) || self == 9999`<br>M1 Please check birth length - is this value correct?	NUMERIC: INTEGER qn_blength<br>_______________<br>SPECIAL VALUES<br>9999 Don't know
Birth weight in gram (####) (as in the health card)<br>W1 `(self>=1000 && self<=5000) || self == 9999`<br>M1 Please check birth weight - is this value correct?	NUMERIC: INTEGER qn_bweight<br>_______________<br>SPECIAL VALUES<br>9999 Don't know
Measurement<br>V1 `self == 1 ? (qn_height1<85 || ageInMonths1<=24) : true`<br>M1 L'enfant devrait être mésuré debout!<br>V2 `self == 2 ? (qn_height1>=85 || ageInMonths1>24) : true`<br>M2 L'enfant devrait être mésuré couché!	SINGLE-SELECT qn_measurement<br>01 ○ lying (younger than 2 yrs or below 85cm)<br>02 ○ standing (older than 2 yrs or above 85cm)
VARIABLE<br>`ageInMonths1<=6?6: ageInMonths1<=12?12: ageInMonths1<=24?24: ageInMonths1<=36?36: ageInMonths1<=48?48: ageInMonths1<=60?60: 60`	LONG age_bracket<br>This variable is excluded from the exported data

STATIC TEXT

E `false`

Age (months): %ageInMonths1% (age bracket: %age_bracket%)

Height1 today in centimeter (###,#)<br>W1 `// WHO growth standards height for age (0 to 60 months) by sex sexe==2 ? self.InRange(Height[(int)age_bracket].minF,Height[(int)age_bracket].maxF) : self.InRange(Height[(int)age_bracket].min` And 1056 other symbols [1]<br>M1 Please check height - is this value correct?	NUMERIC: DECIMAL qn_height1<br>_______________
Height2 today in centimeter (###,#)<br>V1 `(qn_height1-self)<=2.01 && (qn_height1-self)>=-2.01`<br>M1 The difference of both measurements is more then 2 cm - please check and correct or remeasure!	NUMERIC: DECIMAL qn_height2<br>_______________
Weight1 today in kilogram (##,#)<br>W1 `// WHO growth standards weight for age (0 to 5 years) by sex sexe==2 ? self.InRange(Weight[(int)age_bracket].minF,Weight[(int)age_bracket].maxF) : self.InRange(Weight[(int)age_bracket].minM,W` And 946 other symbols [2]<br>M1 Please check weight - is this value correct?	NUMERIC: DECIMAL qn_weight1<br>_______________
Weight2 today in kilogram (##,#)<br>V1 `(qn_weight1-self)<=0.31 && (qn_weight1-self)>=-0.31`<br>M1 The difference of both measurements is more then 300 gram - please check and correct or remeasure!	NUMERIC: DECIMAL qn_weight2<br>_______________
Do you want to enter a comment?<br>I eg if there are problems to perform the measurement, or if there are factors influencing the measurement's precision, eg physical disability of the child etc.	SINGLE-SELECT has_comment_child<br>01 ○ Yes<br>02 ○ No

Comment to child's height and weight measurement<br>E has_comment_child==1	TEXT qn_comment_child<br>........

STATIC TEXT

From the mother

Mother's Height1 today in centimeter (###,#)<br>W1 self>150 && self<190<br>M1 Please check height - is this a true value?	NUMERIC: DECIMAL qn_heightm1<br>- - - - - - -
Mother's Height2 today in centimeter (###,#)<br>W1 self>150 && self<190<br>M1 Please check height - is this a true value?<br>V2 (qn_heightm1-self)<=2.01 && (qn_heightm1-self)>=-2.01<br>M2 The difference of both measurements is more then 2 cm - please check and correct or remeasure!	NUMERIC: DECIMAL qn_heightm2<br>- - - - - - -
Mother's Weight1 today in kilogram (##,#)<br>W1 self>48 && self<90<br>M1 Please check weight - is this a true value?	NUMERIC: DECIMAL qn_weightm1<br>- - - - - - -
Mother's weight2 today in kilogram (##,#)<br>W1 self>48 && self<90<br>M1 Please check weight - is this a true value?<br>V2 (qn_weightm1-self)<=0.21 && (qn_weightm1-self)>=-0.21<br>M2 The difference of both measurements is more then 200 gram - please check and correct or remeasure!	NUMERIC: DECIMAL qn_weightm2<br>- - - - - - -
Do you want to enter a comment?<br>I eg if there are problems to perform the measurement, or if there are factors influencing the measurement's precision	SINGLE-SELECT has_comment_mother<br>01 ○ Yes<br>02 ○ No
Comment to mothers's height and weight measurement<br>E has_comment_mother==1	TEXT qn_comment_mother<br>........

REVENUS ET DÉPENSES

E `$OK1 && ($P1P2 || $randomSampleRound1)`

REVENUS ET DÉPENSES

Roster: REVENUS/DÉPENSES

generated by list question `membres` — `rmembre`

E `age>14 && residence.InList(1)`

STATIC TEXT

Production agricole pendant la campagne passée

%rmembre% a cultivé l'un des produits agricoles énoncés au cours de la campagne passée?	MULTI-SELECT: YES/NO — `produit`<br>01 ☐/☐ Arachides et sous produits<br>02 ☐/☐ Calebasses (entières<br>03 ☐/☐ Chou<br>04 ☐/☐ Coton et sous produits<br>05 ☐/☐ Fonio<br>06 ☐/☐ Cultures fruitières<br>07 ☐/☐ Igname<br>08 ☐/☐ Mais et sous produits<br>09 ☐/☐ Mil/ sorgho et sous produits<br>10 ☐/☐ Niébé et sous produits<br>11 ☐/☐ Oignon<br>12 ☐/☐ Oseille et sous produits<br>13 ☐/☐ Pastèque<br>14 ☐/☐ Pomme de terre<br>15 ☐/☐ Pois de terre et sous produits<br>16 ☐/☐ Riz et sous produits<br><br>And 9 other symbols [11]

REVENUS ET DÉPENSES / REVENUS/DÉPENSES

Roster: CULTIVÉ

generated by multi-select question `produit` — `rproduit`

quel est la Quantité de %rproduit% produit?<br><br>I veuillez noter la quantité et continuer pour selectionner l'unité	NUMERIC: INTEGER — `qtiteproduit`<br><br>_ _

Quel est l'unité de mesure de %rproduit%	SINGLE-SELECT uniteMesure<br>01 ○ Boîte<br>02 ○ Tine<br>03 ○ Sac<br>04 ○ Kilogramme<br>05 ○ Nombre<br>06 ○ Tonne

STATIC TEXT

Revenu monétaire par vente de produits agropastoraux

%rmembre% a vendu des produits agropastor aux énoncés au cours du mois passé et des 5 mois précédent le mois passé.?	MULTI-SELECT: YES/NO produitVendu<br>01 ☐/☐ Arachides et sous prod uits<br>02 ☐/☐ Calebasses (par moitié)<br>03 ☐/☐ Chou<br>04 ☐/☐ Coton et sous produits<br>05 ☐/☐ Fonio<br>06 ☐/☐ Cultures fruitières<br>07 ☐/☐ Igname<br>08 ☐/☐ Mais et sous produits<br>09 ☐/☐ Sorgho et sous produi ts<br>38 ☐/☐ Petit mil et sous produ its<br>10 ☐/☐ Niébé et sous produits<br>11 ☐/☐ Oignon<br>12 ☐/☐ Oseille et sous produit s<br>13 ☐/☐ Pastèque<br>14 ☐/☐ Pomme de terre<br>15 ☐/☐ Pois de terre et sous-p roduits<br>And 22 other symbols [12]

REVENUS ET DÉPENSES / REVENUS/DÉPENSES

Roster: VENDU

generated by multi-select question produitVendu rProduitVendu

Montant du %rProduitVendu% vendu le mois p assé? (CFA)	NUMERIC: INTEGER montantvenduMois<br>_ _
Montant du %rProduitVendu% vendu les 6 moi s passé? (CFA)	NUMERIC: INTEGER montantvendu5moispre<br>_ _<br>SPECIAL VALUES<br>-01 Ne sait pas

STATIC TEXT

Revenu monétaire par salaires, vente de produits NON-agropastoraux, commerce et pensions

%rmembre% a touché, dans le 6 mois passés, un revenu monétaire provenant d'un salaire, de la vente de produits NON agropastoraux, d'un commerce ou d'une pension?	SINGLE-SELECT revenuMone_yes_no<br>01 ○ Oui<br>00 ○ Non
Quelle est la source du Revenu Monetaire?<br>E revenuMone_yes_no==1	MULTI-SELECT sourceRevenu<br>01 ☐ Salaire régulier<br>02 ☐ Salaire occasionnel<br>03 ☐ Vente de produits Non agropastoraux<br>04 ☐ Commerce<br>05 ☐ Pension de retraite, Pension alimentaire, Allocations familiales<br>06 ☐ Autre sources
Autre Revenu monetaire<br>E sourceRevenu.Contains(6)	TEXT autreRevenu<br>................................

REVENUS ET DÉPENSES / REVENUS/DÉPENSES

Roster: REVENU

generated by multi-select question sourceRevenu — revenus

Montant du revenu de %rostertitle% du mois passé? (CFA)	NUMERIC: INTEGER montantRevenu<br>_ _ _ _ _ _ _ _ _ _ _ _ _ _ _ _<br>SPECIAL VALUES<br>0000000 Néant<br>9999999 Ne sait pas
Montant du revenu de %rostertitle% des 6 mois passé? (CFA)	NUMERIC: INTEGER montantRevenu5Mois<br>_ _ _ _ _ _ _ _ _ _ _ _ _ _ _ _<br>SPECIAL VALUES<br>00 Néant<br>-01 Ne sait pas

Si salaires réguliers ou pensions, dans quelle tranche se situe le salaire ?<br>E sourceRevenu.Contains(1) || sourceRevenu.Contains(5) &&false	SINGLE-SELECT trancheSalaire<br>01 ○ 20 000 CFA ou moins<br>02 ○ Entre 21 et 35 000 CFA<br>03 ○ Entre 36 et 50 000 CFA<br>04 ○ Entre 51 et 75 000 CFA<br>05 ○ Entre 76 et 125 000 CFA<br>06 ○ Entre 126 et 175 000 CFA<br>07 ○ 176 000 CFA ou plus<br>08 ○ Refuse de répondre

STATIC TEXT

%rmembre% , est-ce que un membre de votre f amille ou un ami vous a versé de l’argent, ou vo us avez pris un crédit les 6 mois passés?	SINGLE-SELECT versementArg<br>01 ○ Oui<br>00 ○ Non
Quelle est la Source provenance de ce argent?<br>E versementArg==1	MULTI-SELECT sourceProvenance<br>01 ☐ Parents dans le ménage (fils , fille, neveu, père, etc.)<br>02 ☐ Parents à l’extérieur du mén age (fils, fille, neveu, père, et c.)<br>03 ☐ Amis<br>04 ☐ Emigrés en dehors du pays (amis ou parents)<br>05 ☐ Crédit<br>06 ☐ Autre personne

REVENUS ET DÉPENSES / REVENUS/DÉPENSES

Roster: VERSEMENT

generated by multi-select question sourceProvenance versements

Montant du mois passé? (CFA)	NUMERIC: INTEGER montantMois<br>_ _<br>SPECIAL VALUES<br>00 Néant
Montant des 6 mois passé? (CFA)	NUMERIC: INTEGER montant5MoisPasse<br>_ _

Pour quoi a été utilisé cet argent?

MULTI-SELECT utilisation

- 01 ☐ Soutien à la famille pour des dépenses quotidiennes (rep as, ménage, transports quot idiens, logement)
- 02 ☐ Soutien à la famille pour des réparations occasionnelles dans la maison ou d'outils o u de moyens de transport
- 03 ☐ Fêtes, cérémonies (mariage, baptême, décès/funérailles)
- 04 ☐ Soins médicaux
- 05 ☐ Scolarisation, Education, Alp habétisation
- 06 ☐ Assurance maladie (AMU, AS MAT, ...) (inscription et/ou c otisation)
- 07 ☐ Autre destination
- 08 ☐ Néant

STATIC TEXT

Dépenses du ménage

%rmembre% avez vous dépensé de l'argent po ur l'un ou plusieurs des motifs suivants dans le s 6 mois passé?

I Lire la liste

MULTI-SELECT: YES/NO depenseMembre

- 01 ☐/☐ Loyer
- 02 ☐/☐ Electricité
- 03 ☐/☐ Téléphone
- 04 ☐/☐ Matériaux de construc tion (tôles, briques, sec cos, ...)
- 05 ☐/☐ Boissons ou eau
- 06 ☐/☐ Vivres (céréales, condi ments, ...)
- 07 ☐/☐ Ticket de transport ou Carburant
- 08 ☐/☐ Achat ou réparation d' un moyen de transpor t
- 09 ☐/☐ Scolarisation, Educatio n, Alphabétisation
- 10 ☐/☐ Vêtements
- 11 ☐/☐ Soins médicaux
- 12 ☐/☐ Assurance maladie (A MBC)
- 13 ☐/☐ Engrais, pesticide
- 14 ☐/☐ Outils agricoles ou frai s de réparation d'outils agricoles
- 15 ☐/☐ Animaux
- 16 ☐/☐ Dépenses religieuses

And 7 other symbols [13]

Roster: DETAIL

generated by multi-select question depenseMembre

rdepenseMembre

Montant de %rdepenseMembre% depensé le mois passé? (CFA)	NUMERIC: INTEGER montantDep<br>_ _<br>SPECIAL VALUES<br>0000000 Néant<br>9999999 Ne sait pas
Montant de %rdepenseMembre% depensé les 6 mois passé? (CFA)	NUMERIC: INTEGER montantDep5mois<br>_ _<br>SPECIAL VALUES<br>0000000 Néant<br>9999999 Ne sait pas

MORBIDITÉ

E `/* $OK1 && membre.Any(member => member.maladieChronique==1 | member.autreMaladie==1) && $randomSampleRound1 */ $OK1 && $randomSampleRound1`

MORBIDITÉ

Roster: MALADIES CHRONIQUES

generated by list question membres

chronique

E `maladieChronique==1`

STATIC TEXT

2.1: Inventaire des maladies chroniques et de leurs épisodes au cours des 30 derniers jours

Nom de la maladie chronique<br><br>I Ecrire NSP si %chronique% ne connait pas le nom	LIST nomMaladieChronique<br><br>..

MORBIDITÉ / MALADIES CHRONIQUES

Roster: MALADIE

generated by list question nomMaladieChronique

r_chronique

Verifiez le nom de la maladie e ecrivez le nom e n francais<br><br>I (we could select the name from the list of chronic deseases?)	TEXT maladieChroniqueVerifie<br>SCOPE: SUPERVISOR<br><br>..
VARIABLE<br>`nomMaladieChronique[@rowcode-1].ToString().Substring(5)`	STRING nomMC

MORBIDITÉ / MALADIES CHRONIQUES / MALADIE

Roster: SYMPTÔME

generated by fixed list

r_symptomes

01 Symptôme 1

02 Symptôme 2

03 Symptôme 3

04 Symptôme 4

05 Symptôme 5

06 Symptôme 6

De quels symptômes souffrez-vous à cause de votre maladie chronique?	SINGLE-SELECT: COMBO BOX	symptoms
I Marguez "NON APPLICABLE" s'il n y a plus de symptômes	00 ○ NON APPLICABLE	
	01 ○ Abcès	
	02 ○ Anémie	
	03 ○ Bouche fermée, crispée	
	04 ○ Boutons	
	05 ○ Boutons sur tout le corps	
	06 ○ Constipation	
	07 ○ Convulsions	
	08 ○ Corps chaud	
	09 ○ Corps enflé (œdème général)	
	10 ○ Coup rejeté en arrière	
	11 ○ Démangeaisons (prurit)	
	12 ○ Déshydratation	
	13 ○ Diarrhée aigue	
	14 ○ Diarrhée chronique	
	15 ○ Difficultés à respirer (dyspnée)	
	And 63 other symbols [19]	

Enqueteur: Selectionnez la maladie dans la liste

SINGLE-SELECT: COMBO BOX codeMaladieChronique

100	○ Maladies ou infections pulmonaires chroniques (poumons)
101	○ Asthme
102	○ Tuberculose
103	○ Maladies ou infections oculaires chroniques (œil)
104	○ Conjonctivite chronique
105	○ Borgne
106	○ Carence en vitamine A (avitaminose A)
107	○ Cataracte
108	○ Cécité / Aveugle
109	○ Cécité nocturne ou crépusculaire
110	○ Onchocercose (cécité des rivières)
111	○ Tension de l'œil (glaucome)
112	○ Trachome
113	○ Maladies ou infections dermatologiques chroniques (peau)
114	○ Gale
115	○ Teignes (dermatophyties)

And 51 other symbols [14]

Qui vous a dit le nom de votre maladie ?

E
```
nomMaladieChronique[@rowcode-1].ToString().ToUpper() !=
(@rowcode + " -> " + "NSP") /* don't show question for l
istentries "nsp, "NSP", etc. so it gets a N/A value auto
matically */
```

SINGLE-SELECT sourceNomChronique

01	○ Evident (par exemple dans le cas d'un accident)
02	○ Mère ou père
03	○ Personnel de santé
04	○ Autres personnes
05	○ Moi-même
06	○ Ne sait pas

VARIABLE

```
new string[] {"EVI","MPD","PDS","AUP","MOM","NSP","NAP"}
[(int) sourceNomChronique-1]
```

STRING codeSourceMC

STATIC TEXT

E `false`

%codeSourceMC%

STATIC TEXT

À quelle date a débuté votre maladie ?

Année

NUMERIC: INTEGER — anneeMC

V1 `self.InRange(1900, dateEntree.Value.Year) || self==1799`

M1 L'année doit être entre 1900 e maintenant

SPECIAL VALUES

1799	Ne sait pas

Mois

SINGLE-SELECT — moisMC

E `IsAnswered(anneeMC) && anneeMC!=1799`

01	Janvier
02	Février
03	Mars
04	Avril
05	Mai
06	Juin
07	Juillet
08	Août
09	Septembre
10	Octobre
11	Novembre
12	Décembre
99	Ne sait pas

Jour

SINGLE-SELECT — jourMC

F (@optioncode==30 && moisMC!=2) || (@optioncode==31 && moisMC .InList(1,3,5,7,8,10,12)) || @optioncode.InRange(1,29) || @optioncode ==99

E `moisMC.InRange(1,12)`

01	1
02	2
03	3
04	4
05	5
06	6
07	7
08	8
09	9
10	10
11	11
12	12
13	13
14	14
15	15
16	16

And 16 other symbols [15]

Choisissez le niveau de handicap du à votre ma ladie	SINGLE-SELECT handicapMC
	01 ○ Incapacité à réaliser les activ ités vitales (manger, faire sa toilette)
	02 ○ Incapacité à réaliser les activ ités du quotidien (préparer l es repas, confectionner des outils, garder les animaux...)
	03 ○ Incapacité à maintenir une a ctivité professionnelle ou la scolarisation (champs, atelie rs école, service...)
	04 ○ Incapacité à continuer des l oisirs (activités sportives, loi sirs...)
	05 ○ Autres limitations
	06 ○ Aucune limitation
Nom du répondant<br>I (Si la personne n'est pas dans la liste, retournez a la [liste des membre s](membres) e ajoutez cette personne!)	SINGLE-SELECT: LINKED repondantMaladies<br>SOURCE OF CATEGORIES: ROSTER MEMBRE

MORBIDITÉ

Roster: MALADIES AIGUËS

generated by list question membres aigue

E autreMaladie==1

Noms des maladies aiguës<br>I Ecrire NSP si %aigue% ne connait pas le nom	LIST nomMaladieAigue<br>..

MORBIDITÉ / MALADIES AIGUËS

Roster: MALADIE

generated by list question nomMaladieAigue r_aigue

Verifiez le nom de la maladie e ecrivez le nom e n francais<br>I ecrivez NSP si vous ne savez pas.	TEXT maladieAigueVerifie<br>SCOPE: SUPERVISOR<br>..
VARIABLE<br>nomMaladieAigue[@rowcode-1].ToString().Substring(5)	STRING nomMA

MORBIDITÉ / MALADIES AIGUËS / MALADIE

Roster: SYMPTÔME

generated by fixed list r_symptomesMA

01 1

02 2

03 3

04 4

05 5

06 6

Quels symptômes avez-vous ressenti au cours de cette maladie?<br><br>I Marguez "NON APPLICABLE" s'il n y a plus de symptômes	SINGLE-SELECT: COMBO BOX `symptomsMA`<br>00 ○ NON APPLICABLE<br>01 ○ Abcès<br>02 ○ Anémie<br>03 ○ Bouche fermée, crispée<br>04 ○ Boutons<br>05 ○ Boutons sur tout le corps<br>06 ○ Constipation<br>07 ○ Convulsions<br>08 ○ Corps chaud<br>09 ○ Corps enflé (œdème général)<br>10 ○ Coup rejeté en arrière<br>11 ○ Démangeaisons (prurit)<br>12 ○ Déshydratation<br>13 ○ Diarrhée aigue<br>14 ○ Diarrhée chronique<br>15 ○ Difficultés à respirer (dyspnée)<br><br>And 63 other symbols [20]

Selectionnez la maladie

SINGLE-SELECT: COMBO BOX — codeMaladieAigue

200	Maladies ou infections pulmonaires aiguës (poumons)
201	Bronchiolite
202	Bronchite
203	Pneumonie
204	Maladies ou infections respiratoires hautes aiguës (gorge, nez, oreilles)
205	Coqueluche
206	Grippe
207	Infections de la gorge (laryngite, angine ...)
208	Infections de l'oreille (otites, ...)
209	Infections du nez
210	Oreillons
211	Rhume
212	Maladies ou infections oculaires aigues (œil)
213	Conjonctivite à chlamydiae appelée 'Apollo'
214	Conjonctivite aigue
215	Trachome

And 66 other symbols [16]

Qui vous a dit le nom de votre maladie ?

E
```
nomMaladieAigue[@rowcode-1].ToString().ToUpper() != (@rowcode + " -> " + "NSP") /* don't show question for liste ntries "nsp, "NSP", etc. so it gets a N/A value automatically */
```

SINGLE-SELECT — sourceNomAigue

01	Evident (par exemple dans le cas d'un accident)
02	Mère ou père
03	Personnel de santé
04	Autres personnes
05	Moi-même
06	Ne sait pas

VARIABLE

```
new string[] {"EVI","MPD","PDS","AUP","MOM","NSP","NAP"}[(int) sourceNomAigue-1]
```

STRING — codeSourceMA

STATIC TEXT

À quelle date a débuté votre maladie ?

**Année**<br>V1 `self.InRange(1900, dateEntree.Value.Year) || self==1799`<br>M1 L'année doit être entre 1900 e maintenant	NUMERIC: INTEGER — anneeMA<br>_ _<br>SPECIAL VALUES<br>1799 Ne sait pas
**Mois**<br>E `IsAnswered(anneeMA) && anneeMA!=1799`	SINGLE-SELECT — moisMA<br>01 ○ Janvier<br>02 ○ Février<br>03 ○ Mars<br>04 ○ Avril<br>05 ○ Mai<br>06 ○ Juin<br>07 ○ Juillet<br>08 ○ Août<br>09 ○ Septembre<br>10 ○ Octobre<br>11 ○ Novembre<br>12 ○ Décembre<br>99 ○ Ne sait pas
**Jour**<br>F (@optioncode==30 && moisMA!=2) || (@optioncode==31 && moisMA .InList(1,3,5,7,8,10,12)) || @optioncode.InRange(1,29) || @optioncode ==99<br>E `moisMA.InRange(1,12)`	SINGLE-SELECT — jourMA<br>01 ○ 1<br>02 ○ 2<br>03 ○ 3<br>04 ○ 4<br>05 ○ 5<br>06 ○ 6<br>07 ○ 7<br>08 ○ 8<br>09 ○ 9<br>10 ○ 10<br>11 ○ 11<br>12 ○ 12<br>13 ○ 13<br>14 ○ 14<br>15 ○ 15<br>16 ○ 16<br><br>And 16 other symbols [17]
**Aujourd’hui, vos symptômes sont-ils terminés?**	SINGLE-SELECT — symptomsCrisesMAtermines<br>01 ○ Oui<br>02 ○ Non

STATIC TEXT

E `symptomsCrisesMAtermines==1`

À quelle date vos symptômes ont-ils cessé?

Année<br><br>E `symptomsCrisesMAtermines==1`<br>V1 `self.InRange(1900, dateEntree.Value.Year) || self==1799`<br>M1 L'année doit être entre 1900 e 2018	NUMERIC: INTEGER `anneeCrisesMACesse`<br><br>SPECIAL VALUES<br>1799 Ne sait pas
Mois<br><br>E `IsAnswered(anneeCrisesMACesse) && anneeCrisesMACesse!=1799`	SINGLE-SELECT `moisCrisesMACesse`<br>01 ○ Janvier<br>02 ○ Février<br>03 ○ Mars<br>04 ○ Avril<br>05 ○ Mai<br>06 ○ Juin<br>07 ○ Juillet<br>08 ○ Août<br>09 ○ Septembre<br>10 ○ Octobre<br>11 ○ Novembre<br>12 ○ Décembre<br>99 ○ Ne sait pas
Jour<br><br>F (@optioncode==30 && moisCrisesMACesse!=2) || (@optioncode==31 && moisCrisesMACesse.InList(1,3,5,7,8,10,12)) || @optioncode.InRange(1,29) || @optioncode==99<br>E `moisCrisesMACesse.InRange(1,12)`	SINGLE-SELECT `jourCrisesMACesse`<br>01 ○ 1<br>02 ○ 2<br>03 ○ 3<br>04 ○ 4<br>05 ○ 5<br>06 ○ 6<br>07 ○ 7<br>08 ○ 8<br>09 ○ 9<br>10 ○ 10<br>11 ○ 11<br>12 ○ 12<br>13 ○ 13<br>14 ○ 14<br>15 ○ 15<br>16 ○ 16<br><br>And 16 other symbols [18]
Qui a répondu ces questions?	SINGLE-SELECT: LINKED<br>SOURCE OF CATEGORIES: ROSTER MEMBRE `repondantMaigues`

MORBIDITÉ

Roster: SÉVÉRITÉ DU PROBLÈME DE SANTÉ

generated by list question membres

r_soins

E (maladieChronique==1) || autreMaladie==1

VARIABLE<br>@rowcode	LONG ixMembre
Lorsque vous étiez malade, avez-vous cru que vous alliez mourir?<br>E /* (bool) termineesA */ true	SINGLE-SELECT peurMourirA<br>01 Oui<br>02 Non
Choisissez parmi les propositions suivantes le degré de sévérité de votre maladie	SINGLE-SELECT severiteMA<br>06 Incapacité à réaliser les activités vitales (manger, téter pour les enfants en bas âge, faire sa toilette)<br>05 Incapacité à réaliser les activités du quotidien (préparer les repas, confectionner des outils, garder les animaux…)<br>04 Incapacité à maintenir une activité professionnelle ou la scolarisation (champs, ateliers, école, service…)<br>03 Incapacité à continuer des loisirs (activités sportives, loisirs…)<br>02 Autres limitations<br>01 Aucune limitation
Votre maladie vous a-t-elle empêché de mener à bien vos activités quotidiennes ?<br>E age>=6	SINGLE-SELECT empecheActivitesMA<br>01 Oui<br>02 Non
Combien de temps?<br>E empecheActivitesMA==1<br>V1 self<40 || self.InList(99,97)<br>M1 Impossible! C'est trop de temps.	NUMERIC: INTEGER tempsEmpecheActivitesMA<br>_ _ _ _ _ _ _ _ _ _ _ _ _ _ _ _ _ _ _ _<br>SPECIAL VALUES<br>99 Ne sait pas<br>97 Non applicable
%tempsEmpecheActivitesMA% Heures ou Jours ?<br>E tempsEmpecheActivitesMA<40	SINGLE-SELECT unit3A<br>01 Heures<br>02 Jours
Votre maladie vous a t-elle empêché d'aller à l'école?<br>E age>=6 && statutOccupation==5	SINGLE-SELECT empecheEcoleMA<br>01 Oui<br>02 Non

Combien de temps?<br>E `empecheEcoleMA==1`<br>V1 `self<40 || self.InList(99,97)`<br>M1 Impossible! C'est trop de temps.	NUMERIC: INTEGER `tempsEmpecheEcoleMA`<br>_ _ _ _ _ _ _ _ _ _ _ _ _ _ _ _ _ _ _ _<br>SPECIAL VALUES<br>99 Ne sait pas<br>97 Non applicable
%tempsEmpecheEcoleMA% Heures ou Jours?<br>E `tempsEmpecheEcoleMA<40`	SINGLE-SELECT `unit4A`<br>01 ○ Heures<br>02 ○ Jours
Qui a répondu ces questions?<br>I (Si la personne n'est pas dans la liste, retournez a la [liste des membre s](membres) e ajoutez cette personne!)	SINGLE-SELECT: LINKED `repondantSoins`<br>SOURCE OF CATEGORIES: ROSTER MEMBRE

MORBIDITÉ

Roster: EXAMEN PHYSIQUE - %ROSTERTITLE%

generated by list question `membres` — `examenPhysique`

E `residence.InList(1,2)`

MORBIDITÉ / EXAMEN PHYSIQUE - %ROSTERTITLE%

Roster: ANTHROPOMETRY

generated by fixed list — `peAnthropometry`

01 1

02 2

03 3

Weight (kg)	NUMERIC: DECIMAL `peWeight`<br>_ _ _ _ _ _ _ _ _ _ _ _ _ _ _ _ _ _ _ _
Height (cm)	NUMERIC: DECIMAL `peHeight`<br>_ _ _ _ _ _ _ _ _ _ _ _ _ _ _ _ _ _ _ _
Waste (cm)	NUMERIC: DECIMAL `peWaste`<br>_ _ _ _ _ _ _ _ _ _ _ _ _ _ _ _ _ _ _ _
Hip (cm)	NUMERIC: DECIMAL `peHip`<br>_ _ _ _ _ _ _ _ _ _ _ _ _ _ _ _ _ _ _ _

MORBIDITÉ

Roster: PHYSICAL EXAMINATION - %ROSTERTITLE%

generated by list question `membres` — `physExam`

E `false /* ...for now: email Rainer Sauerborn 10.5.2022 */`

MORBIDITÉ / PHYSICAL EXAMINATION - %ROSTERTITLE%

PHYSICAL EXAMINATION

E `!$P1P2`

	Question	Type	Variable
 V1<br>M1	Body temperature (°C)<br>`self.InRange(35,42.9)`<br>This cannot be the body temperature in degrees Celsius!	NUMERIC: DECIMAL	`peTemp`
	Tap to record time of measurement	DATE: CURRENT TIME	`peTimeBP1`
	Systolic blood pressure (mmHg)	NUMERIC: INTEGER	`pePsyst1`
	Diastolic blood pressure (mmHg)	NUMERIC: INTEGER	`pePdiast1`
	Pulse	NUMERIC: INTEGER	`pePulse1`
	VARIABLE<br>`5`	DOUBLE	`peBPinterval`
	VARIABLE<br>`/* (int) peBPinterval - (peTimeBP2 - peTimeBP1.Value.AddMinutes((double) peBPinterval)).Value.Minutes */ (int) peBPinterval - (peTimeBP2 - peTimeBP1).Value.Minutes`	LONG	`peBPelapsed2`

STATIC TEXT

Wait for %peBPinterval% minutes before continuing...

	Question	Type	Variable
 V1<br>M1	Tap to record time of 2nd measurement<br>`peTimeBP1.Value.AddMinutes((double) peBPinterval) < self`<br>Allow for %peBPinterval% minutes between measurements! Try again in %peBPelapsed2% minutes	DATE: CURRENT TIME	`peTimeBP2`
 E	Systolic blood pressure (mmHg)<br>`peTimeBP1.Value.AddMinutes((double) peBPinterval) < peTimeBP2`	NUMERIC: INTEGER	`pePsyst2`
 E	Diastolic blood pressure (mmHg)<br>`peTimeBP1.Value.AddMinutes((double) peBPinterval) < peTimeBP2`	NUMERIC: INTEGER	`pePdiast2`
 E	Pulse<br>`peTimeBP1.Value.AddMinutes((double) peBPinterval) < peTimeBP2`	NUMERIC: INTEGER	`pePulse2`
	VARIABLE<br>`(int) peBPinterval - (peTimeBP3 - peTimeBP2).Value.Minutes`	LONG	`peBPelapsed3`

STATIC TEXT

E `peTimeBP1.Value.AddMinutes((double) peBPinterval) < peTimeBP2`

Wait for %peBPinterval% minutes before continuing...

	Tap to record time of 3rd measurement	DATE: CURRENT TIME — peTimeBP3
E	peTimeBP1.Value.AddMinutes((double) peBPinterval) < peTimeBP2	
V1	peTimeBP2.Value.AddMinutes((double) peBPinterval) < self	
M1	Allow for %peBPinterval% minutes between measurements! Try again in %peBPelapsed3% minutes	
	Systolic blood pressure (mmHg)	NUMERIC: INTEGER — pePsyst3
E	peTimeBP2.Value.AddMinutes((double) peBPinterval) < peTimeBP3	
	Diastolic blood pressure (mmHg)	NUMERIC: INTEGER — pePdiast3
E	peTimeBP2.Value.AddMinutes((double) peBPinterval) < peTimeBP3	
	Pulse	NUMERIC: INTEGER — pePulse3
E	peTimeBP2.Value.AddMinutes((double) peBPinterval) < peTimeBP3	

STATIC TEXT

ANTHROPOMETRY section was here originally

Fasting plasma glucose (FPG) [mmol/L]	NUMERIC: INTEGER — peFPG
Glycated hemoglobin HbA1c [mmol/mol]	NUMERIC: INTEGER — peHbA1c
Hemoglobin (Hb) [g/dL]	NUMERIC: INTEGER — peHb

STATIC TEXT

Malaria rapid diagnostic test (RDT)

pan-Lactatdehydrogenase (pLDH)	SINGLE-SELECT — pepLDH<br>01 ○ negative<br>02 ○ positive
HRP2	SINGLE-SELECT — peHRP2<br>01 ○ negative<br>02 ○ positive

STATIC TEXT

Malaria microscopy

Malaria parasite count (MPC) per 200 white blood cells (WBC)<br>Thick film No. 1	TEXT — peMPC1
Malaria parasite count (MPC) per 200 white blood cells (WBC)<br>Thick film No. 2	TEXT — peMPC2

Plasmodium species	SINGLE-SELECT		pePlasmodium
	01	○ P. falciparum	
	02	○ P. malariae	
	03	○ P. ovale	
	04	○ P. vivax	
	09	○ Unknown	

AGRICULTURE AUX CHAMPS

E `$OK && $P1P2`

STATIC TEXT

Les travailleurs sur les champs

AGRICULTURE AUX CHAMPS

Roster: ÉPOUSE

generated by list question membres

epouses

E `parente.InList(3108,1720)`

Votre épouse %rostertitle% vous accompagne-t-elle aux champs ?	SINGLE-SELECT QA14<br>01 ○ Oui<br>00 ○ Non
Pour faire quels travaux spécifiquement ?<br>E `QA14==1`	MULTI-SELECT QA15<br>01 ☐ Préparer les champs<br>02 ☐ semer<br>03 ☐ Désherber=cultiver<br>04 ☐ Récolter<br>09 ☐ Autres
Autre travail a preciser<br>E `QA15.Contains(9)`	TEXT QA15_autre<br>..
Combien de personnes qui travaillent dans vot re champ sont des membres de votre ménage ?<br>V1 `QA17<=membres.Length`<br>M1 C'est plus que le nombre de personnes dans le ménage!	NUMERIC: INTEGER QA17<br>- - - - - - - - - - - - - - - - - - - -
Parmi ceux, combien sont des femmes ?<br>V1 `self <= QA17`<br>M1 le nombre de femmes ne peut pas être superieur au nombre total des membres du ménage travaillant dans le champs!	NUMERIC: INTEGER QA18<br>- - - - - - - - - - - - - - - - - - - -
Combien d'ouvriers journaliers travaillent dans votres champs ?<br>V1 `self<=100`<br>M1 le nombre de personne doit etre inferieur ou egal a 100	NUMERIC: INTEGER QA16<br>- - - - - - - - - - - - - - - - - - - -

STATIC TEXT

Quels des dispositifs agricoles suivants le ménage possède?

Nombre de Faucheuse<br>V1 `self<100`<br>M1 le nombre de Faucheuse doit etre compris entre 0 a 99	NUMERIC: INTEGER nbreFaucheuse<br>- - - - - - - - - - - - - - - - - - - -<br>SPECIAL VALUES<br>99 Néant

Nombre de Coupe-coupe

V1 self<100

M1 le nombre de Coupe-coupe doit etre compris entre 0 a 99

NUMERIC: INTEGER nbreCoupe

SPECIAL VALUES

99 Néant

Nombre de Grand Chariot

V1 self<100

M1 Le nombre de Grand Chariot doit etre compris entre 0 a 99

NUMERIC: INTEGER nbreGrandChario

SPECIAL VALUES

99 Néant

Nombre de petite Chariot

V1 self<100

M1 Le nombre de petit Chariot doit etre compris entre 0 a 99

NUMERIC: INTEGER nbrPetitChariot

SPECIAL VALUES

99 Néant

Nombre de Outils de mains

V1 self<100

M1 Le nombre de outil de mains doit etre comprise entre 0 a 99

NUMERIC: INTEGER nbreOutilMain

SPECIAL VALUES

99 Néant

Nombre de Tracteur

V1 self<100

M1 le nombre de tracteur doit etre compris entre 0 a 99

NUMERIC: INTEGER nbreTracteur

SPECIAL VALUES

99 Néant

Nombre de Charrues

V1 self<100

M1 le nombre de Charrue doit etre comprise entre 0 a 99

NUMERIC: INTEGER nbreCharrue

SPECIAL VALUES

99 Néant

Nombre de Houe

V1 self<100

M1 le nombre de Houe doit etre compris entre 0 a 99

NUMERIC: INTEGER nbreHoue

SPECIAL VALUES

99 Néant

Nombre de Bœuf(s) de traction

V1 self<100

M1 le nombre de Boeuf doit etre compris entre 0 a 99

NUMERIC: INTEGER NbreBoeuf

SPECIAL VALUES

99 Néant

STATIC TEXT

De la récolte en 2019 ...

Question	Réponse
Avez-vous pu nourrir votre famille avec les réco ltes de votre champs (greniers) toute l’année?	SINGLE-SELECT QA31<br>01 Oui<br>00 Non
Jusqu’à quel mois de l’année avez-vous pu nour rir votre famille avec les récoltes de votre cham ps (greniers)<br>E QA31==0	SINGLE-SELECT QA32<br>01 Janvier<br>02 Fevrier<br>03 Mars<br>04 Avril<br>05 Mai<br>06 Juin<br>07 Juillet<br>08 Aout<br>09 Septembre<br>10 Octobre<br>11 Novembre<br>12 Decembre
Avez-vous dû acheter des céréales complémen taires pour nourrir votre famille ?<br>E QA31==0	SINGLE-SELECT QA33<br>01 Oui<br>00 Non
A partir de quel mois avez-vous dû acheter des céréales complémentaires<br>E QA33==1	SINGLE-SELECT QA34<br>01 Janvier<br>02 Fevrier<br>03 Mars<br>04 Avril<br>05 Mai<br>06 Juin<br>07 Juillet<br>08 Aout<br>09 Septembre<br>10 Octobre<br>11 Novembre<br>12 Decembre

Quelles des cereales suivantes complementaire vous avez acheté pour nourrir votre famille?

E QA33==1

MULTI-SELECT: YES/NO QA35

01	☐/☐ Sorgho
02	☐/☐ Riz
03	☐/☐ Fonio
04	☐/☐ Mais
05	☐/☐ Petit mil
06	☐/☐ Blé
07	☐/☐ Haricot
08	☐/☐ Arachide
09	☐/☐ soja
10	☐/☐ Patate douce
11	☐/☐ Igname
12	☐/☐ Manioc
13	☐/☐ Banane
14	☐/☐ pomme de terre
15	☐/☐ oseille/bissap
16	☐/☐ sésame

And 1 other symbols [21]

AGRICULTURE AUX CHAMPS

Roster: DETAIL

generated by multi-select question QA35 r_nouriture

Quel est la quantité de %r_nouriture% que vous avez acheté?

V1 QA36_qtite_kg<=20000

M1 la quantité en kg semble etre tres elevé

NUMERIC: DECIMAL QA36_qtite_kg

_ _

Quel est l'unité de %rostertitle% acheté par le menage?

SINGLE-SELECT uniteCerealeAchete

01	○ Boîte de tomate
02	○ Tine
03	○ Sac grand (100kg)
04	○ Sac petit (50kg)
05	○ Kilogramme
06	○ Nombre

Quel est le prix de 1 %uniteCerealeAchete% de %r_nouriture% que vous avez acheté?

V1 /* meaningless if unit can be kg or 100kg bags QA36_prix_kg<=2000 */ true

M1 le prix semble etre elevé

NUMERIC: INTEGER QA36_prix_kg

_ _

STATIC TEXT

Attention: Dans cet enquête on demande seulement sur les cultures de la campagne précédante!

Votre menage a t-il cultivé les cultures suivants<br>a la campagne précédante?	MULTI-SELECT: YES/NO QCS<br>01 ☐/☐ Sorgho<br>02 ☐/☐ Petit mil<br>03 ☐/☐ Maïs<br>04 ☐/☐ Arachides<br>05 ☐/☐ Riz<br>06 ☐/☐ Oseille/bissap<br>07 ☐/☐ Blé<br>08 ☐/☐ Manioc<br>09 ☐/☐ Pommes de terre<br>10 ☐/☐ Cotton<br>11 ☐/☐ Sésame<br>12 ☐/☐ Fonio<br>13 ☐/☐ Haricots<br>14 ☐/☐ Petits poids<br>15 ☐/☐ Soja<br>16 ☐/☐ Patate douce<br>And 3 other symbols [22]
Combien de champs votre ménage cultive-t-il ?<br>I Un champ doit avoir: - une seule culture - ou deux ou plus cultures mél angées (associées)<br>V1 QA11<=10<br>M1 Le nombre de champss doit etre inferieur ou egal a 10	NUMERIC: INTEGER QA11<br>_ _
Listez tous les champs que le Menage Cultive<br>Donnez un nom a chaque champ<br>I Exemple: Champ de Mamadou Champ du nord Plantation des bananes<br>V1 `self.Length <= QA11`<br>M1 Mais vous avez dit qu'il y a seulement %QA11% champs! SVP, changez le número des champs.<br>V2 `fieldsToList <= 0`<br>M2 Il faut lister encore %fieldsToList% champs!	LIST `ListeChamp`<br>..
VARIABLE<br>`QA11 - ListeChamp.Length`	LONG `fieldsToList`<br>`This variable is excluded from the exported data`

LES CHAMPS DU MÉNAGE

E `$OK && $P1P2`

LES CHAMPS DU MÉNAGE

Roster: DETAILS

generated by list question `ListeChamp` — `lesChamps`

Quelle est la longueur du %lesChamps% en metres?<br>E `false`	NUMERIC: DECIMAL — `LongueurChamp`<br>_ _
Quelle est la largeur du %lesChamps% en metres?<br>E `false`	NUMERIC: DECIMAL — `largeurChamp`<br>_ _
VARIABLE<br>`LongueurChamp*largeurChamp`	DOUBLE — `SuperficieM2`
VARIABLE<br>`SuperficieM2/10000`	DOUBLE — `SuperficeHa`
Combien d'hectares a le %lesChamps%?<br>V1 `self<1000`<br>M1 Ça parait beaucoup! Vous êtes sur que ces sont des HECTARES?	NUMERIC: DECIMAL — `areaHa`<br>_ _
Quelle est la source d'eau utilisée pour arroser le champ?	SINGLE-SELECT — `sourceEau`<br>01 ○ Pluie<br>02 ○ Eau de Fleuve<br>03 ○ Puits<br>09 ○ Autre
Quelle autre source?<br>E `sourceEau==9`	TEXT — `sourceEauAutre`<br>..
Le %lesChamps% avait eu une seule culture ou plusieurs cultures associées <u>a la campagne précédante</u>?	SINGLE-SELECT — `associe`<br>01 ○ Monoculture<br>02 ○ Associé

Listez les cultures que le menage a cultivé sur le %lesChamps% a la campagne précédante<br><br>I Si la culture n'existe pas dans la liste, l'ajoutez [[ici]](QCS)<br>F QCS.Yes.Contains(@optioncode)<br>V1 `associe == 1 && self.Length <=1 || associe == 2`<br>M1 Mais vous avez dit que le champs est "Monoculture"! SVP, changez pour "Associé"	MULTI-SELECT — culturesDuChamp<br>01 ☐ Sorgho<br>02 ☐ Petit mil<br>03 ☐ Maïs<br>04 ☐ Arachides<br>05 ☐ Riz<br>06 ☐ Oseille/bissap<br>07 ☐ Blé<br>08 ☐ Manioc<br>09 ☐ Pommes de terre<br>10 ☐ Cotton<br>11 ☐ Sésame<br>12 ☐ Fonio<br>13 ☐ Haricots<br>14 ☐ Petits poids<br>15 ☐ Soja<br>16 ☐ Patate douce<br><br>And 3 other symbols [23]
Ces cultures etaient intercalée?<br><br>E `culturesDuChamp.Length > 1 &&false`	SINGLE-SELECT — intercale<br>01 ○ Oui<br>02 ○ Non

LES CHAMPS DU MÉNAGE / DETAILS

Roster: DETAILS

generated by multi-select question `culturesDuChamp` — `culturesCultivees`

Quelle est la longueur dut a la culture de %culturesCultivees% ?<br><br>I Veuillez donner la longueur en mettre<br>E `false`	NUMERIC: DECIMAL — longueurCulture<br><br>_ _ _ _ _ _ _ _ _ _ _ _ _ _ _ _ _ _ _ _
Quelle est la largeur dut a la culture de %culturesCultivees% ?<br><br>E `false`	NUMERIC: DECIMAL — largeurCulture<br><br>_ _ _ _ _ _ _ _ _ _ _ _ _ _ _ _ _ _ _ _
Quelle partie de %lesChamps% vous avez récolté de %rostertitle%?<br><br>E `intercale==1`	SINGLE-SELECT — percentChamps<br>01 ○ un quart<br>02 ○ un tiers<br>03 ○ la moitié<br>04 ○ deux tiers<br>05 ○ trois quarts
VARIABLE<br>`longueurCulture*largeurCulture`	DOUBLE — superCulturCarre
VARIABLE<br>`superCulturCarre/10000`	DOUBLE — SuperficeCultureHa

Question	Réponses
Dans quel mois avez-vous semé le %culturesCultivees%	SINGLE-SELECT — MoisSemance<br>01 ○ Janvier<br>02 ○ Fevrier<br>03 ○ Mars<br>04 ○ Avril<br>05 ○ Mai<br>06 ○ Juin<br>07 ○ Juillet<br>08 ○ Aout<br>09 ○ Septembre<br>10 ○ Octobre<br>11 ○ Novembre<br>12 ○ Decembre<br>99 ○ Ne sait pas
Dans quelle semaine avez-vous semé le %culturesCultivees%	SINGLE-SELECT — SemaineSemance<br>01 ○ 1ere semaine<br>02 ○ 2eme semaine<br>03 ○ 3eme semaine<br>04 ○ 4eme semaine<br>99 ○ Ne Sait pas
Dans quel mois avez-vous Recolté le %culturesCultivees%	SINGLE-SELECT — moisRecolt<br>01 ○ Janvier<br>02 ○ Fevrier<br>03 ○ Mars<br>04 ○ Avril<br>05 ○ Mai<br>06 ○ Juin<br>07 ○ Juillet<br>08 ○ Aout<br>09 ○ Septembre<br>10 ○ Octobre<br>11 ○ Novembre<br>12 ○ Decembre<br>99 ○ Ne sait pas
Dans quelle semaine avez-vous récolté le %culturesCultivees%	SINGLE-SELECT — SemaineRecolt<br>01 ○ 1ere semaine<br>02 ○ 2eme semaine<br>03 ○ 3eme semaine<br>04 ○ 4eme semaine<br>99 ○ Ne Sait pas

LES CULTURES SPÉCIFIQUES

E `$OK && $P1P2`

LES CULTURES SPÉCIFIQUES

Roster: DETAIL

generated by multi-select question QCS

rCulture

STATIC TEXT

E `!lesChamps.Any(c => c.culturesDuChamp.Contains(@rowcode))`

Vous avez dit que vous avez cultivez %rostertitle%,
mais il n y a aucun champ avec %rostertitle%!
SVP, indiquez %rostertitle% dans les champs ou il était cultivé!

Type de cultures<br>V1 `lesChamps.Any(c => c.culturesDuChamp.Contains(@rowcode))`<br>M1 Vous avez dit que vous avez cultivez %rostertitle%,<br>mais il n y a aucun champ avec %rostertitle%!<br>SVP, indiquez %rostertitle% [dans les champs](lesChamps) ou il était cultivé!	SINGLE-SELECT QCS_typeCulture<br>01 ○ Traditionnel<br>02 ○ OMG<br>03 ○ Autre
Quelle autre type de culture<br>E `QCS_typeCulture==3`	TEXT autreTypeCulture<br>..
Quantité total de %rCulture% récoltée par le ménage entier	NUMERIC: INTEGER qtiteToal<br>_ _ _ _ _ _ _ _ _ _ _ _ _ _ _ _ _ _ _ _
Quel est l'unité de la Quantité total de %rCulture% récoltée par le ménage	SINGLE-SELECT unite_recolte<br>01 ○ Boîte de tomate<br>02 ○ Tine<br>03 ○ Sac grand (100kg)<br>04 ○ Sac petit (50kg)<br>05 ○ Kilogramme<br>06 ○ Nombre<br>07 ○ Tonne
Le menage a t-il vendu du %rCulture% au marcher?	SINGLE-SELECT qtiteVendu<br>01 ○ Oui<br>00 ○ Non
Quelle est la quantite du %rCulture% vendu?<br>E `qtiteVendu==1`	NUMERIC: INTEGER qtiteV<br>_ _ _ _ _ _ _ _ _ _ _ _ _ _ _ _ _ _ _ _

Quel est l'unité de la quantité de %rCulture% vendu par le menage?<br><br>E qtiteVendu==1	SINGLE-SELECT unite_vendu<br>01 ○ Boîte de tomate<br>02 ○ Tine<br>03 ○ Sac grand (100kg)<br>04 ○ Sac petit (50kg)<br>05 ○ Kilogramme<br>06 ○ Nombre<br>07 ○ Tonne
Le menage a t-il acheté du %rCulture% au marcher?	SINGLE-SELECT qtiteAchete<br>01 ○ Oui<br>00 ○ Non
Quelle est la quantité de %rCulture% acheté au marché?<br><br>E qtiteAchete==1	NUMERIC: INTEGER quantie<br>- -
Quel est l'unité de %rCulture% acheté par le menage?<br><br>E qtiteAchete==1	SINGLE-SELECT uniteAchete<br>01 ○ Boîte de tomate<br>02 ○ Tine<br>03 ○ Sac grand (100kg)<br>04 ○ Sac petit (50kg)<br>05 ○ Kilogramme<br>06 ○ Nombre<br>07 ○ Tonne
Quelles difficultées liées à l'environnement avez-vous été confronté avec %rCulture%?	MULTI-SELECT stress<br>00 ☐ Aucune<br>01 ☐ Sècheresse<br>02 ☐ innondation<br>03 ☐ plante infectée<br>04 ☐ criquet pélérin<br>05 ☐ oiseaux migrateurs<br>09 ☐ Autre
Quelle autre stress environnemental?<br><br>E stress.Contains(9)	TEXT autre_stress<br>..
Quelles difficultées economique vous avez eu?	MULTI-SELECT stressEcon<br>00 ☐ Aucune<br>01 ☐ Prix d'engrais<br>02 ☐ Prix de pesticides<br>03 ☐ Prix de semances<br>05 ☐ Autre
Quelle autre dificultée?<br><br>E stressEcon.Contains(5)	TEXT stressEconAutre<br>..

Combien de mois votre ménage consomme la production (%rCulture%) avant rupture du stoc k?	NUMERIC: INTEGER dureeConsommation<br>_ _
Utilisez vous des fertilisant avec %rCulture%?	SINGLE-SELECT fertilisation<br>01 O Oui<br>00 O Non
Quel type de fertilisant<br>E fertilisation==1	SINGLE-SELECT typeFertilisant<br>01 O Traditionel (fumier)<br>02 O Artificiel(chimique)
Quel type?<br>E typeFertilisant==2	SINGLE-SELECT typeArtificie<br>01 O NPK<br>02 O Urée<br>03 O Phosphate<br>05 O Autre
Quel autre fertilisant artificiel?<br>E typeArtificie==5	TEXT autreArti<br>..
Quelle quantité de fertilisant pour %rCulture%?<br>E fertilisation==1	NUMERIC: DECIMAL quantiteFertilisant<br>_ _
Unité<br>E fertilisation==1	SINGLE-SELECT uniteFertilisant<br>01 O Kg<br>02 O Sac de 50 kg<br>03 O charrette
Ultilisez-vous d'insecticide avec %rCulture%?	SINGLE-SELECT insecticide<br>01 O Oui<br>00 O Non
%rCulture% a-t-il été attaqué par des Parasite/I nsectes/Sauterelles ?	SINGLE-SELECT sauterelle<br>01 O Oui<br>02 O Non
Quantité de la récolte perdue par Parasite/Inse ctes/Sauterelles?<br>E sauterelle==1	NUMERIC: INTEGER quantPerdue<br>_ _<br>SPECIAL VALUES<br>00 Néant

Question	Réponse	Variable
Quelle est l'unité de %rCulture% perdue?<br>E quantPerdue > 0	SINGLE-SELECT<br>01 ○ Boîte de tomate<br>02 ○ Tine<br>03 ○ Sac grand (100kg)<br>04 ○ Sac petit (50kg)<br>05 ○ Kilogramme<br>06 ○ Nombre<br>07 ○ Tonne	unitePerdue
Quantité de la récolte perdue par <u>autres cause s</u> (périssés, volés, etc.) ?	NUMERIC: INTEGER<br>- -<br>SPECIAL VALUES<br>00 Néant	quantPerdueAutre
Quelle est l'unité de %rCulture% perdue?<br>E quantPerdueAutre>0	SINGLE-SELECT<br>01 ○ Boîte de tomate<br>02 ○ Tine<br>03 ○ Sac grand (100kg)<br>04 ○ Sac petit (50kg)<br>05 ○ Kilogramme<br>06 ○ Nombre<br>07 ○ Tonne	unitePerdueAutre

LES CULTURES SPÉCIFIQUES / DETAIL

PRATIQUES D'AGRICULTURE

Question	Réponse	Variable
La culture est-elle séchée avant conservation (s tockage) ?	SINGLE-SELECT<br>01 ○ Oui<br>02 ○ Non	PA1
Où cette culture est-elle conservée (stockée) ?<br>E PA1==1	SINGLE-SELECT<br>01 ○ Entrepot<br>02 ○ Grenier<br>03 ○ Sur le champ<br>04 ○ Dans votre concession au vil lage<br>05 ○ Hangar	PA2
Comment %rCulture% est conservé (stocké) ?<br>E PA1==1	SINGLE-SELECT<br>01 ○ Décortiquée simple<br>02 ○ Sous forme de sacs de 50kg ou 100 kg<br>03 ○ Autre	PA3
Comment?<br>E PA3==3	TEXT<br>..	PA3autre

Appliquez-vous de poison (rodenticides), contr e les rongeurs dans votre grenier pour la cultu re ?	SINGLE-SELECT PA4<br>01 ◯ Oui<br>02 ◯ Non
Combien (grammes)?<br>I 1 kg = 1000 grammes<br>E PA4==1	NUMERIC: INTEGER PA5<br>_ _
Pratiquez-vous une technique de conservation d'eau?	SINGLE-SELECT PA6<br>01 ◯ Oui<br>02 ◯ Non
Dans quels champs?<br>E PA6==1	MULTI-SELECT: LINKED PA7<br>SOURCE OF CATEGORIES: TEXT LIST QUESTION LISTECHAMP

LES CULTURES SPÉCIFIQUES / DETAIL / PRATIQUES D'AGRICULTURE

Roster: TECHNIQUE

generated by list question ListeChamp — champs

E `PA7.Contains(@rowcode)`

Quelle pratique sur le %champs%?<br>(multiple réponses possible)	MULTI-SELECT PA8<br>01 ☐ Cordon pierreux<br>02 ☐ Demi-lune<br>03 ☐ Diguettes<br>04 ☐ Arrosement artificiel
Pratiquez-vous une technique de récupération des sols?	SINGLE-SELECT PA9<br>01 ◯ Oui<br>02 ◯ Non
Dans quels champs?<br>E PA9==1	MULTI-SELECT: LINKED PA10<br>SOURCE OF CATEGORIES: TEXT LIST QUESTION LISTECHAMP

LES CULTURES SPÉCIFIQUES / DETAIL / PRATIQUES D'AGRICULTURE

Roster: TECHNIQUE

generated by list question ListeChamp — champs2

E `PA10.Contains(@rowcode)`

Quelle pratique sur le %champs2%?
(multiple réponses possible)

MULTI-SELECT PA11

01 ☐ Jachère
02 ☐ Rotation des cultures (répété les cultures de façon cyclique)
03 ☐ Assolement (changer de cultures sur la même parcelle)
04 ☐ Brise vent ou haie vive (planter des plantes autour du champs comme bordure, exemple acacia)
05 ☐ Agroforesterie (associe des plantes pérennes comme anacarde aux cultures)

A quel prix avez-vous payé l'angrais?

E `rCulture.Count(x => x.fertilisation==1)>0`

NUMERIC: INTEGER prixAngrais

_ _

HANDICAPS

E `$OK1 && !$P1P2`

HANDICAPS

Roster: HANDICAP

generated by list question `membres` — `r_handicaps`

Question	Response	Variable
Est-ce que %rostertitle% a un handicap (visuel, auditif, moteur ou mental)?	SINGLE-SELECT<br>01 ○ Oui<br>02 ○ Non<br>03 ○ Ne sait pas	`hasHandicap`
Est-ce que %rostertitle% est orphelin?<br>E `parente!=3599 && mereDansMenage!=1 && age<20 && hasHandicap==1`	SINGLE-SELECT<br>01 ○ Oui<br>02 ○ Non	`orphelin`
Le père de %membre% vit dans le ménage?<br>E `parente!=3599 && hasHandicap==1 && age<20`	SINGLE-SELECT<br>01 ○ Oui<br>02 ○ Non	`hcPereDansMenage`
Qui est le père de %membre%<br>F membre[@rowcode].sexe==1 && membre[@rowcode].age > 14<br>E `hcPereDansMenage==1 && hasHandicap==1`	SINGLE-SELECT: LINKED<br>SOURCE OF CATEGORIES: ROSTER MEMBRE	`pereDuHandicape`
Nom du père<br>E `parente!=3599 && hasHandicap==1 && hcPereDansMenage==2`	TEXT<br>..	`hcNomPere`
Prénom(s) du père<br>E `parente!=3599 && hasHandicap==1 && hcPereDansMenage==2`	TEXT<br>..	`hcPrenomPere`
Profession du père<br>E `parente!=3599 && hasHandicap==1 && hcPereDansMenage==2`	TEXT<br>..	`hcProfPere`
Adresse / Tel du père<br>E `parente!=3599 && hasHandicap==1 && hcPereDansMenage==2`	TEXT<br>..	`hcAdrPere`
Nombre d'enfants scolarisés de la famille<br>E `parente!=3599 && hasHandicap==1`	NUMERIC: INTEGER<br>_ _ _ _ _ _ _ _ _ _ _ _ _ _ _ _ _ _ _ _	`hcNmbEnfScol`
Qui est la mère de %membre%<br>F membre[@rowcode].sexe==2 && membre[@rowcode].age > 11<br>E `mereDansMenage==1 && hasHandicap==1 && age<20`	SINGLE-SELECT: LINKED<br>SOURCE OF CATEGORIES: ROSTER MEMBRE	`mereDuHandicape`
Nom de la mère<br>E `mereDansMenage!=1 && hasHandicap==1 && age<20`	TEXT<br>..	`hcNomMere`

Prénom(s) de la mère<br>E mereDansMenage!=1 && hasHandicap==1 && age<20	TEXT hcPrenomMere<br>..
Profession de la mère<br>E mereDansMenage!=1 && hasHandicap==1 && age<20	TEXT hcProfMere<br>..
Adresse / Tel de la mère<br>E mereDansMenage!=1 && hasHandicap==1 && age<20	TEXT hcAdrMere<br>..
Nature de la déficience<br>E hasHandicap==1	MULTI-SELECT hcNatureDef<br>01 ☐ Handicap mental (ou déficience intellectuelle)<br>02 ☐ Handicap auditif<br>03 ☐ Handicap visuel<br>04 ☐ Aveugle<br>05 ☐ Malvoyant<br>06 ☐ Handicap moteur<br>07 ☐ Autisme et Troubles Envahissants du Développement<br>08 ☐ Handicap Psychique<br>09 ☐ Pluri handicap<br>10 ☐ Polyhandicap

HANDICAPS / HANDICAP

Roster: %ROSTERTITLE%

generated by multi-select question hcNatureDef — selectedHandicaps

Est-ce que %rostertitle% est inné ou acquis?	SINGLE-SELECT typeHandicap<br>01 ○ Inné (né avec)<br>02 ○ Acquis
Date début de %rostertitle% - Année<br>E typeHandicap==2<br>V1 self.InRange(1900, dateEntree.Value.Year) || self==9999<br>M1 L'année doit être entre 1900 et la date d'aujourd'hui	NUMERIC: INTEGER hcAnnee<br>- -<br>SPECIAL VALUES<br>9999 Ne sait pas

Date début de %rostertitle% - Mois<br><br>E typeHandicap==2 && hcAnnee!=9999	SINGLE-SELECT hcMois<br>01 Janvier<br>02 Février<br>03 Mars<br>04 Avril<br>05 Mai<br>06 Juin<br>07 Juillet<br>08 Août<br>09 Septembre<br>10 Octobre<br>11 Novembre<br>12 Décembre<br>99 Ne sait pas
Nom du répondant<br><br>I (Si la personne n'est pas dans la liste, retournez a la [liste des membre s](membres) e ajoutez cette personne!)	SINGLE-SELECT: LINKED repondantHandicaps<br>SOURCE OF CATEGORIES: ROSTER MEMBRE

ÉMIGRATION

E ($OK1 || $allLeft) && !$P1P2

Depuis %dateBasePretty% une personne a quitté le ménage?	SINGLE-SELECT<br>01 Oui<br>02 Non	avaitEmigration
Une de ces personnes a quitté le ménage depuis de %dateBasePretty%?<br>(Si la personne n'est pas dans la liste, retournez a la [liste des membres](membres) e ajoutez cette personne!)<br>E /* don't ask if all members are new! -> new household */ /* membre.Count(x=>x.preloadedMember==1)>0 */ avaitEmigration==1	MULTI-SELECT: LINKED<br>SOURCE OF CATEGORIES: ROSTER MEMBRE	anyEmigration

STATIC TEXT

E result==6 && (anyEmigration.Length < membres.Length)

Vous avez dites que tout le ménage a déménagé!
Il faut selectionner tous les membres du ménage.

Quelqu'un du ménage a l'intention de quitter le ménage?<br>E !$allLeft	SINGLE-SELECT<br>01 Oui<br>02 Non	anyIntendedEmigration
Qui a l'intention de quitter le ménage?<br>(Si la personne n'est pas dans la liste, retournez a la [liste des membres](membres) e ajoutez cette personne!)<br>E anyIntendedEmigration==1	MULTI-SELECT: LINKED<br>SOURCE OF CATEGORIES: ROSTER MEMBRE	intendedEmigration
Nom du répondant<br>E !$allLeft	SINGLE-SELECT: LINKED<br>SOURCE OF CATEGORIES: ROSTER MEMBRE	repondantEmig

ÉMIGRATION

Roster: ÉMIGRATION / DÉPLACEMENT SAISONNIER

generated by list question membres

r_emigration

E anyEmigration.Any(x=>x[0]==@rowcode) /* previously only worked with x[1]==@rowcode, now with x[0]==@rowcode as documented in public qxs!!! */

S’agit-il d’une émigration interne?<br>E false	SINGLE-SELECT<br>01 Oui<br>02 Non	emigrationInterne
A %rostertitle% émigré et est revenu(e)?	SINGLE-SELECT<br>01 Oui<br>02 Non	emigration6mois
A %rostertitle% émigré et a l'intention de revenir?<br>E emigration6mois==2	SINGLE-SELECT<br>01 Oui<br>02 Non	emigration6moisPrevu

VARIABLE	STRING	returned
`emigration6mois==1 ? " " : "prévue"`		

Date de l'émigration - Année

NUMERIC: INTEGER — anneeE

V1 `self >= dateBase.Value.Year`
M1 Impossible! %rostertitle% était encore dans le ménage en %dateBase Pretty%!
V2 `self <= dateEntree.Value.Year`
M2 Ne peut pas être à l'avenir!

____

Date de l'émigration - Mois

SINGLE-SELECT — moisE

E `anneeE!=9999`

- 01 ○ Janvier
- 02 ○ Février
- 03 ○ Mars
- 04 ○ Avril
- 05 ○ Mai
- 06 ○ Juin
- 07 ○ Juillet
- 08 ○ Août
- 09 ○ Septembre
- 10 ○ Octobre
- 11 ○ Novembre
- 12 ○ Décembre
- 99 ○ Ne sait pas

Date de l'émigration - Jour

SINGLE-SELECT — jourE

F (@optioncode==30 && moisE!=2) || (@optioncode==31 && moisE.InList(1,3,5,7,8,10,12)) || @optioncode.InRange(1,29) || @optioncode==99
E `moisE.InRange(1,12)`
V1 `new DateTime((int) anneeE,(int) moisE,jourE==99?1:(int) jourE) <= dateEntree`
M1 Date de la émigration ne peut pas être à l'avenir!

- 01 ○ 1
- 02 ○ 2
- 03 ○ 3
- 04 ○ 4
- 05 ○ 5
- 06 ○ 6
- 07 ○ 7
- 08 ○ 8
- 09 ○ 9
- 10 ○ 10
- 11 ○ 11
- 12 ○ 12
- 13 ○ 13
- 14 ○ 14
- 15 ○ 15
- 16 ○ 16

And 16 other symbols [24]

Date %returned% de Retour - Année

NUMERIC: INTEGER — anneeER

E `emigration6mois==1 || emigration6moisPrevu==1`

____

Question	Réponses
Date %returned% de Retour - Mois<br><br>E anneeER!=9999 && (emigration6mois==1 || emigration6moisPrevu==1)	SINGLE-SELECT moisER<br>01 Janvier<br>02 Février<br>03 Mars<br>04 Avril<br>05 Mai<br>06 Juin<br>07 Juillet<br>08 Août<br>09 Septembre<br>10 Octobre<br>11 Novembre<br>12 Décembre<br>99 Ne sait pas
Date %returned% de Retour - Jour<br><br>F (@optioncode==30 && moisER!=2) | | (@optioncode==31 && moisER.InList(1,3,5,7,8,10,12)) | | @optioncode.InRange(1,29) | | @optioncode==99<br>E moisER.InRange(1,12) && (emigration6mois==1 || emigration6moisPrevu==1)	SINGLE-SELECT jourER<br>01 1<br>02 2<br>03 3<br>04 4<br>05 5<br>06 6<br>07 7<br>08 8<br>09 9<br>10 10<br>11 11<br>12 12<br>13 13<br>14 14<br>15 15<br>16 16<br><br>And 16 other symbols [25]
Quelle était la destination de %rostertitle%?	SINGLE-SELECT destination<br>01 Intérieur SSDS<br>02 Au Burkina Faso<br>03 Hors Burkina Faso

**A quel village?**<br><br>I Tapez partie du nom du village, et sélectionnez de la liste<br>E destination==1	SINGLE-SELECT: COMBO BOX — villDestination<br>02 ○ Toni<br>05 ○ Boron<br>08 ○ Cissé<br>09 ○ Dankoumana<br>10 ○ Dembeléla<br>11 ○ Denissa<br>12 ○ Denissa Mossi<br>14 ○ Dionkongo<br>15 ○ Dina<br>16 ○ Dokoura<br>17 ○ Goni<br>18 ○ Kamadena<br>19 ○ Kèmena<br>21 ○ Koro<br>23 ○ Leï<br>27 ○ Ouetté<br><br>And 43 other symbols [26]
**A quel secteur?**<br><br>E destination==1<br>V1 self.InRange(0,99)<br>M1 Secteur a seulement 2 chiffres!	NUMERIC: INTEGER — secteurDestination<br><br>- -<br><br>SPECIAL VALUES<br>00 00
**A quelle concession?**<br><br>E destination==1<br>V1 self.InRange(1,9999)<br>M1 Concession a entre 1 et 4 chiffres.	NUMERIC: INTEGER — concessionDestination<br><br>- -<br><br>SPECIAL VALUES<br>9999 Ne sait pas
**A quel ménage?**<br><br>I XX - si le ménage est inconnu<br>E destination==1	TEXT — menageDestination<br><br>..

A quel lieu?

F (@optioncode<=4 | | @optioncode==99) && destination==2 | | @optioncode>4 && destination==3

E `destination>1`

SINGLE-SELECT — `destinationHorsSSDS`

- 01 ○ Ouaga
- 02 ○ Bobo
- 03 ○ Dedougou
- 05 ○ Côte d'Ivoire
- 06 ○ Mali
- 07 ○ Autre lieu en Afrique
- 08 ○ Amerique
- 09 ○ Asie
- 10 ○ Europe
- 11 ○ Australie
- 99 ○ Autre

Quelle autre destination?

E `destinationHorsSSDS==99`

TEXT — `destinationHorsSSDSautre`

....

Raison de la émigration?

E `false`

SINGLE-SELECT — `raisonEmigrationOld`

- 01 ○ Mariage
- 02 ○ Accoucher
- 03 ○ Cultiver
- 04 ○ Travail rémunéré en ville
- 05 ○ Affectation/obtention d’emploi
- 06 ○ Travail minier
- 07 ○ Travail dans les plantations
- 08 ○ Pâturage
- 09 ○ Etudier
- 10 ○ Orpaillage
- 99 ○ Autre

Quelle est la principale activité prévue de %rostertitle% a la destination?	SINGLE-SELECT `raisonEmigration`<br>01 ○ Mariage<br>02 ○ Accoucher<br>11 ○ Confiage<br>03 ○ Cultiver<br>12 ○ Suivre un parent<br>13 ○ Visiter parents/amis<br>04 ○ Travail rémunéré en ville<br>05 ○ Affectation/obtention d'emploi<br>06 ○ Travail minier<br>07 ○ Travail dans les plantations<br>08 ○ Pâturage<br>09 ○ Etudier<br>10 ○ Orpaillage<br>99 ○ Autre
Quelle autre raison?<br>E `raisonEmigration==99`	TEXT `raisonAutreE`<br>..
Causes de la émigration<br>F (raisonEmigration==3 && @optioncode.InList(1,2,3,4,99)) || (raisonEmigration==4 && @optioncode.InList(4,99)) || (raisonEmigration==6 && @optioncode.InList(1,2,4,99)) || (raisonEmigration==7 && @opt<br>And 109 other symbols [3]<br>E `!raisonEmigration.InList(1,2,5,8,9) &&false`	SINGLE-SELECT `causeEmigrationOld`<br>01 ○ Récoltes insuffisantes<br>02 ○ Saison pluvieuse inondant ou avec poches de sécheresse<br>03 ○ Recherche de terres cultivables<br>04 ○ Besoin quelconque d'argent<br>05 ○ Affectation/obtention d'emploi<br>06 ○ Mariage<br>07 ○ Accouchement<br>08 ○ Etudes<br>09 ○ Confiage<br>10 ○ Prob. conjugal<br>11 ○ Prob. de santé<br>99 ○ Autre

Quelles sont les raisons de quitter sa maison p our %raisonEmigration%?<br><br>I jusque 3 selections possible<br>F (raisonEmigration==1 && @optioncode.InList(15,12,98,99)) | | (raison Emigration==2 && @optioncode.InList(16,12,17,98,99)) | | (raisonEmi gration==11 && @optioncode.InList(4,12,14,10,16,98,99)) | | (rais<br>And 752 other symbols [4]	MULTI-SELECT causeEmigration<br>01 ☐ Récoltes insuffisantes<br>02 ☐ Saison pluvieuse inondant ou avec poches de sécheresse<br>03 ☐ Pas de terres cultivables<br>13 ☐ Pas de paturages<br>18 ☐ Pas de travail ici<br>04 ☐ Besoin quelconque d'argent<br>14 ☐ Aucune (bonne) école ici<br>15 ☐ Aventure/Liberté<br>16 ☐ Meilleurs services de santé<br>10 ☐ Prob. conjugal<br>11 ☐ Prob. de santé<br>17 ☐ Être avec famille<br>12 ☐ Insecurité<br>98 ☐ Aucune raison particulière<br>99 ☐ Autre
Quelle autre cause?<br><br>E `causeEmigration.Contains(99)`	TEXT causeAutreE<br><br>..
Quelle est la raison plus importante?<br><br>F causeEmigration.Contains(@optioncode)<br>E `causeEmigration.Length > 1`	SINGLE-SELECT mainCause<br>01 ○ Récoltes insuffisantes<br>02 ○ Saison pluvieuse inondant ou avec poches de sécheresse<br>03 ○ Pas de terres cultivables<br>13 ○ Pas de paturages<br>18 ○ Pas de travail ici<br>04 ○ Besoin quelconque d'argent<br>14 ○ Aucune (bonne) école ici<br>15 ○ Aventure/Liberté<br>16 ○ Meilleurs services de santé<br>10 ○ Prob. conjugal<br>11 ○ Prob. de santé<br>17 ○ Être avec famille<br>12 ○ Insecurité<br>98 ○ Aucune raison particulière<br>99 ○ Autre
VARIABLE<br>`causeEmigration.Length == 1 ? causeEmigration[0] : mainCause`	LONG mainCauseEmigration

IMMIGRATION

E `$OK1 && !$P1P2`

Depuis %dateBasePretty% une personne est ar rivée dans le ménage?	SINGLE-SELECT<br>01 O Oui<br>02 O Non	avaitImmigration
Selectionnez toutes les personnes qui sont arri vées dans votre ménage depuis de %dateBase Pretty%<br>- même si elles sont reparties ou ont l'intention de repartir.<br>(Si la personne n'est pas dans la liste, retournez a la [liste des membres](membres) e ajoutez cette personne!)<br>E `avaitImmigration==1`	MULTI-SELECT: LINKED<br>SOURCE OF CATEGORIES: ROSTER MEMBRE	anyImmigration
Nom du répondant	SINGLE-SELECT: LINKED<br>SOURCE OF CATEGORIES: ROSTER MEMBRE	repondantImmig

IMMIGRATION

Roster: IMMIGRÉ

generated by list question `membres` — `r_immigration`

E `anyImmigration.Any(x=>x[0]==@rowcode) /* what the heck?! In PUBLIC EXAMPLE HH Workers Demo it works with x[0]==@rowcode but not here!!! */ && !$P1P2`

VARIABLE<br>`r_immigration[@rowcode].rowname`	STRING	immigrant
Combien de fois %membre% a immigré?	NUMERIC: INTEGER<br>_ _ _ _ _ _ _ _ _ _ _ _ _ _ _ _ _ _ _ _	nbImmigrations

IMMIGRATION / IMMIGRÉ

Roster: IMMIGRATION

generated by numeric question `nbImmigrations` — `r_immigration_episode`

%membre% a résidé au moins 2 mois?	SINGLE-SELECT<br>01 O Oui<br>02 O Non	plusDe2Mois

IMMIGRATION / IMMIGRÉ / IMMIGRATION

IMMIGRATION %MEMBRE%

E `/* only if born before previous census (ageInMonths1 > (CenturyMonthCode(dateEntree.Value.Month, dateEntree.Value.Year) - CenturyMonthCode(dateBase.Value.Month, dateBase.Value.Year) - ((dateEntree.Va` And 135 other symbols [2]

STATIC TEXT

%membre% n'etait pas dans le ménage au census anterieur.
Donc %membre% doit avoir immigré après.

S'agit-il d'une migration interne?<br>E `false`	SINGLE-SELECT<br>01 O Oui<br>02 O Non	migrationInterne

	A %membre% immigré et est reparti(e)?	SINGLE-SELECT immigration6mois<br>01 Oui<br>02 Non
E	A %membre% immigré et a l'intention de repar tir?<br>`immigration6mois==2`	SINGLE-SELECT immigration6moisPrevu<br>01 Oui<br>02 Non
	VARIABLE<br>`immigration6mois==1 ? " " : "prévue"`	STRING left
	%membre% a-t-il déjà été enregistré?	SINGLE-SELECT isEnregistre<br>01 Oui<br>02 Non
	%membre% avait-il vécu dans ce ménage avant ?	SINGLE-SELECT formerMember<br>01 Oui<br>02 Non
V1<br>M1<br>V2<br>M2	Date de l'immigration - Année<br>`self >= dateBase.Value.Year`<br>Seulement immigrés depuis de %dateBasePretty%!<br>`self <= dateEntree.Value.Year`<br>Ne peut pas être à l'avenir!	NUMERIC: INTEGER anneeI<br>_ _ _ _ _ _ _ _ _ _ _ _ _ _ _ _ _ _ _ _
E	Date de l'immigration - Mois<br>`anneeI!=9999`	SINGLE-SELECT moisI<br>01 Janvier<br>02 Février<br>03 Mars<br>04 Avril<br>05 Mai<br>06 Juin<br>07 Juillet<br>08 Août<br>09 Septembre<br>10 Octobre<br>11 Novembre<br>12 Décembre<br>99 Ne sait pas

Date de l'immigration - Jour

SINGLE-SELECT jourI

F (@optioncode==30 && moisI!=2) || (@optioncode==31 && moisI.InList(1,3,5,7,8,10,12)) || @optioncode.InRange(1,29) || @optioncode==99

E `moisI.InRange(1,12)`

V1 `new DateTime((int) anneeI,(int) moisI,jourI==99?1:(int) jourI) <= dateEntree`

M1 Date de la immigration ne peut pas être à l'avenir!

01	1
02	2
03	3
04	4
05	5
06	6
07	7
08	8
09	9
10	10
11	11
12	12
13	13
14	14
15	15
16	16

And 16 other symbols [27]

Date %left% de Départ - Année

NUMERIC: INTEGER anneeIR

E `immigration6mois==1 || immigration6moisPrevu==1`

_ _

Date %left% de Départ - Mois

SINGLE-SELECT moisIR

E `anneeIR!=9999 && (immigration6mois==1 || immigration6moisPrevu==1)`

01	Janvier
02	Février
03	Mars
04	Avril
05	Mai
06	Juin
07	Juillet
08	Août
09	Septembre
10	Octobre
11	Novembre
12	Décembre
99	Ne sait pas

Date %left% de Départ - Jour

F (@optioncode==30 && moisIR!=2) | | (@optioncode==31 && moisIR.InList(1,3,5,7,8,10,12)) | | @optioncode.InRange(1,29) | | @optioncode==99

E `moisIR.InRange(1,12) && (immigration6mois==1 || immigration6moisPrevu==1)`

SINGLE-SELECT — `jourIR`

01	1
02	2
03	3
04	4
05	5
06	6
07	7
08	8
09	9
10	10
11	11
12	12
13	13
14	14
15	15
16	16

And 16 other symbols [28]

Quelle est la provenance de %membre%?

SINGLE-SELECT — `provenance`

01	Intérieur SSDS
02	Au Burkina Faso
03	Hors Burkina Faso

De quel village?

I Tapez partie du nom du village, et sélectionnez de la liste

E `provenance==1`

SINGLE-SELECT: COMBO BOX — `villProvenance`

02	Toni
05	Boron
08	Cissé
09	Dankoumana
10	Dembeléla
11	Denissa
12	Denissa Mossi
14	Dionkongo
15	Dina
16	Dokoura
17	Goni
18	Kamadena
19	Kèmena
21	Koro
23	Leï
27	Ouetté

And 43 other symbols [29]

De quel secteur?

E `provenance==1`
V1 `self.InRange(0,99)`
M1 Secteur a seulement 2 chiffres!

NUMERIC: INTEGER — `secteurProvenance`

SPECIAL VALUES

00	00

De quelle concession?

E `provenance==1`
V1 `self.InRange(1,9999)`
M1 Concession a entre 1 et 4 chiffres.

NUMERIC: INTEGER — `concessionProvenance`

SPECIAL VALUES

9999	Ne sait pas

De quel ménage?

I XX - si le ménage est inconnu
E `provenance==1`

TEXT — `menageProvenance`

De quel lieu?

F (@optioncode<=4 | | @optioncode==99) && provenance==2 | | @optioncode>4 && provenance==3
E `provenance>1`

SINGLE-SELECT — `provenanceHorsSSDS`

01	Ouaga
02	Bobo
03	Dedougou
05	Côte d'Ivoire
06	Mali
07	Autre lieu en Afrique
08	Amerique
09	Asie
10	Europe
11	Australie
99	Autre

Quelle autre provenance?

E `provenanceHorsSSDS==99`

TEXT — `provenanceHorsSSDSautre`

Raison/motif de la immigration?

E `false`

SINGLE-SELECT — `raisonMigration`

01	Mariage
02	Accoucher
03	Cultiver
04	Travail rémunéré en ville
05	Affectation/obtention d’emploi
06	Travail minier
07	Travail dans les plantations
08	Pâturage
09	Etudier
10	Orpaillage
99	Autre

Quelle est la principale activité pour laquelle %i mmigrant% est venu?	SINGLE-SELECT raisonImmigration<br>01 ○ Mariage<br>02 ○ Accoucher<br>11 ○ Confiage<br>03 ○ Cultiver<br>12 ○ Suivre un parent<br>13 ○ Visiter parents/amis<br>04 ○ Travail rémunéré en ville<br>05 ○ Affectation/obtention d'emp loi<br>06 ○ Travail minier<br>07 ○ Travail dans les plantations<br>08 ○ Pâturage<br>09 ○ Etudier<br>10 ○ Orpaillage<br>99 ○ Autre
Quelle autre activité?<br>E raisonImmigration==99	TEXT raisonAutreI<br>..
Causes de la immigration<br>F (raisonMigration==3 && @optioncode.InList(1,2,3,4,99)) || (raisonMigration==4 && @optioncode.InList(4,99)) || (raisonMigration==6 && @optioncode.InList(1,2,4,99)) || (raisonMigration==7 && @optionc And 103 other symbols [5]<br>E !raisonMigration.InList(1,2,5,8,9) &&false	SINGLE-SELECT causeImmigration<br>01 ○ Récoltes insuffisantes<br>02 ○ Saison pluvieuse inondant ou avec poches de sécheresse<br>03 ○ Recherche de terres cultivables<br>04 ○ Besoin quelconque d'argent<br>05 ○ Affectation/obtention d'emp loi<br>06 ○ Mariage<br>07 ○ Accouchement<br>08 ○ Etudes<br>09 ○ Confiage<br>10 ○ Prob. conjugal<br>11 ○ Prob. de santé<br>99 ○ Autre
Quelle autre cause?<br>E causeImmigration==99	TEXT causeAutreI<br>..

Quelles sont les raisons de quitter sa maison pour %raisonImmigration%?	MULTI-SELECT causesImmigration
I jusque 3 selections possible<br>F (raisonImmigration==1 && @optioncode.InList(15,12,98,99)) | | (raisonImmigration==2 && @optioncode.InList(16,12,17,98,99)) | | (raisonImmigration==11 && @optioncode.InList(4,12,14,10,16,98,99)) | | (r<br>And 766 other symbols [6]	01 ☐ Récoltes insuffisantes<br>02 ☐ Saison pluvieuse inondant ou avec poches de sécheresse<br>03 ☐ Pas de terres cultivables<br>13 ☐ Pas de paturages<br>18 ☐ Pas de travail ici<br>04 ☐ Besoin quelconque d'argent<br>14 ☐ Aucune (bonne) école ici<br>15 ☐ Aventure/Liberté<br>16 ☐ Meilleurs services de santé<br>10 ☐ Prob. conjugal<br>11 ☐ Prob. de santé<br>17 ☐ Être avec famille<br>12 ☐ Insecurité<br>98 ☐ Aucune raison particulière<br>99 ☐ Autre

DÉCÈS

E `($OK1 || $allDead) && !$P1P2`

Y-a-t-il eu un ou plusieurs décès dans votre mé nage depuis de %dateBasePretty%?<br>E `membres.Length>0`	SINGLE-SELECT — anyDeces<br>01 ○ Oui<br>02 ○ Non
Selectionnez les décès:<br>(Si la personne n'est pas dans la liste, retournez a la [liste des membres](membres) e ajoutez cette personne!)<br>E `anyDeces==1`	MULTI-SELECT: LINKED — deces<br>SOURCE OF CATEGORIES: TEXT LIST QUESTION MEMBRES

DÉCÈS

Roster: DÉCÈS

generated by list question `membres` — r_deces

E `deces.Contains(@rowcode)`

Date de Décès - Année<br>V1 `self >= dateBase.Value.Year`<br>M1 Seulement décès depuis de %dateBasePretty%!<br>Désactivez %rostertitle% dans la liste de décès!<br>V2 `self <= dateEntree.Value.Year`<br>M2 Ne peut pas être dans l'avenir!	NUMERIC: INTEGER — anneeD<br>_ _
Date de Décès - Mois<br>E `anneeD!=9999`	SINGLE-SELECT — moisD<br>01 ○ Janvier<br>02 ○ Février<br>03 ○ Mars<br>04 ○ Avril<br>05 ○ Mai<br>06 ○ Juin<br>07 ○ Juillet<br>08 ○ Août<br>09 ○ Septembre<br>10 ○ Octobre<br>11 ○ Novembre<br>12 ○ Décembre<br>99 ○ Ne sait pas

Date de Décès - Jour

F (@optioncode==30 && moisD!=2) || (@optioncode==31 && moisD.InList(1,3,5,7,8,10,12)) || @optioncode.InRange(1,29) || @optioncode==99

E `moisD.InRange(1,12)`

V1 `new DateTime((int) anneeD,(int) moisD,jourD==99?1:(int) jourD) <= dateEntree`

M1 Date de décès ne peut pas être à l'avenir!

SINGLE-SELECT jourD

01	O 1
02	O 2
03	O 3
04	O 4
05	O 5
06	O 6
07	O 7
08	O 8
09	O 9
10	O 10
11	O 11
12	O 12
13	O 13
14	O 14
15	O 15
16	O 16

And 16 other symbols [30]

VARIABLE

```
CenturyMonthCode(dateEntree.Value.Month, dateEntree.Value.Year) - // if don't know month, use current month CenturyMonthCode((moisD==99?dateEntree.Value.Month:(int) moisD), (int) anneeD) // if don't k
```

And 119 other symbols [3]

LONG months_Deceased

STATIC TEXT

E `false`

Deceased since %months_Deceased% months

STATIC TEXT

E `months_Deceased > timeSinceLast`

Seulement décès depuis de %dateBasePretty%!
Désactivez %rostertitle% dans la liste de décès!

Lieu Décès

SINGLE-SELECT lieuDeces

01	O Formation sanitaire
02	O Domicile
03	O Route/brousse
04	O Autre

Quel autre lieu?

E `lieuDeces==4`

TEXT lieuDeces_autre

....

Quelle est la cause apparente du décès de %rostertitle%	SINGLE-SELECT<br>01 ○ une maladie<br>02 ○ un accident<br>03 ○ mort naturelle<br>09 ○ autre cause<br>88 ○ Ne sais pas	causeDeces
Quelle autre cause?<br>E `causeDeces==9`	TEXT<br>..	causeDecesAutre

STATIC TEXT

E `result==7 && (deces.Length < membres.Length)`

Vous avez dites que tout le ménage est décédé!
Il faut selectionner tous les membres du ménage.

Nom du répondant<br>E `anyDeces==1 && (deces.Length < membres.Length)`	SINGLE-SELECT: LINKED<br>SOURCE OF CATEGORIES: ROSTER MEMBRE	repondantDeces

TEST GPS

STATIC TEXT

Vous pouvez utiliser ce test
pour verifier le GPS da la tablette
ou pour fair une mésure de GPS pour un Collègue,
par example si son GPS ne marche pas.

Test de GPS<br><br>I Utilisez, par exemple, pour faire une mesure GPS pour un collègue.	GPS testgps<br><br>________________<br>N<br>________________<br>W<br>________________<br>A
VARIABLE<br>`testgps.Latitude.ToString ("#.#####")`	STRING testLat
VARIABLE<br>`testgps.Longitude.ToString ("#.#####")`	STRING testLon

STATIC TEXT

E `IsAnswered(testgps)`

Latitude: %testLat%

STATIC TEXT

E `IsAnswered(testgps)`

Longitude: %testLon%

APPENDIX A — ENABLING CONDITIONS

[1] : Nouveau-né %membre%

Enablement Condition:

```
/* only if born after previous census */
ageInMonths1 <=
(CenturyMonthCode(dateEntree.Value.Month, dateEntree.Value.Year) -
CenturyMonthCode(dateBase.Value.Month, dateBase.Value.Year)
- ((dateEntree.Value.Day < dateBase.Value.Day)?1:0))
```

[2] : Immigration %membre%

Enablement Condition:

```
/* only if born before previous census
(ageInMonths1 >
(CenturyMonthCode(dateEntree.Value.Month, dateEntree.Value.Year) -
CenturyMonthCode(dateBase.Value.Month, dateBase.Value.Year)
- ((dateEntree.Value.Day < dateBase.Value.Day)?1:0)))
*/
/* and not part of the household during the last visit
&& preloadedMember!=1
*/
plusDe2Mois==1
```

APPENDIX B — VALIDATION CONDITIONS AND MESSAGES

[1] qn_height1: Height1 today in centimeter (###,#)
Validation Condition:

```
// WHO growth standards height for age (0 to 60 months) by sex

sexe==2 ? self.InRange(Height[(int)age_bracket].minF,Height[(int)age_bracket].maxF)
        : self.InRange(Height[(int)age_bracket].minM,Height[(int)age_bracket].maxM)


/*
(self>=43.6 && self<=72.5 && qn04==2 && qn_agec_mo>=0 && qn_agec_mo<=6)
// || (self>=44.2 && self<=74.0 && qn04==1 && qn_agec_mo>=0 && qn_agec_mo<=6)
 || (self.InRange(44.2, 74.0) && qn04==1 && qn_agec_mo>=0 && qn_agec_mo<=6)
 || (self>=60.3 && self<=81.7 && qn04==2 && qn_agec_mo>=7 && qn_agec_mo<=12)
 || (self>=62.7 && self<=82.9 && qn04==1 && qn_agec_mo>=7 && qn_agec_mo<=12)
 || (self>=67.3 && self<=96.1 && qn04==2 && qn_agec_mo>=13 && qn_agec_mo<=24)
 || (self>=69.6 && self<=97.0 && qn04==1 && qn_agec_mo>=13 && qn_agec_mo<=24)
 || (self>=76.8 && self<=106.5 && qn04==2 && qn_agec_mo>=25 && qn_agec_mo<=36)
 || (self>=78.6 && self<=107.2 && qn04==1 && qn_agec_mo>=25 && qn_agec_mo<=36)
 || (self>=84.2 && self<=115.7 && qn04==2 && qn_agec_mo>=37 && qn_agec_mo<=48)
 || (self>=85.5 && self<=115.9 && qn04==1 && qn_agec_mo>=37 && qn_agec_mo<=48)
 || (self>=90.3 && self<=123.7 && qn04==2 && qn_agec_mo>=49 && qn_agec_mo<=60)
 || (self>=91.2 && self<=123.9 && qn04==1 && qn_agec_mo>=49 && qn_agec_mo<=60)
*/
```

Validation Message: Please check height - is this value correct?

[2] qn_weight1: Weight1 today in kilogram (##,#)
Validation Condition:

```
// WHO growth standards weight for age (0 to 5 years) by sex
sexe==2 ? self.InRange(Weight[(int)age_bracket].minF,Weight[(int)age_bracket].maxF)
        : self.InRange(Weight[(int)age_bracket].minM,Weight[(int)age_bracket].maxM)
/*
(self.InRange(2,10.6) && qn04==2 && qn_agec_mo>=0 && qn_agec_mo<=6)
 || (self.InRange(2.1, 10.9) && qn04==1 && qn_agec_mo>=0 && qn_agec_mo<=6)
 || (self.InRange(5.3,13.1) && qn04==2 && qn_agec_mo>=7 && qn_agec_mo<=12)
 || (self.InRange(5.9, 13.3) && qn04==1 && qn_agec_mo>=7 && qn_agec_mo<=12)
 || (self.InRange(6.4,17.0) && qn04==2 && qn_agec_mo>=13 && qn_agec_mo<=24)
 || (self.InRange(7.1,17.1) && qn04==1 && qn_agec_mo>=13 && qn_agec_mo<=24)
 || (self.InRange(8.2,20.9) && qn04==2 && qn_agec_mo>=25 && qn_agec_mo<=36)
 || (self.InRange(8.8,20.7) && qn04==1 && qn_agec_mo>=25 && qn_agec_mo<=36)
 || (self.InRange(9.7,25.2) && qn04==2 && qn_agec_mo>=37 && qn_agec_mo<=48)
 || (self.InRange(10.1,24.2) && qn04==1 && qn_agec_mo>=37 && qn_agec_mo<=48)
 || (self.InRange(11.0,29.5) && qn04==2 && qn_agec_mo>=49 && qn_agec_mo<=60)
 || (self.InRange(11.3,27.9) && qn04==1 && qn_agec_mo>=49 && qn_agec_mo<=60)
*/
```

Validation Message: Please check weight - is this value correct?

APPENDIX C — CATEGORIES

[1] villages

Categories: 2: Toni , 5: Boron , 8: Cissé , 9: Dankoumana , 10: Dembeléla , 11: Denissa , 12: Denissa Mossi , 14: Dionkongo , 15: Dina , 16: Dokoura , 17: Goni , 18: Kamadena , 19: Kèmena , 21: Koro , 23: Leï , 27: Ouetté , 28: Pa , 29: Sampopo , 30: Seriba , 31: Sien , 33: Sobon , 34: Solimana , 36: Tebéré , 37: Tonséré , 39: Tissi , 40: Dara , 41: Bankoumani , 42: Nouna , 43: Babekolon , 44: Bagala , 45: Biron badala , 46: Bissau , 47: Bokuy , 48: Damandigui , 49: Hinkuy , 51: Kansara , 52: Kéréna , 53: Konkuini , 54: Korédougou , 55 : Moinsi , 56: Mourdié , 57: Sèrè , 58: Soin , 59: Tonkoroni , 1: Barakuy , 3: Biron-Bobo , 4: Biron-Marka , 6: Bouni/Bouné , 7: Bourasso , 13: Diamasso , 20: Kodougou , 22: Labarani , 24: Lekuy , 25: Lemini , 26: Nokuy , 32: Sikoro , 35: Sirakoro/Sirakorosso , 38: Zanakuy , 50: Kamiankoro

[2] vill: Village

Categories: 2: Toni , 5: Boron , 8: Cissé , 9: Dankoumana , 10: Dembeléla , 11: Denissa , 12: Denissa Mossi , 14: Dionkongo , 15: Dina , 16: Dokoura , 17: Goni , 18: Kamadena , 19: Kèmena , 21: Koro , 23: Leï , 27: Ouetté , 28: Pa , 29: Sampopo , 30: Seriba , 31: Sien , 33: Sobon , 34: Solimana , 36: Tebéré , 37: Tonséré , 39: Tissi , 40: Dara , 41: Bankoumani , 42: Nouna , 43: Babekolon , 44: Bagala , 45: Biron badala , 46: Bissau , 47: Bokuy , 48: Damandigui , 49: Hinkuy , 51: Kansara , 52: Kéréna , 53: Konkuini , 54: Korédougou , 55 : Moinsi , 56: Mourdié , 57: Sèrè , 58: Soin , 59: Tonkoroni , 1: Barakuy , 3: Biron-Bobo , 4: Biron-Marka , 6: Bouni/Bouné , 7: Bourasso , 13: Diamasso , 20: Kodougou , 22: Labarani , 24: Lekuy , 25: Lemini , 26: Nokuy , 32: Sikoro , 35: Sirakoro/Sirakorosso , 38: Zanakuy , 50: Kamiankoro

[3] q200: Le ménage possède-t-il un de ces équipements?

Categories: 201: RADIO , 202: TELEVISEUR , 203: LECTEUR VIDEO/CD/DVD , 204: TELEPHONE FIXE , 205: TELEPHONE PORTABLE , 218: MOTO , 219: TRICYCLE , 220: BICYCLETTE (VÉLO) , 206: REFRIGERATEUR / CONGELATEUR , 208: PLAQUE A GAZ , 209: CUISINIÈRE , 210: RECHAUD A PETROLE / GAZ , 211: PLAQUE ELECTRIQUE , 212: PLAQUE SOLAIRE , 213: GROUPE ELECTROGENE , 214: LAMPE SOLAIRE , 215: LAMPE A PETROLE , 216: LAMPE A GAZ , 224: CLÉ USB , 225: BOOMER (BOOMBOX) , 217: VOITURE , 221: CHARRETTE (2 roues) , 226: CHARIOT (4 roues) , 222: ORDINATEUR BUREAU , 223: ORDINATEUR PORTABLE

[4] parente: Lien de parenté avec CM

Categories: 1546: Chef de ménage , 3108: Epouse/époux , 1720: Coépouse , 3599: Fils/fille , 3042: Enfant adopté/confié , 3826: Frère/sœur , 10157: Père /mère , 10275: Petit Fils/fille , 8913: Neveu/nièce , 9804: Oncle/tante , 3047: Enfant conjoint(e) , 4446: Grand-père/grand-mère , 4173: Gendre/belle fille , 823: Beau-frère/belle sœur , 539: Autre parent , 531: Aucun lien , 9271: Ne sais pas

[5] jourN: Date de Naissance - Jour

Categories: 1: 1 , 2: 2 , 3: 3 , 4: 4 , 5: 5 , 6: 6 , 7: 7 , 8: 8 , 9: 9 , 10: 10 , 11: 11 , 12: 12 , 13: 13 , 14: 14 , 15: 15 , 16: 16 , 17: 17 , 18: 18 , 19: 19 , 20: 20 , 21: 21 , 22: 22 , 23: 23 , 24: 24 , 25: 25 , 26: 26 , 27: 27 , 28: 28 , 29: 29 , 30: 30 , 31: 31 , 99: Ne sait pas

[6] classe: Dernière classe frequenté

Categories: 1: PS1 , 2: PS2 , 3: PS3 , 4: PR1 , 5: PR2 , 6: PR3 , 7: PR4 , 8: PR5 , 9: PR6 , 10: SE1 , 11: SE2 , 12: SE3 , 13: SE4 , 14: SE5 , 15: SE6 , 16: SE7 , 17: SU1 , 18: SU2 , 19: SU3 , 20: SU4 , 21: SU5 , 22: SU6 , 23: SU7 , 24: SU8 , 25: SU9 , 26: CA1 , 27: CA2 , 28: CA3 , 29: BE1 , 30: BE2 , 31: CB1 , 32: CB2 , 33: CB3 , 34: CB4

[7] prevClasse: Dernière classe frequenté

Categories: 1: PS1 , 2: PS2 , 3: PS3 , 4: PR1 , 5: PR2 , 6: PR3 , 7: PR4 , 8: PR5 , 9: PR6 , 10: SE1 , 11: SE2 , 12: SE3 , 13: SE4 , 14: SE5 , 15: SE6 , 16: SE7 , 17: SU1 , 18: SU2 , 19: SU3 , 20: SU4 , 21: SU5 , 22: SU6 , 23: SU7 , 24: SU8 , 25: SU9 , 26: CA1 , 27: CA2 , 28: CA3 , 29: BE1 , 30: BE2 , 31: CB1 , 32: CB2 , 33: CB3 , 34: CB4

[8] occupation: Occupation principale

Categories: 1: Agriculture , 2: Apiculture , 3: Berger , 4: Boucher , 5: Cathéchiste , 6: Chauffeur , 7: Coiffeur , 8: Commerce , 9: Cordonnier , 10: Cuisinier , 11: Dolotière , 12: Elève/Écolier/Etudiant , 13: Elevage , 14: Forgeron/ FGR=FOR , 15: Fileuse , 16: Fonc. public , 17: Gardien , 18: Griot , 19: Jardinier , 20: Macon(nerie) , 21: Marabout , 22: Ménagère , 23: Pêcheur , 24: Potier(ie) , 25: Puisatier , 26: Tailleur , 27: Teinturier , 28: Tisstérand , 29: Vanier , 30: Neant , 31: Marab./Macon , 32: Autre Fonct , 33: Aucun , 34: Sécretaire , 35: Gendarme , 36: Policier/ POL=PLC , 37: Mécanicien , 38: Garagiste , 39: Ouvrier , 40: Comptable , 41: Agent de Recherche , 42: Missionnaire , 43: Gestionnaire , 44: Entrepreneur , 45: Boulanger , 46: Bijoutier , 47: Plombier , 48: Institutiteur , 49: Profess/secondaire , 50: Profess/supérieur , 51: Médecin , 52: Infirmier , 53: Matrone , 54: Sage femme , 55: Agt santé villageois , 56: Pharmacien , 57: Guérisseur , 58: Policier , 59: Menuisier , 60: Apprenti , 61: Cçant de mchde diver , 62: Cçant légume/condime , 63: Cçant de bétail , 64: Cçant de céréales , 65: Cçant de Volailles , 66: Cçant Beurre karité , 67: Cçant tissus/pagnes , 90: Retraité , 95: Défaut , 97: Non applic , 98: Sans info , 68: Autre artisan , 69: Travailleur du Privé , 99: ne sait pas - NSP , 96: Autres

[9] residPereVill: A quel village?

Categories: 2: Toni , 5: Boron , 8: Cissé , 9: Dankoumana , 10: Dembeléla , 11: Denissa , 12: Denissa Mossi , 14: Dionkongo , 15: Dina , 16: Dokoura , 17: Goni , 18: Kamadena , 19: Kèmena , 21: Koro , 23: Leï , 27: Ouetté , 28: Pa , 29: Sampopo , 30: Seriba , 31: Sien , 33: Sobon , 34: Solimana , 36: Tebéré , 37: Tonséré , 39: Tissi , 40: Dara , 41: Bankoumani , 42: Nouna , 43: Babekolon , 44: Bagala , 45: Biron badala , 46: Bissau , 47: Bokuy , 48: Damandigui , 49: Hinkuy , 51: Kansara , 52: Kéréna , 53: Konkuini , 54: Korédougou , 55 : Moinsi , 56: Mourdié , 57: Sèrè , 58: Soin , 59: Tonkoroni , 1: Barakuy , 3: Biron-Bobo , 4: Biron-Marka , 6: Bouni/Bouné , 7: Bourasso , 13: Diamasso , 20: Kodougou , 22: Labarani , 24: Lekuy , 25: Lemini , 26: Nokuy , 32: Sikoro , 35: Sirakoro/Sirakorosso , 38: Zanakuy , 50: Kamiankoro

[10] villAccouchement: A quel village?

Categories: 2: Toni , 5: Boron , 8: Cissé , 9: Dankoumana , 10: Dembeléla , 11: Denissa , 12: Denissa Mossi , 14: Dionkongo , 15: Dina , 16: Dokoura , 17: Goni , 18: Kamadena , 19: Kèmena , 21: Koro , 23: Leï , 27: Ouetté , 28: Pa , 29: Sampopo , 30: Seriba , 31: Sien ,

33 : Sobon , 34 : Solimana , 36 : Tebéré , 37 : Tonséré , 39 : Tissi , 40 : Dara , 41 : Bankoumani , 42 : Nouna , 43 : Babekolon , 44 : Bagala , 45 : Biron badala , 46 : Bissau , 47 : Bokuy , 48 : Damandigui , 49 : Hinkuy , 51 : Kansara , 52 : Kéréna , 53 : Konkuini , 54 : Korédougou , 55 : Moinsi , 56 : Mourdié , 57 : Sèrè , 58 : Soin , 59 : Tonkoroni , 1 : Barakuy , 3 : Biron-Bobo , 4 : Biron-Marka , 6 : Bouni/Bouné , 7 : Bourass o , 13 : Diamasso , 20 : Kodougou , 22 : Labarani , 24 : Lekuy , 25 : Lemini , 26 : Nokuy , 32 : Sikoro , 35 : Sirakoro/Sirakorosso , 38 : Zanaku y , 50 : Kamiankoro

[11] produit: %rmembre% a cultivé l'un des produits agricoles énoncés au cours de la campagne passée?
Categories: 1 : Arachides et sous produits , 2 : Calebasses (entières , 3 : Chou , 4 : Coton et sous produits , 5 : Fonio , 6 : Cultures fruitières , 7 : Igname , 8 : Mais et sous produits , 9 : Mil/ sorgho et sous produits , 10 : Niébé et sous produits , 11 : Oignon , 12 : Oseille et sous produit s , 13 : Pastèque , 14 : Pomme de terre , 15 : Pois de terre et sous produits , 16 : Riz et sous produits , 17 : Sésame , 18 : Patate douce , 19 : Tabac , 20 : Tomate , 21 : Salade , 22 : Aubergine , 23 : Courgette , 24 : Comcombre , 25 : Autres cultures

[12] produitVendu: %rmembre% a vendu des produits agropastoraux énoncés au cours du mois passé et des 5 mois précédent le mois passé.?
Categories: 1 : Arachides et sous produits , 2 : Calebasses (par moitié) , 3 : Chou , 4 : Coton et sous produits , 5 : Fonio , 6 : Cultures fruitière s , 7 : Igname , 8 : Mais et sous produits , 9 : Sorgho et sous produits , 38 : Petit mil et sous produits , 10 : Niébé et sous produits , 11 : Oigno n , 12 : Oseille et sous produits , 13 : Pastèque , 14 : Pomme de terre , 15 : Pois de terre et sous-produits , 16 : Riz et sous produits , 17 : Sés ame , 18 : Patate douce , 19 : Tabac , 20 : Tomates , 21 : Salade , 22 : Aubergine , 23 : Courgette , 24 : Comcombre , 25 : Autres cultures , 2 6 : Karité (amandes et beurre) , 27 : Néré (grains et soumbala , 28 : Autres produits de la cueillette (tamarin, miel et de la chasse , 29 : Vente de bovins (famille du bœuf) , 30 : Vente de caprins (famille de la chèvre) , 31 : Vente d'ovins (famille du mouton) , 32 : Vente de porcins (famille du porc) , 33 : Vente d'asins (famille de l'âne) , 34 : Vente de volailles (poulets, pintades, canards) , 35 : Vente d'autres animaux (pigeons, lapin s, ...) , 36 : Vente de produits d'animaux (œufs, lait, peau, etc.) , 37 : Vente de produits de pêche (poissons, ...)

[13] depenseMembre: %rmembre% avez vous dépensé de l'argent pour l'un ou plusieurs des motifs suivants dans les 6 mois passé?
Categories: 1 : Loyer , 2 : Electricité , 3 : Téléphone , 4 : Matériaux de construction (tôles, briques, seccos, ...) , 5 : Boissons ou eau , 6 : Vivres (céréales, condiments, ...) , 7 : Ticket de transport ou Carburant , 8 : Achat ou réparation d'un moyen de transport , 9 : Scolarisation, Education , Alphabétisation , 10 : Vêtements , 11 : Soins médicaux , 12 : Assurance maladie (AMBC) , 13 : Engrais, pesticide , 14 : Outils agricoles ou fra is de réparation d'outils agricoles , 15 : Animaux , 16 : Dépenses religieuses , 17 : Fêtes, cérémonies (funérailles, mariage, ...) , 18 : Pétrole , 1 9 : Tabac, cola , 20 : Envoi d'argent à un parent / ami , 21 : Remboursement de crédit(s) , 22 : Autres motifs , 23 : Néant

[14] codeMaladieChronique: Enqueteur: Selectionnez la maladie dans la liste
Categories: 100 : Maladies ou infections pulmonaires chroniques (poumons) , 101 : Asthme , 102 : Tuberculose , 103 : Maladies ou infections oculaires chroniques (œil) , 104 : Conjonctivite chronique , 105 : Borgne , 106 : Carence en vitamine A (avitaminose A) , 107 : Cataracte , 108 : Cécité / Aveugle , 109 : Cécité nocturne ou crépusculaire , 110 : Onchocercose (cécité des rivières) , 111 : Tension de l'œil (glaucome) , 112 : Trachome , 113 : Maladies ou infections dermatologiques chroniques (peau) , 114 : Gale , 115 : Teignes (dermatophyties) , 116 : Maladies ou infections gastro-intestinales chroniques (estomac, intestins) , 117 : Dysentérie chronique , 118 : Amibiase , 119 : Shigellose , 120 : Maladi es ou infections rénales chroniques (reins) , 121 : Glomérulonéphrite chronique , 122 : Insuffisance rénale chronique , 123 : Syndrome néphro tique , 124 : Maladies cardiologiques (cœur) , 125 : Insuffisance cardiaque , 126 : Maladies ou infections hépatiques (foie) , 127 : Cirrhose , 1 28 : Hépatite , 129 : Maladies mentales , 130 : Folie , 131 : Cancers , 132 : Cancer (tumeur maligne) du foie , 133 : Cancer (tumeur maligne) de la langue , 134 : Cancer (tumeur maligne) de la peau , 135 : Cancer (tumeur maligne) des organes génitaux , 136 : Cancer (tumeur malign e) des voies respiratoires , 137 : Cancer (tumeur maligne) du sein , 138 : Cancer du cerveau (tumeur maligne cérébrale) , 139 : Cancer du sys tème digestif (tumeur maligne digestive) , 140 : Autres cancers : A préciser dans Col. 4 , 141 : Alcoolisme , 142 : Bilharziose , 143 : Diabète , 1 44 : Drépanocytose , 145 : Eléphantiasis (filariose lymphatique) , 146 : Epilepsie , 147 : Gangrène , 148 : Goitre (nodule thyroïdien) , 149 : H ernie , 150 : Hypertension artérielle , 151 : Hypertension artérielle de la grossesse , 152 : Lèpre , 153 : Maladie du sommeil (trypanosomiase africaine) , 154 : Malnutrition de l'enfant ou de l'adulte , 155 : Malnutrition du nouveau né , 156 : Paralysie , 157 : Pré-éclampsie , 158 : Rhum atisme , 159 : Séquelles d'une morsure de scorpion , 160 : Séquelles d'une morsure de serpent , 161 : Sida , 162 : Stérilité , 163 : Surdité , 16 4 : Ver de guinée (dracunculose) , 165 : Autres maladies chroniques , 999 : Ne sait pas

[15] jourMC: Jour
Categories: 1 : 1 , 2 : 2 , 3 : 3 , 4 : 4 , 5 : 5 , 6 : 6 , 7 : 7 , 8 : 8 , 9 : 9 , 10 : 10 , 11 : 11 , 12 : 12 , 13 : 13 , 14 : 14 , 15 : 15 , 16 : 16 , 17 : 17 , 18 : 18 , 19 : 19 , 20 : 20 , 21 : 21 , 22 : 22 , 23 : 23 , 24 : 24 , 25 : 25 , 26 : 26 , 27 : 27 , 28 : 28 , 29 : 29 , 30 : 30 , 31 : 31 , 99 : Ne sait p as

[16] codeMaladieAigue: Selectionnez la maladie
Categories: 200 : Maladies ou infections pulmonaires aiguës (poumons) , 201 : Bronchiolite , 202 : Bronchite , 203 : Pneumonie , 204 : Malad ies ou infections respiratoires hautes aiguës (gorge, nez, oreilles) , 205 : Coqueluche , 206 : Grippe , 207 : Infections de la gorge (laryngite, an gine ...) , 208 : Infections de l'oreille (otites, ...) , 209 : Infections du nez , 210 : Oreillons , 211 : Rhume , 212 : Maladies ou infections oculaires aigues (œil) , 213 : Conjonctivite à chlamydiae appelée 'Apollo' , 214 : Conjonctivite aigue , 215 : Trachome , 216 : Maladies ou infections der matologiques aiguës (peau) , 217 : Gale , 218 : Teignes (dermatophyties) , 219 : Maladies ou infections gastro-intestinales aiguës (estomac, in testins) , 220 : Dysentérie aiguë , 221 : Amibiase , 222 : Choléra , 223 : Hémorragie gastro-intestinale , 224 : Shigellose , 225 : Maladies ou i nfections rénales aiguës (reins) , 226 : Glomérulonéphrite aiguë , 227 : Insuffisance rénale aiguë , 228 : Maladies ou infections aigues de la fe mme enceinte , 229 : Convulsions (éclampsie) , 230 : Hémorragie avant l'accouchement , 231 : Autres complications avant l'accouchement , 2 32 : Accouchement difficile (dystocie) , 233 : Travail prolongé lors de l'accouchement , 234 : Autres complications au cours de l'accouchement , 235 : Hémorragie après l'accouchement , 236 : Fièvre suite à l'accouchement (puerpérale) , 237 : Infection inflammatoire de l'utérus (endo métrite) , 238 : Autres complications après l'accouchement , 239 : Complications d'avortement , 240 : Complications d'une grossesse jumelair e ou multiple , 241 : Maladies ou infections aigues du nouveau né , 242 : Asphyxie du nouveau né à la naissance , 243 : Hémorragie de l'ombili c du nouveau né , 244 : Infection de l'ombilic du nouveau né , 245 : Jaunisse (ictère) du nouveau né , 246 : Malformation du nouveau né , 247 : Mort né , 248 : Petit poids de naissance , 249 : Prématuré , 250 : Souffrance du nouveau né à la naissance , 251 : Tétanos du nouveau né , 25 2 : Autres maladies du nouveau né , 253 : Accident vasculaire cérébral (AVC) , 254 : Arrêt cardiaque / Crise cardiaque (infarctus cardiaque) , 2 55 : Bilharziose , 256 : Fièvre jaune , 257 : Fièvre typhoïde , 258 : Gangrène , 259 : Hernie , 260 : Intoxication alimentaire , 261 : Intoxication d'origine inconnue , 262 : Intoxication par un poison / Empoisonnement , 263 : Méningite , 264 : Morsure de serpent , 265 : Occlusion intestin ale , 266 : Œdème aigue du poumon , 267 : Paludisme de type non précisé (on ne sait pas si simple ou grave) , 268 : Paludisme grave confirm

é par un examen biologique au laboratoire , 269: Paludisme grave non confirmé par un examen biologique au laboratoire , 270: Paludisme simple confirmé par un examen biologique au laboratoire , 271: Paludisme simple non confirmé par un examen biologique au laboratoire , 272: Piqûre de scorpion , 273: Piqûre d'insecte , 274: Polio (poliomyélite) , 275: Rage , 276: Rougeole , 277: Tétanos (enfant et adulte) , 278: Traumatisme par accident , 279: Varicelle , 280: Autre maladie aiguë , 999: Ne sait pas

[17] jourMA: Jour
Categories: 1: 1 , 2: 2 , 3: 3 , 4: 4 , 5: 5 , 6: 6 , 7: 7 , 8: 8 , 9: 9 , 10: 10 , 11: 11 , 12: 12 , 13: 13 , 14: 14 , 15: 15 , 16: 16 , 17: 17 , 18: 18 , 19: 19 , 20: 20 , 21: 21 , 22: 22 , 23: 23 , 24: 24 , 25: 25 , 26: 26 , 27: 27 , 28: 28 , 29: 29 , 30: 30 , 31: 31 , 99: Ne sait pas

[18] jourCrisesMACesse: Jour
Categories: 1: 1 , 2: 2 , 3: 3 , 4: 4 , 5: 5 , 6: 6 , 7: 7 , 8: 8 , 9: 9 , 10: 10 , 11: 11 , 12: 12 , 13: 13 , 14: 14 , 15: 15 , 16: 16 , 17: 17 , 18: 18 , 19: 19 , 20: 20 , 21: 21 , 22: 22 , 23: 23 , 24: 24 , 25: 25 , 26: 26 , 27: 27 , 28: 28 , 29: 29 , 30: 30 , 31: 31 , 99: Ne sait pas

[19] symptoms: De quels symptômes souffrez-vous à cause de votre maladie chronique?
Categories: 0: NON APPLICABLE , 1: Abcès , 2: Anémie , 3: Bouche fermée, crispée , 4: Boutons , 5: Boutons sur tout le corps , 6: Constipation , 7: Convulsions , 8: Corps chaud , 9: Corps enflé (œdème général) , 10: Coup rejeté en arrière , 11: Démangeaisons (prurit) , 12: Déshydratation , 13: Diarrhée aigue , 14: Diarrhée chronique , 15: Difficultés à respirer (dyspnée) , 16: Douleur au moment des selles , 17: Douleur en urinant , 18: Douleurs du bas ventre (douleurs pelviennes) , 19: Ecoulement urétral chez l'homme , 20: Evanouissement , 21: Faiblesse , 22: Fatigue (asthénie) , 23: Fièvre , 24: Fièvre intermittente , 25: Fièvre très forte , 26: Fissure anale , 27: Frisson , 28: Hémorrhoïdes , 29: Hoquet , 30: Jambe, pied enflée (œdème du pied, de la jambe) , 31: Jaunisse (ictère) , 32: Mal à la bouche , 33: Mal à la gorge , 34: Mal à la tête (céphalée) , 35: Mal à l'oreille , 36: Mal au foie , 37: Mal au rein , 38: Mal au ventre , 39: Mal aux côtes , 40: Mal aux dents , 41: Mal aux os et/ou articulations , 42: Mal aux yeux , 43: Mal de cœur , 44: Mal de dos , 45: Manque ou perte d'appétit , 46: Ne jouait plus , 47: Ne tétait plus , 48: Nez bouché , 49: Nez qui coule , 50: Palpitation du cœur , 51: Perte de poids , 52: Pertes vaginales blanches de la femme , 53: Plaie aiguë , 54: Plaie chronique , 55: Plaies sur les organes génitaux de l'homme ou de la femme , 56: Raideur du coup , 57: Règles douloureuses , 58: Respiration bruyante , 59: Respiration rapide , 60: Saignement vaginal , 61: Sang dans les crachats (hémoptysie) , 62: Sang dans les selles , 63: Sang dans les urines (hématurie) , 64: Sensation de chaleur , 65: Sensation de froid , 66: Toux , 67: Toux grasse , 68: Toux sèche , 69: Transpiration , 70: Troubles de la vision (difficultés pour voir) , 71: Troubles mentaux , 72: Vertiges , 73: Visage enflé (œdème du visage) , 74: Vomissements , 75: Yeux jaunes , 76: Yeux larmoyants , 77: Yeux rouges , 78: Autres symptômes

[20] symptomsMA: Quels symptômes avez-vous ressenti au cours de cette maladie?
Categories: 0: NON APPLICABLE , 1: Abcès , 2: Anémie , 3: Bouche fermée, crispée , 4: Boutons , 5: Boutons sur tout le corps , 6: Constipation , 7: Convulsions , 8: Corps chaud , 9: Corps enflé (œdème général) , 10: Coup rejeté en arrière , 11: Démangeaisons (prurit) , 12: Déshydratation , 13: Diarrhée aigue , 14: Diarrhée chronique , 15: Difficultés à respirer (dyspnée) , 16: Douleur au moment des selles , 17: Douleur en urinant , 18: Douleurs du bas ventre (douleurs pelviennes) , 19: Ecoulement urétral chez l'homme , 20: Evanouissement , 21: Faiblesse , 22: Fatigue (asthénie) , 23: Fièvre , 24: Fièvre intermittente , 25: Fièvre très forte , 26: Fissure anale , 27: Frisson , 28: Hémorrhoïdes , 29: Hoquet , 30: Jambe, pied enflée (œdème du pied, de la jambe) , 31: Jaunisse (ictère) , 32: Mal à la bouche , 33: Mal à la gorge , 34: Mal à la tête (céphalée) , 35: Mal à l'oreille , 36: Mal au foie , 37: Mal au rein , 38: Mal au ventre , 39: Mal aux côtes , 40: Mal aux dents , 41: Mal aux os et/ou articulations , 42: Mal aux yeux , 43: Mal de cœur , 44: Mal de dos , 45: Manque ou perte d'appétit , 46: Ne jouait plus , 47: Ne tétait plus , 48: Nez bouché , 49: Nez qui coule , 50: Palpitation du cœur , 51: Perte de poids , 52: Pertes vaginales blanches de la femme , 53: Plaie aiguë , 54: Plaie chronique , 55: Plaies sur les organes génitaux de l'homme ou de la femme , 56: Raideur du coup , 57: Règles douloureuses , 58: Respiration bruyante , 59: Respiration rapide , 60: Saignement vaginal , 61: Sang dans les crachats (hémoptysie) , 62: Sang dans les selles , 63: Sang dans les urines (hématurie) , 64: Sensation de chaleur , 65: Sensation de froid , 66: Toux , 67: Toux grasse , 68: Toux sèche , 69: Transpiration , 70: Troubles de la vision (difficultés pour voir) , 71: Troubles mentaux , 72: Vertiges , 73: Visage enflé (œdème du visage) , 74: Vomissements , 75: Yeux jaunes , 76: Yeux larmoyants , 77: Yeux rouges , 78: Autres symptômes

[21] QA35: Quelles des cereales suivantes complementaire vous avez acheté pour nourrir votre famille?
Categories: 1: Sorgho , 2: Riz , 3: Fonio , 4: Mais , 5: Petit mil , 6: Blé , 7: Haricot , 8: Arachide , 9: soja , 10: Patate douce , 11: Igname , 12: Manioc , 13: Banane , 14: pomme de terre , 15: oseille/bissap , 16: sésame , 17: produits maraichers

[22] QCS: Votre menage a t-il cultivé les cultures suivants <u>a la campagne précédante</u>?
Categories: 1: Sorgho , 2: Petit mil , 3: Maïs , 4: Arachides , 5: Riz , 6: Oseille/bissap , 7: Blé , 8: Manioc , 9: Pommes de terre , 10: Cotton , 11: Sésame , 12: Fonio , 13: Haricots , 14: Petits poids , 15: Soja , 16: Patate douce , 17: Igname , 18: Banane , 19: Produits maraichers

[23] culturesDuChamp: Listez les cultures que le menage a cultivé sur le %lesChamps% <u>a la campagne précédante</u>
Categories: 1: Sorgho , 2: Petit mil , 3: Maïs , 4: Arachides , 5: Riz , 6: Oseille/bissap , 7: Blé , 8: Manioc , 9: Pommes de terre , 10: Cotton , 11: Sésame , 12: Fonio , 13: Haricots , 14: Petits poids , 15: Soja , 16: Patate douce , 17: Igname , 18: Banane , 19: Produits maraichers

[24] jourE: Date de l'émigration - Jour
Categories: 1: 1 , 2: 2 , 3: 3 , 4: 4 , 5: 5 , 6: 6 , 7: 7 , 8: 8 , 9: 9 , 10: 10 , 11: 11 , 12: 12 , 13: 13 , 14: 14 , 15: 15 , 16: 16 , 17: 17 , 18: 18 , 19: 19 , 20: 20 , 21: 21 , 22: 22 , 23: 23 , 24: 24 , 25: 25 , 26: 26 , 27: 27 , 28: 28 , 29: 29 , 30: 30 , 31: 31 , 99: Ne sait pas

[25] jourER: Date %returned% de Retour - Jour
Categories: 1: 1 , 2: 2 , 3: 3 , 4: 4 , 5: 5 , 6: 6 , 7: 7 , 8: 8 , 9: 9 , 10: 10 , 11: 11 , 12: 12 , 13: 13 , 14: 14 , 15: 15 , 16: 16 , 17: 17 , 18: 18 , 19: 19 , 20: 20 , 21: 21 , 22: 22 , 23: 23 , 24: 24 , 25: 25 , 26: 26 , 27: 27 , 28: 28 , 29: 29 , 30: 30 , 31: 31 , 99: Ne sait pas

[26] villDestination: A quel village?
Categories: 2: Toni , 5: Boron , 8: Cissé , 9: Dankoumana , 10: Dembeléla , 11: Denissa , 12: Denissa Mossi , 14: Dionkongo , 15: Dina

, 16: Dokoura , 17: Goni , 18: Kamadena , 19: Kèmena , 21: Koro , 23: Leï , 27: Ouetté , 28: Pa , 29: Sampopo , 30: Seriba , 31: Sien , 33: Sobon , 34: Solimana , 36: Tebéré , 37: Tonséré , 39: Tissi , 40: Dara , 41: Bankoumani , 42: Nouna , 43: Babekolon , 44: Bagala , 45: Biron badala , 46: Bissau , 47: Bokuy , 48: Damandigui , 49: Hinkuy , 51: Kansara , 52: Kéréna , 53: Konkuini , 54: Korédougou , 55 : Moinsi , 56: Mourdié , 57: Sèrè , 58: Soin , 59: Tonkoroni , 1: Barakuy , 3: Biron-Bobo , 4: Biron-Marka , 6: Bouni/Bouné , 7: Bourasso , 13: Diamasso , 20: Kodougou , 22: Labarani , 24: Lekuy , 25: Lemini , 26: Nokuy , 32: Sikoro , 35: Sirakoro/Sirakorosso , 38: Zanakuy , 50: Kamiankoro

[27] jourI: Date de l'immigration - Jour
Categories: 1: 1 , 2: 2 , 3: 3 , 4: 4 , 5: 5 , 6: 6 , 7: 7 , 8: 8 , 9: 9 , 10: 10 , 11: 11 , 12: 12 , 13: 13 , 14: 14 , 15: 15 , 16: 16 , 17: 17 , 18: 18 , 19: 19 , 20: 20 , 21: 21 , 22: 22 , 23: 23 , 24: 24 , 25: 25 , 26: 26 , 27: 27 , 28: 28 , 29: 29 , 30: 30 , 31: 31 , 99: Ne sait pas

[28] jourIR: Date %left% de Départ - Jour
Categories: 1: 1 , 2: 2 , 3: 3 , 4: 4 , 5: 5 , 6: 6 , 7: 7 , 8: 8 , 9: 9 , 10: 10 , 11: 11 , 12: 12 , 13: 13 , 14: 14 , 15: 15 , 16: 16 , 17: 17 , 18: 18 , 19: 19 , 20: 20 , 21: 21 , 22: 22 , 23: 23 , 24: 24 , 25: 25 , 26: 26 , 27: 27 , 28: 28 , 29: 29 , 30: 30 , 31: 31 , 99: Ne sait pas

[29] villProvenance: De quel village?
Categories: 2: Toni , 5: Boron , 8: Cissé , 9: Dankoumana , 10: Dembeléla , 11: Denissa , 12: Denissa Mossi , 14: Dionkongo , 15: Dina , 16: Dokoura , 17: Goni , 18: Kamadena , 19: Kèmena , 21: Koro , 23: Leï , 27: Ouetté , 28: Pa , 29: Sampopo , 30: Seriba , 31: Sien , 33: Sobon , 34: Solimana , 36: Tebéré , 37: Tonséré , 39: Tissi , 40: Dara , 41: Bankoumani , 42: Nouna , 43: Babekolon , 44: Bagala , 45: Biron badala , 46: Bissau , 47: Bokuy , 48: Damandigui , 49: Hinkuy , 51: Kansara , 52: Kéréna , 53: Konkuini , 54: Korédougou , 55 : Moinsi , 56: Mourdié , 57: Sèrè , 58: Soin , 59: Tonkoroni , 1: Barakuy , 3: Biron-Bobo , 4: Biron-Marka , 6: Bouni/Bouné , 7: Bourasso , 13: Diamasso , 20: Kodougou , 22: Labarani , 24: Lekuy , 25: Lemini , 26: Nokuy , 32: Sikoro , 35: Sirakoro/Sirakorosso , 38: Zanakuy , 50: Kamiankoro

[30] jourD: Date de Décès - Jour
Categories: 1: 1 , 2: 2 , 3: 3 , 4: 4 , 5: 5 , 6: 6 , 7: 7 , 8: 8 , 9: 9 , 10: 10 , 11: 11 , 12: 12 , 13: 13 , 14: 14 , 15: 15 , 16: 16 , 17: 17 , 18: 18 , 19: 19 , 20: 20 , 21: 21 , 22: 22 , 23: 23 , 24: 24 , 25: 25 , 26: 26 , 27: 27 , 28: 28 , 29: 29 , 30: 30 , 31: 31 , 99: Ne sait pas

APPENDIX D — VARIABLES

[1] age:

IsAnswered(reportedAge) ? reportedAge : (moisN==99 ? FullYearsBetween(new DateTime((int) anneeN,7,1), dateEntree) : (jourN==99 ? FullYearsBetween(new DateTime((int) anneeN,(int) moisN,15), dateEntree) : FullYearsBetween(new DateTime((int) anneeN,(int) moisN,(int) jourN), dateEntree)))

[2] ageInMonths1:

IsAnswered(reportedAge) ? (long) reportedAge*12 : CenturyMonthCode(dateEntree.Value.Month, dateEntree.Value.Year) - // if don't know month, use June CenturyMonthCode((moisN==99?6:(int) moisN), (int) anneeN) // if don't know day, use 15th - ((dateEntree.Value.Day < (jourN==99 || moisN==99 ? 15 : (int)jourN))?1:0)

[3] months_Deceased:

CenturyMonthCode(dateEntree.Value.Month, dateEntree.Value.Year) - // if don't know month, use current month CenturyMonthCode((moisD==99?dateEntree.Value.Month:(int) moisD), (int) anneeD) // if don't know day, use current day - ((dateEntree.Value.Day < (jourD==99 || moisD==99 ? dateEntree.Value.Day : (int)jourD))?1:0)

APPENDIX E — CATEGORIES FILTERS

[1] classe: Dernière classe frequenté

niveauEducation==1 ? @optioncode.InRange(1,3) : niveauEducation==2 ? @optioncode.InRange(4,13) : niveauEducation==3 ? @optioncode.InRange(14,16) : niveauEducation==4 ? @optioncode.InRange(17,25) : niveauEducation==5 ? @optioncode.InRange(26,30) : niveauEducation==6 ? @optioncode.InRange(31,34) : false

[2] prevClasse: Dernière classe frequenté

/* niveauEducation==1 ? @optioncode.InRange(1,3) : niveauEducation==2 ? @optioncode.InRange(4,13) : niveauEducation==3 ? @optioncode.InRange(14,16) : niveauEducation==4 ? @optioncode.InRange(17,25) : niveauEducation==5 ? @optioncode.InRange(26,30) : niveauEducation==6 ? @optioncode.InRange(31,34) : false */ true

[3] causeEmigrationOld: Causes de la émigration

(raisonEmigration==3 && @optioncode.InList(1,2,3,4,99)) || (raisonEmigration==4 && @optioncode.InList(4,99)) || (raisonEmigration==6 && @optioncode.InList(1,2,4,99)) || (raisonEmigration==7 && @optioncode.InList(1,2,4,99)) || (raisonEmigration==10 && @optioncode.InList(1,2,4,99)) || raisonEmigration==99

[4] causeEmigration: Quelles sont les raisons de quitter sa maison pour %raisonEmigration%?

(raisonEmigration==1 && @optioncode.InList(15,12,98,99)) || (raisonEmigration==2 && @optioncode.InList(16,12,17,98,99)) || (raisonEmigration==11 && @optioncode.InList(4,12,14,10,16,98,99)) || (raisonEmigration==3 && @optioncode.InList(1,2,3,4,12,13,16,17,12,98,99)) || (raisonEmigration==12 && @optioncode.InList(12,15,17,98,99)) || (raisonEmigration==13 && @optioncode.InList(10,11,12,17,98,99)) || (raisonEmigration==4 && @optioncode.InList(1,3,4,10,11,12,17,18,98,99)) || (raisonEmigration==5 && @optioncode.InList(1,3,4,10,11,12,17,18,98,99)) || (raisonEmigration==6 && @optioncode.InList(1,3,4,10,11,12,17,18,98,99)) || (raisonEmigration==7 && @optioncode.InList(1,3,4,10,11,12,17,18,98,99)) || (raisonEmigration==10 && @optioncode.InList(1,3,4,10,11,12,17,18,98,99)) || (raisonEmigration==8 && @optioncode.InList(1,2,3,4,13,16,17,12,98,99)) || (raisonEmigration==9 && @optioncode.InList(12,14,15,16,17,98,99)) || raisonEmigration==99

[5] causeImmigration: Causes de la immigration

(raisonMigration==3 && @optioncode.InList(1,2,3,4,99)) || (raisonMigration==4 && @optioncode.InList(4,99)) || (raisonMigration==6 && @optioncode.InList(1,2,4,99)) || (raisonMigration==7 && @optioncode.InList(1,2,4,99)) || (raisonMigration==10 && @optioncode.InList(1,2,4,99)) || raisonMigration==99

[6] causesImmigration: Quelles sont les raisons de quitter sa maison pour %raisonImmigration%?

(raisonImmigration==1 && @optioncode.InList(15,12,98,99)) || (raisonImmigration==2 && @optioncode.InList(16,12,17,98,99)) || (raisonImmigration==11 && @optioncode.InList(4,12,14,10,16,98,99)) || (raisonImmigration==3 && @optioncode.InList(1,2,3,4,12,13,16,17,12,98,99)) || (raisonImmigration==12 && @optioncode.InList(12,15,17,98,99)) || (raisonImmigration==13 && @optioncode.InList(10,11,12,17,98,99)) || (raisonImmigration==4 && @optioncode.InList(1,3,4,10,11,12,17,18,98,99)) || (raisonImmigration==5 && @optioncode.InList(1,3,4,10,11,12,17,18,98,99)) || (raisonImmigration==6 && @optioncode.InList(1,3,4,10,11,12,17,18,98,99)) || (raisonImmigration==7 && @optioncode.InList(1,3,4,10,11,12,17,18,98,99)) || (raisonImmigration==10 && @optioncode.InList(1,3,4,10,11,12,17,18,98,99)) || (raisonImmigration==8 && @optioncode.InList(1,2,3,4,13,16,17,12,98,99)) || (raisonImmigration==9 && @optioncode.InList(12,14,15,16,17,98,99)) || raisonImmigration==99

LEGEND

Legend and structure of information in this file

Name of section

Enabling condition for this section

Question title

Type of question, scope

Answer options

Variable name

SECTION 5: OTHER INCOME SOURCES

E s4_other_sources_which.Contains(98)

Duis aute irure dolor in reprehenderit in voluptate velit esse cillum dolore eu fugiat nulla pariatur?

MULTI-SELECT
SCOPE: PREFILLED

s4_rel_leaders_other

I **This refers to family relations**
E s3_time_other > 0
V1 s4_rel_leaders_which.Contains(98)
M1 **Can not be itself**
V2 (s3_time_other_breeding_advice <= (50 - s3_time_art_insem_advice)) ||s3_time_other_breeding_advice == 0
M2 **This person is not in the list**
F optioncode != s5_ignored_option_code

01 ☐ Community animal health workers
02 ☐ Private
03 ☐ Government
04 ☐ Livestock keepers association
05 ☐ NGO

And 5 other [13]

Additional information:

"I" — Question instruction
"E" — Enabling condition
"V1" — Validation condition №1
"M1" — Message for validation №1
"F" — Filter in Categorical questions

Link to full set in appendix

Breadcrumbs

Type or roster

Roster Title

CHAPTER 3 IDENTIFICATION /
Roster: **LEADER RELATION DETAILS**
generated by fixed list:

01 Ward Livestock Officer
02 Village Livestock Officer
99 Other (specify)

List items
